# Dorsal root ganglia control nociceptive input to the central nervous system

**DOI:** 10.1371/journal.pbio.3001958

**Published:** 2023-01-05

**Authors:** Han Hao, Rosmaliza Ramli, Caixue Wang, Chao Liu, Shihab Shah, Pierce Mullen, Varinder Lall, Frederick Jones, Jicheng Shao, Hailin Zhang, David B. Jaffe, Nikita Gamper, Xiaona Du

**Affiliations:** 1 Department of Pharmacology, Hebei Medical University; The Key Laboratory of Neural and Vascular Biology, Ministry of Education, China; The Key Laboratory of New Drug Pharmacology and Toxicology, Hebei Province; Shijiazhuang, China; 2 Faculty of Biological Sciences, University of Leeds, Leeds, United Kingdom; 3 School of Dental Sciences, Universiti Sains Malaysia, Kelantan, Malaysia; 4 Department of Animal Care, Hebei Medical University; The Key Laboratory of Experimental Animal, Hebei Province; Shijiazhuang, China; 5 Department of Neuroscience, Developmental and Regenerative Biology, The University of Texas at San Antonio, San Antonio, Texas, United States of America; University of Notre Dame, Center for Stem Cells and Regenerative Medicine, UNITED STATES

## Abstract

Accumulating observations suggest that peripheral somatosensory ganglia may regulate nociceptive transmission, yet direct evidence is sparse. Here, in experiments on rats and mice, we show that the peripheral afferent nociceptive information undergoes dynamic filtering within the dorsal root ganglion (DRG) and suggest that this filtering occurs at the axonal bifurcations (t-junctions). Using synchronous in vivo electrophysiological recordings from the peripheral and central processes of sensory neurons (in the spinal nerve and dorsal root), ganglionic transplantation of GABAergic progenitor cells, and optogenetics, we demonstrate existence of tonic and dynamic filtering of action potentials traveling through the DRG. Filtering induced by focal application of GABA or optogenetic GABA release from the DRG-transplanted GABAergic progenitor cells was specific to nociceptive fibers. Light-sheet imaging and computer modeling demonstrated that, compared to other somatosensory fiber types, nociceptors have shorter stem axons, making somatic control over t-junctional filtering more efficient. Optogenetically induced GABA release within DRG from the transplanted GABAergic cells enhanced filtering and alleviated hypersensitivity to noxious stimulation produced by chronic inflammation and neuropathic injury in vivo. These findings support “gating” of pain information by DRGs and suggest new therapeutic approaches for pain relief.

## Abstract 2

Information transmitted along the peripheral somatosensory nerves can be actively modified at the dorsal root ganglion (DRG). This study demonstrates the existence of tonic and dynamic GABAergic filtering of action potentials traveling through the DRG, with therapeutic implications for pain relief.

## Introduction

Current understanding of the somatosensory information processing largely assumes that peripheral nerves faithfully deliver peripherally born action potentials to the spinal cord. The first synapse in the dorsal horn of the spinal cord is assumed to be the first major integration point for action potentials generated at the periphery. Such a view is represented by the Gate Control Theory of pain [1] and its subsequent refinements and modifications [2–4]. While it has been proposed that information processing is more efficient the earlier it begins within the sensory pathway [5–7], the absence of true synaptic connections or interneurons within peripheral somatosensory nerves and ganglia reasonably led researchers to dismiss them as possible information processing sites. Despite this, growing evidence suggests that a degree of crosstalk between the peripheral fibers [8,9] or sensory neuron somata [10–12] might exist. Moreover, there is substantial experimental evidence that action potentials propagating from the peripheral nerve endings of nociceptive nerve terminals to the spinal cord can fail (or be “filtered”) at axonal bifurcation points (t-junctions) within the dorsal root ganglion (DRG) [13–19].

An intrinsic GABAergic signaling system within DRGs was recently proposed as a modulator of ganglionic filtering [15]. Yet, our understanding of how information can be modified within DRGs still remains sparse. Here, we obtained direct in vivo evidence for ganglionic filtering mediated by the GABA signaling system and assessed if such filtering can be exploited to control pain. Using in vivo electrophysiological recordings from the peripheral and central processes of sensory neurons (in the spinal nerve (SN) and dorsal root (DR)), optogenetic manipulations, stem axon morphometry, and biophysical modeling, we demonstrate that the DRG is a bona fide processing device controlling and modifying nociceptive signaling into the central nervous system (CNS). These findings support the existence of a “peripheral gate” in somatosensory system and suggest new ways of how sensory ganglia can be targeted for pain control.

## Results

### Action potentials induced by the excitation of peripheral nerve endings are filtered within the DRG

**Fig 1:**
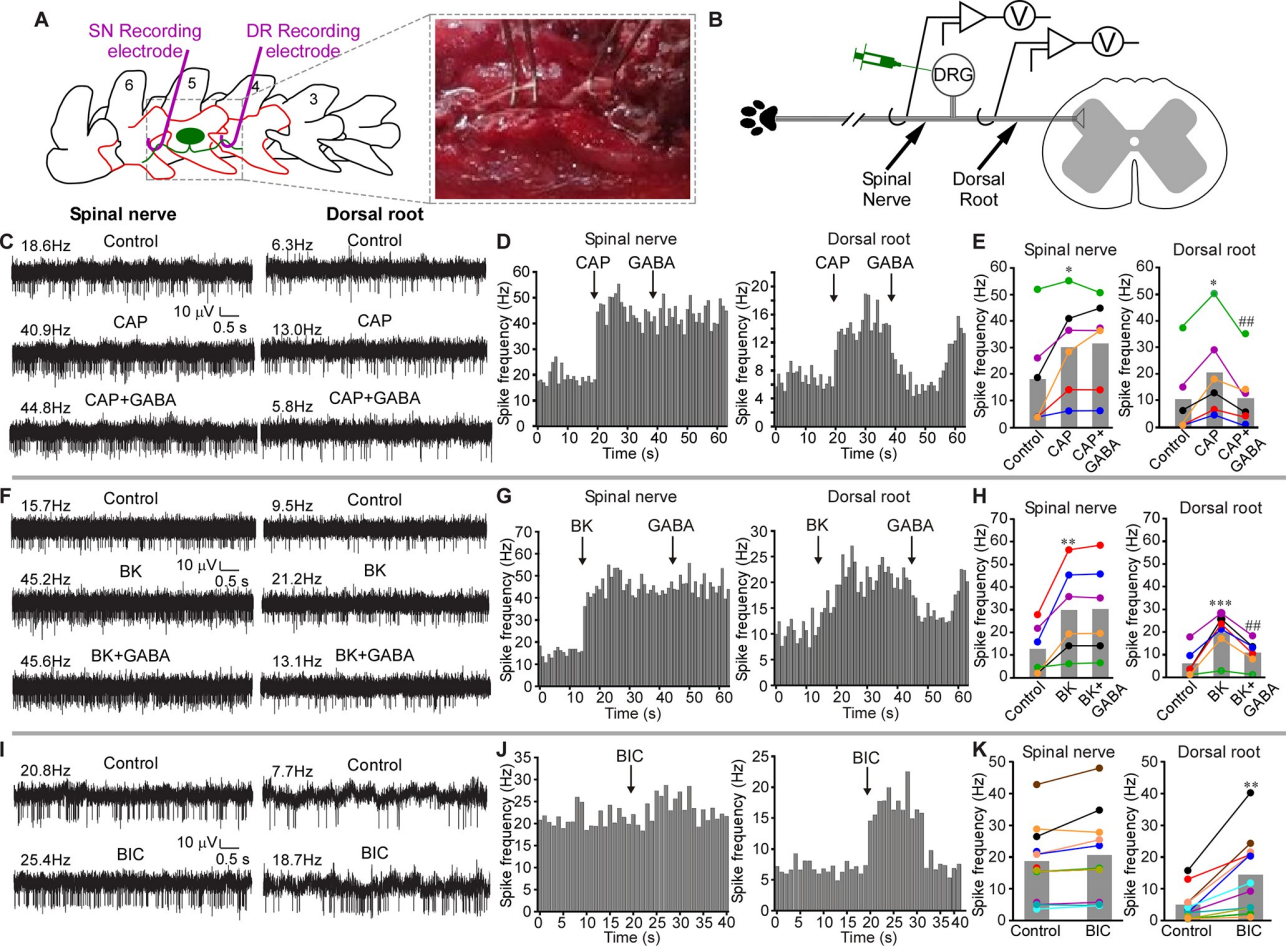
GABAergic filtering of nociceptive signals at the DRG. (**A**) Schematic of surgical exposure of the L5 SN (left), L5 DRG, and the DR (middle) in an anesthetized rat. Parts of the vertebra that are surgically removed are shown in orange. SN and DR are then individually suspended on fine hook electrodes; DRG is exposed to direct drug application. (**B**) Schematic of the electrode placement. (**C**) Hindpaw injection of Capsaicin (CAP, 10 μM, 50 μl) increased firing frequency in both SN and DR branches of the nerve (middle traces, as compared to basal activity shown in the upper traces). Application of GABA (200 μM, 3 μl) to DRG reduced CAP-induced firing frequency in DR but not SN (bottom traces). (**D**) Histograms of firing frequencies from panel C. (**E**) Summary of experiments as in panel C. Two-factor repeated measures ANOVA, with factors of nerve site (SN, DR) and drug treatment (Control, CAP, CAP+GABA) revealed main effects associated with nerve site [F(1,10) = 15.1; *p* < 0.05] and drug application [F(2,9) = 12.8; *p* < 0.05], and there was significant interaction between these factors [F(2,9) = 7.9; *p* < 0.05]. Bonferroni post hoc test: *significant difference from control (*p* < 0.05); ^##^significant difference from CAP (*p* < 0.01). (**F–H**) Similar to C–E, but Bradykinin (BK, 100 μM, 50 μl) was hindpaw injected, instead of CAP. (**H**) Two-factor repeated measures ANOVA: main effect associated with drug application [F(2,9) = 12.0; *p* < 0.05]; significant interaction between nerve site and drug application [F(2,9) = 11.5; *p* < 0.05]. Bonferroni post hoc test: **,***significant difference from control (*p* < 0.01, *p* < 0.001); ^##^significant difference from BK (*p* < 0.01). (**I–K**) GABA_A_ antagonist bicuculline (BIC, 200 μM, 3 μl) was applied to DRG instead of GABA; hindpaw was not stimulated. (**K**) Two-factor repeated measures ANOVA: main effects associated with nerve site [F(1,20) = 7.7; *p* < 0.05], drug application [F(1,20) = 12.0; *p* < 0.01], significant interaction between nerve site and drug application [F(1,20) = 18.7; *p* < 0.01]. Bonferroni post hoc test: **significant difference from control (*p* < 0.01). Metadata for quantifications presented in this figure can be found at https://archive.researchdata.leeds.ac.uk/1042/. Schematics are drawn with Canvas X 2019. DR, dorsal root; DRG, dorsal root ganglion; SN, spinal nerve.

We first developed a method for in vivo electrophysiological recording of extracellular spiking activity from both the peripheral and central branches of the L5 spinal nerve of a rat. SN, DRG, and DR were surgically exposed in anesthetized rat (Fig 1A and 1B). SN and DR were then individually suspended on fine hook electrodes, while the DRG was exposed to direct drug application. This preparation allows (i) synchronous measurement of the firing rates in the SN (before spikes enter the DRG) and DR (after spikes passed through the DRG); (ii) sensory stimulation of the hind paw; (iii) direct application of compounds or light to the DRG.

In our recordings, both the SN and DR usually displayed spontaneous firing activity (Fig 1C–1K), consistent with earlier reports [20,21]. Intraplantar injection of algogenic compounds, capsaicin (a TRPV1 agonist; CAP, 10 μM, 50 μl; Fig 1C–1E) or bradykinin (BK, 100 μM, 50 μl; Fig 1F–1H), significantly increased firing frequency in both SN and DR branches of the nerve, consistent with the evoked nociceptive inputs being transmitted from the peripheral nerve towards the spinal cord. Capsaicin injection increased firing rates to 168% and 200% of basal values in SN and DR nerves, respectively; BK injection increased firing rates in SN and DR to 241% and 326%, respectively.

Recent studies suggest there is a GABAergic inhibition at the DRG, in addition to the well-accepted spinal GABAergic inhibitory network [15,22] and that DRG neurons can themselves produce and release GABA [15,23]. We thus tested how exogenous application of GABA to the DRG would affect the propagation of peripherally induced nociceptive signals through the ganglion. Direct application of GABA (200 μM, 3 μl) to the DRG (see Materials and methods) significantly reduced capsaicin- or BK-induced firing rates specifically in the DR, having no effect on the firing rates in the SN (Fig 1C–1H). Thus, GABAergic inhibition at the DRG can induce a prominent filtering of the throughput conduction.

Interestingly, application of the GABA_A_ receptor antagonist, bicuculline (BIC; 200 μM, 3 μl) to the DRG during continuous recording of spontaneous activity in both SN and DR (in the absence of any peripheral stimulation) significantly increased firing rate in the DR but not in the SN (Fig 1I–1K). This finding is consistent with our previous observation that application of BIC via the L5-DRG-implanted cannula in vivo induces nocifensive behavior towards the hind paw [15]. Thus, BIC is likely to attenuate tonic filtering in nociceptive fibers passing through the DRG.

The interpretation of experiments presented in Fig 1 could be complicated by the presence of the ventral root (VR). Even though the experiments were conducted on immobile animals under deep anesthesia, the presence of intact motor fibers in the SN allows for execution of the flexor reflex that could contribute to SN activity via the efferent and re-afferent discharge. To account for such an eventuality, we evaluated the effect of VR transection on the spontaneous and evoked activity in SN and DR (S1A–S1J Fig; panel J schematizes the approach). VR transection had no noticeable effect on either the SN or DR spontaneous activity (S1A and S1B Fig). Capsaicin, GABA (S1C and S1D Fig), and BIC (S1E and S1F Fig) produced effects qualitatively identical to these presented in Fig 1: capsaicin increased firing rate in both SN and DR and GABA reduced this induced firing rate in DR but not SN. BIC increased firing rate in DR but not in SN. Interestingly, when GABA was applied to DRG on its own, without noxious stimulation of the paw, it failed to produce a significant effect on firing rate in both the SN and DR (S1G and S1H Fig). Another important observation from the experiments presented in Figs 1 and S1 was that firing rates in the SN were consistently higher than in the DR. Importantly, this was also true for preparations with VR transection (summarized in S1I Fig). Together with the fact that BIC consistently increased firing rates in the DR (with or without VR transection), these findings support the hypothesis for tonic filtering at DRG.

While VR transection eliminated the efferent input, it did not eliminate efferent fibers themselves from the spinal nerve; thus, any spurious activity in those fibers could have contributed to the SN activity and may have contributed to higher firing rates in the SN, as compared to DR. In order to eliminate efferent fibers, we took advantage of the fact that VR injury causes progressive degeneration of the motoneurons and preganglionic parasympathetic neurons (PPNs) [24]. Thus, we performed VR transections and allowed animals to recover; 2 weeks after VR transections, the recordings similar to these shown in Figs 1 and S1 were repeated. The motor and PPN fiber degeneration was confirmed by almost complete loss of their marker, choline acetyltransferase (ChAT) [24] at 2 weeks after VR transection (S2A Fig). Despite the removal of efferent fibers, spontaneous firing rate in the DR was still significantly lower, as compared to SN; GABA still significantly reduced the capsaicin-induced firing rate in DR but not SN, while BIC increased firing rate in DR but not in SN (S2B–S2E Fig). Thus, under our experimental conditions, motor neuron input had no significant contribution to either spontaneous or evoked activity in SN or DR.

When we tested the effects of GABA on the capsaicin-induced firing and of BIC on spontaneous activity in SN and DR in female rats (S3 Fig) and on male rats anesthetized with different anesthetic (isoflurane instead of pentobarbital i.p.; S4 Fig), we obtained results qualitatively identical to these shown in Figs 1, S1 and S2. Thus, GABA-mediated modulation of ganglionic filtering is a phenomenon reliably observed under a variety of experimental conditions in animals of either sex.

**Fig 2:**
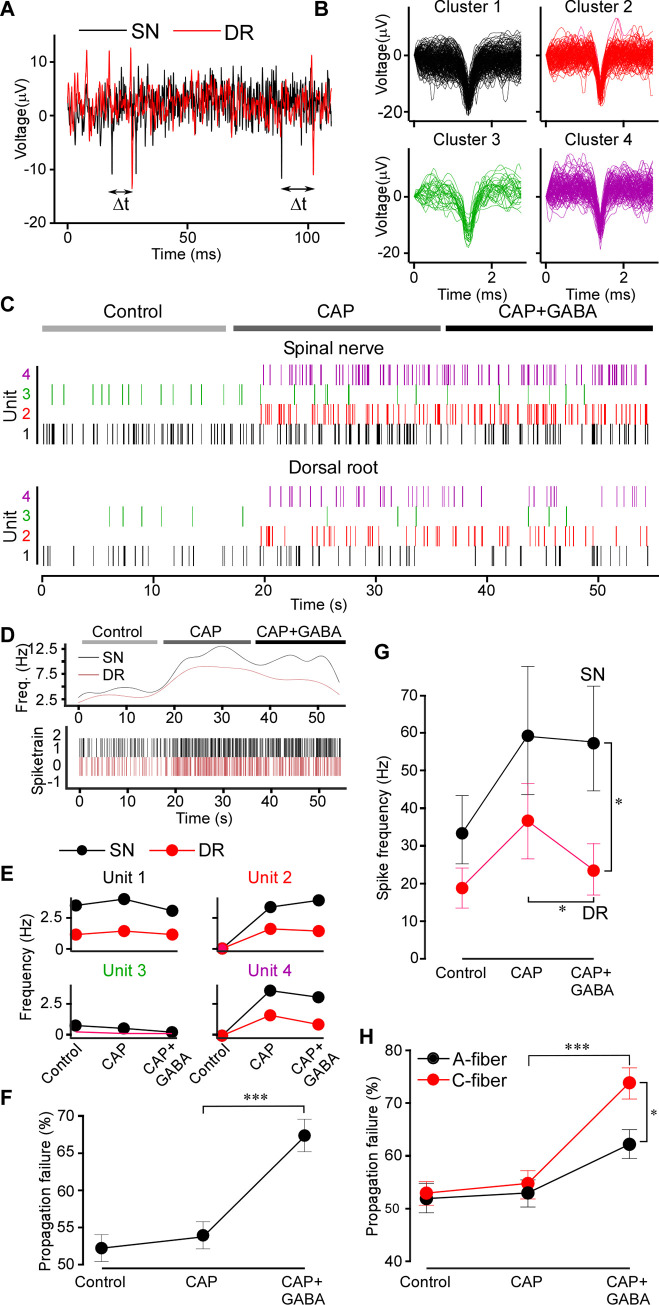
Spike analysis confirms GABAergic filtering. (**A**) Latencies between the SN and DR spikes were measured; the minimum latency defined the SN origin of a DR spike. (**B**) Extracellular spike waveforms extracted from a spinal nerve recording and clustered using WaveClus. (**C**) Raster plot for each clustered waveform (denoted as Unit) under control, CAP and CAP+GABA conditions after matching the DR spikes with these in the SN. Note: capsaicin induced firing of specific units. (**D**) Instantaneous firing frequency of the all identified units shown in C in the DR and SN. (**E**) Firing rates of individual units identifies capsaicin-sensitive units. (**F**) Propagation failure (%) of all matched spike units under control, CAP and CAP+GABA conditions. One-way ANOVA: F(2,117) = 17.7; *p* < 0.001; Tukey post hoc test: ***significant difference from CAP, *p* < 0.001. (**G**) Average firing rates for all multi-unit nerve recordings in the dataset (*n* = 6) in SN and DR under control, CAP and CAP+GABA conditions. Two-factor (nerve site, drug application) repeated measures ANOVA: main effects associated with drug application [F(2,10) = 6.38; *p* < 0.05], significant interaction between factors [F(2,10) = 10.74; *p* < 0.01]. Bonferroni post hoc test: *significant difference from control (*p* < 0.05). (**H**) Units were divided into “C-type” and “A-type” based on the SN-DR latency (C: <1.2 m/s; A: >1.2 m/s) and propagation failure rate analyzed as in (F). Two-factor (fiber type, drug application) mixed-effects ANOVA: main effect associated with drug application [F(2,70) = 34.82, *p* < 0.001], significant interaction between factors [F(2,72) = 4.712, *p* < 0.05]. Sidak post hoc test: *significant difference between CAP+GABA and CAP, significant difference between A and C fibers (*p* < 0.05). Metadata for quantifications presented in this figure can be found at https://archive.researchdata.leeds.ac.uk/1042/. Code for spike sorting analysis is available at GitHub (https://github.com/pnm4sfix/SpikePropagation). DR, dorsal root; SN, spinal nerve.

In order to better understand filtering of specific spikes in the recordings as these exemplified in Fig 1, we developed a spike-matching method allowing to correlate SN and DR spikes (Figs 2A and S5–S7; see Spike sorting section of Materials and methods for detailed description of the approach). We used this method to analyze sample datasets from recordings shown in Fig 1. This method proved accurate (correct matching of 80 to 100% spike pairs) in computer-generated, Poisson spike trains up to 100 Hz irrespective of the degree of filtering; the accuracy was inversely proportional to firing frequency (S5A and S5B Fig). The rate of false positive matching (FPR) was relatively low across fiber types (mostly below 7%), but faster conducting fiber types were more likely to have a higher FPR at higher spike train frequencies (S5C Fig).

Sorting extracellular SN spike waveforms using the WaveClus implementation of super-parametric clustering allowed us to isolate distinct spike clusters (units) and match these between SN and DR recordings. A representative experiment capturing the firing activity of 4 distinct units is shown in Fig 2B–2E. Another example of a similar dataset with 10 units analyzed is shown in S6 and S7 Figs. Capsaicin-responsive units were clearly identifiable, noted by the onset of activity during application of capsaicin (Fig 2C and 2E). CAP-evoked spikes were characterized by a larger amplitude on average (CAP-insensitive units: 14.5 ± 1.5 μV; CAP-responsive units: 19.6 ± 0.9 μV; *p* < 0.01). However, capsaicin-responsive units could not be well distinguished by latency and spike width in this way.

Individual spike waveforms in sorted units (cluster) in the SN and corresponding average waveforms are shown in S6B and S6C Fig, correspondingly. Average waveforms of matched units in the DR are shown in the S6D Fig. To rule out contamination of DR units with synchronized firing of another fiber, we calculated the mean deviation (represented as a z score) of each waveform in the unit from the mean waveform of the unit (S7A Fig). Any spikes originating from another fiber firing in a temporally correlated way should exhibit a different waveform shape and thus be recognized as an outlier (>3 z score). The large majority of spikes were within a z score of 3 from the unit means, suggesting our matching protocol identified homogenous and distinct DR firing units. Mean latencies for each of the 10 unit analyzed are shown in S7B Fig.

The mean difference in spiking units between SN and DR was significantly greater with GABA administration after CAP (Fig 2G and 2H), revealing disappearance (failure) of spikes in the DR specifically upon application of GABA. We further characterized spike units as either A-fiber or C-type based on conduction velocity (<1.2 m/s; presumably C-type; >1.2 m/s, presumably A-fibers [25]). Interestingly, this revealed that C-type fibers were indeed significantly more filtered, as compared to A-type fibers, during application of GABA (Fig 2H).

Taken together, data presented in Figs 1 and 2 and S1–S7 provide the following observations: (i) there is a basal activity in the SN and DR even in the absence of peripheral stimulation; (ii) the firing rates in the SN are higher than these in DR, suggesting presence of “tonic filtering” of this basal activity at the DRG. (iii) DRG-applied GABA_A_ antagonist (BIC) increased basal firing in the DR but not in the SN. (iv) Noxious stimulation increases firing rates in both SN and DR, but the DRG-applied GABA reduced firing in the DR specifically, thus enhancing GABAergic filtering in the nociceptive fibers. Therefore, the filtering at the DRG can also be dynamic, that is, it can be readily increased or decreased by modulating mechanisms. (v) Exogenous GABA did not affect the basal firing rate (either in the SN or DR). This may indicate that some fibers may be already inhibited by GABA tone at the basal conditions and adding extra GABA does not affect these but inhibiting GABA channels may relieve inhibition of these “sensitive” fibers. Generally, after BIC the firing rates in the DR were still somewhat lower than in the SN; hence, not all tonic filtering is necessarily GABAergic.

### Transplantation of forebrain GABAergic neuron precursors into the adult mouse DRG in vivo delivers an analgesic mechanism

**Fig 3:**
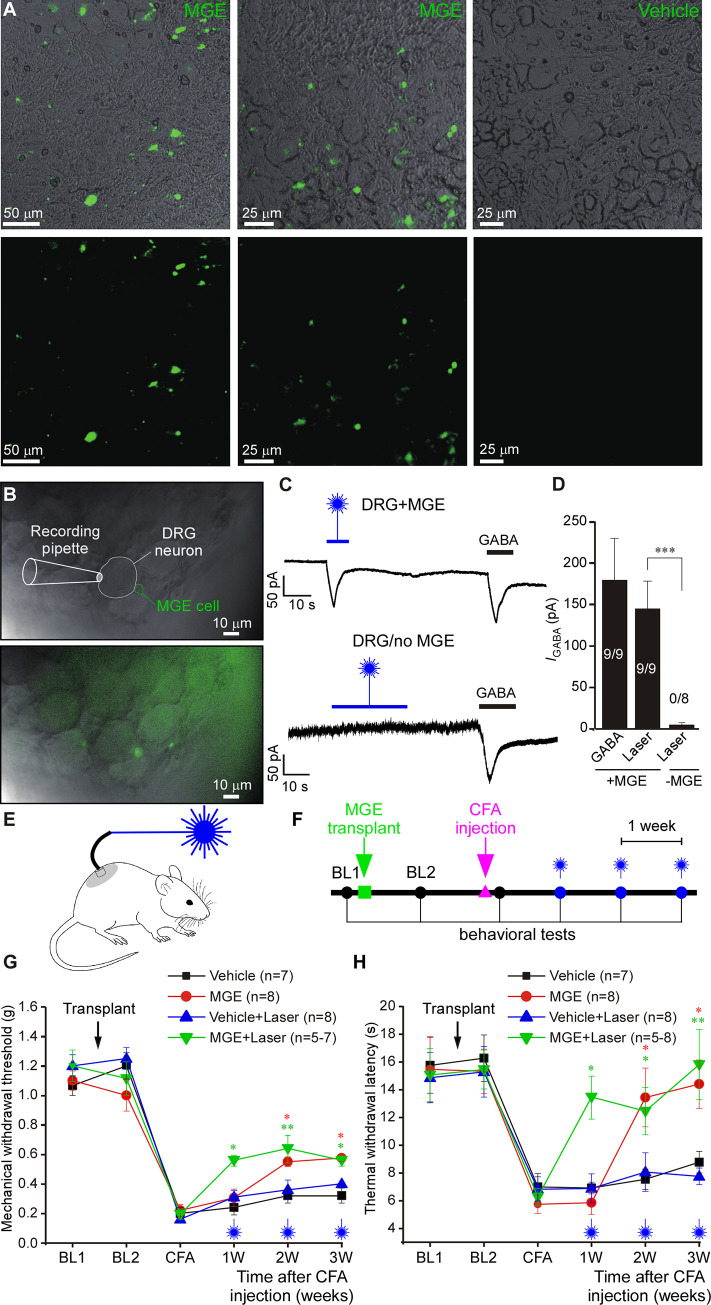
Transplantation of forebrain GABAergic neuron progenitor cells into the adult mouse DRG in vivo delivers an analgesic mechanism. (**A**) Fluorescence micrographs of DRG sections of mice 4 weeks after the injection with a suspension of MGE cells derived from VGAT-ChR2-eYFP mice. Control mice (right images) received vehicle injections. (**B**) Bright-field and overlaid fluorescence images of “loosened” DRG preparation used for patch clamp recording. The whole L4 DRG transplanted with VGAT-ChR2-eYFP MGE cells was extracted 4 weeks after transplantation. Recording was made from a DRG neuron juxtaposed to fluorescent MGE cell (top image) using whole-cell voltage clamp. (**C**) Top: example trace of continuous current recording (−60 mV) from the cell shown in B; stimulation with 473 nm blue laser (3 mV) induced inward current, similar in amplitude and kinetics to the current induced by perfusion of GABA (200 μM). Bottom: similar recording from a DRG of a vehicle-injected mice. (**D**) Summary for panel C; one-way ANOVA: F(2,23) = 5.9; *p* < 0.01; Bonferroni post hoc test: ***significant difference between +MGE vs. -MGE (*p* < 0.001). Number of recorded/responsive cells is indicated within each bar. (**E**) Schematic of the in vivo optogenetic DRG stimulation. (**F**) Timeline of the in vivo behavioral testing after the MGE cell transplantation and hindpaw injection of CFA. (**G, H**) Hypersensitivity to mechanical (**G**) and thermal (**H**) stimulation caused by hindpaw injection of CFA 2 weeks after MGE cells transplantation into L4 DRG of mice. At a time of MGE transplantation, mice were also implanted with the fiberoptic light guide. Mechanical and thermal sensitivity was measured using the von Frey and Hargreaves methods, respectively. Starting at 1 week after the CFA injection, measurements were performed while stimulating the L4 DRG with 473 nm laser. Black and blue symbols denote control mice DRG-injected with vehicle without and with optogenetic stimulation, respectively. Red and green symbols denote MGE-transplanted mice without and with optogenetic stimulation, respectively. BL1: baseline before transplantation; BL2: baseline after transplantation; CFA: 1 day after the plantar injection of CFA. (**G**) Three-factor (MGE vs. vehicle, time after CFA, laser stimulation) ANOVA: main effects associated with MGE transplantation [F(1,24) = 50.9; *p* < 0.001], time after CFA [F(2,23) = 6.6; *p* < 0.01]; laser stimulation [F(1,24) = 8.9; *p* < 0.01]. Bonferroni post hoc test: red* indicate the difference between MGE group and vehicle group within the corresponding time point; green* indicate the difference between MGE with laser stimulation group and vehicle with laser stimulation group; *p < 0.05, **p < 0.01, ***p < 0.001. (**H**) Three-factor (MGE vs. vehicle, time after CFA, laser stimulation) repeated measures ANOVA: main effects associated with MGE transplantation [F(1,24) = 37.4; *p* < 0.001], time after CFA [F(2,23) = 6.1; *p* < 0.01], laser stimulation [F(1,24) = 2.4; *p* = 0.12]; significant interaction between time and laser stimulation [F(2,23) = 2.5; *p* = 0.09] and between MGE and laser stimulation [F(2,23) = 3.2; *p* = 0.08]. Bonferroni post hoc test: red* indicate the difference between MGE group and vehicle group within the corresponding time point; green* indicate the difference between MGE with laser stimulation group and vehicle with laser stimulation group; *p < 0.05, **p < 0.01, ***p < 0.001. Metadata for quantifications presented in this figure can be found at https://archive.researchdata.leeds.ac.uk/1042/. Schematics are drawn with Canvas X 2019; vector diagram of laboratory mouse from Wikimedia was used in the panel E, https://commons.wikimedia.org/wiki/File:Vector_diagram_of_laboratory_mouse_(black_and_white).svg. CFA, complete Freund’s adjuvant; DRG, dorsal root ganglion; MGE, medial ganglionic eminence.

To test how GABAergic filtering of nociceptive transmission at the DRG can be exploited in vivo, we adopted an approach developed by Basbaum’s group, who were able to transplant and functionally integrate into dorsal spinal cord, embryonic GABAergic progenitor cells from the medial ganglionic eminence (MGE). Transplanted MGE cells were able to compensate for the loss of spinal GABAergic inhibitory system observed in neuropathic pain models [26,27]. We transplanted embryonic MGE cells derived from VGAT-ChR2-eYFP into the L4 DRG of WT C57 mice. L4 DRG was chosen in this case as it is the major contributor to the sciatic nerve in mice [28]. At 4 weeks after the DRG injection, we observed numerous YFP-positive cells in the DRG sections (Fig 3A); fluorescent cells were entirely absent in vehicle-injected control animals.

In order to confirm that transplanted MGE cells can function as GABAergic neurons within DRG, we performed patch clamp recordings from the DRG neurons juxtaposed to the MGE cells (Fig 3B–3D) using “loosened” whole L4 DRGs from mice pre-injected (4 weeks) with the VGAT-ChR2-eYFP-expressing MGE (see Materials and methods). Stimulation of the ganglion with the 473 nm blue light induced inward currents in 9/9 DRG neurons, which were in close juxtaposition with MGE cells (Fig 3B–3D). These same neurons also responded to perfusion of 200 μM GABA with very similar inward currents. In contrast, DRG neurons from vehicle-injected mice never responded to blue light (0/8 neurons) but these did respond to GABA (Fig 3C and 3D). These results suggest that (i) implanted MGE progenitor cells can survive and maturate to produce GABA-releasing neurons in DRG; and (ii) stimulus-induced release of GABA by resident neurons can induce a response in neighboring neurons.

Next, we tested if optogenetic release of GABA from the implanted MGE cells can alleviate hypersensitivity to noxious stimuli in chronic pain models. In these experiments, a fiber-optic light guide was implanted into the DRG immediately after the MGE cells transplantation (Fig 3E; Materials and methods). Chronic inflammation with hind paw injection of complete Freund’s adjuvant (CFA, 20 μl) induced significant hypersensitivity to mechanical and thermal stimuli (Fig 3F–3H). We then performed mechanical (Fig 3G) or thermal (Fig 3H) sensitivity tests while stimulating ipsilateral L4 DRG with blue light. Optical stimulation significantly reduced both types of hypersensitivity in MGE-injected mice. Interestingly, starting from the second week after the CFA injection, both mechanical and thermal hypersensitivity in the MGE-implanted mice started to recover even in the absence of optogenetic stimulation and by the third week after the CFA injection blue light stimulation no longer produced any additional analgesic effect (Fig 3G and 3H). We hypothesized that a buildup of tonic GABA release from the transplanted MGE cells in DRG could be responsible for the light-stimulation-independent recovery of the CFA-induced hypersensitivity. This hypothesis was corroborated in experiments, similar to the ones presented in Fig 3G and 3H, but in which no optogenetic stimulation was used, to avoid inducing any stimulus-induced GABA release (S8A and S8B Fig). We also utilized a chronic constriction injury (CCI) model of neuropathic pain in similar experiments (S8C and S8D Fig). In both models, hypersensitivity developed in control (vehicle-injected) and MGE-implanted mice. However, the latter group displayed significantly quicker and more complete recovery. Collectively, these data suggest that DRG-implanted MGE cells can be stimulated to release GABA locally in vivo and that such release produces analgesic effect.

### Optogenetic release or direct application of GABA to the DRG enhances filtering of spikes triggered by noxious but not innocuous stimuli

**Fig 4:**
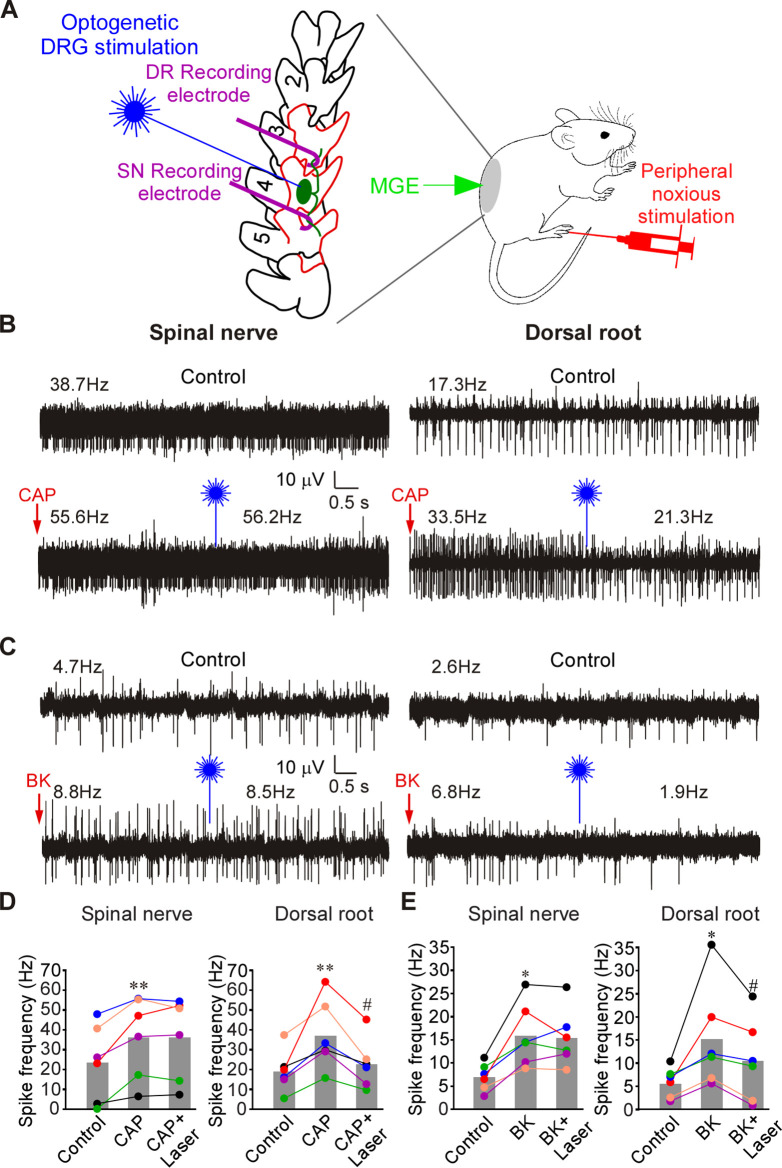
Optogenetic activation of MGE cells in DRG reduces frequency of firing induced by CAP or BK in the DR but not in the SN. (**A**) Schematic of the recording paradigm: mice were DRG-transplanted with VGAT-ChR2-eYFP MGE cells 3–4 weeks before recordings; recordings were done as shown in Fig 1B (but from L4 DRG), supplemented with optical stimulation of the exposed DRG. (**B**) Hindpaw injection of CAP (10 μM, 20 μl; indicated by red arrow) increased firing frequency in both SN and DR. Application of 473 nm laser light to DRG (indicated by blue symbol) acutely reduced CAP-induced firing frequency in DR but not SN (bottom traces). (**C**) Experiment similar to A, but BK (100 μM, 20 μl) was hindpaw injected, instead of CAP. (**D**) Summary of panel B. Two-factor (nerve site, drug application) repeated measures ANOVA: main effect associated with drug treatment [F(2,9) = 11.4; *p* < 0.05] and significant interaction between nerve site and treatment [F(2,9) = 7.6; *p* < 0.05]. Bonferroni post hoc test: **significant difference from control (*p* < 0.01); ^#^significant difference from CAP (*p* < 0.05). (**E**) Summary of panel C. Two-factor (nerve site, drug application) repeated measures ANOVA: main effect associated with nerve site [F(1,10) = 6.7; *p* < 0.05] and significant interaction between nerve site and treatment [F(2,9) = 9.5; *p* < 0.05]. Bonferroni post hoc test: *significant difference from control (*p* < 0.05); ^#^significant difference from BK (*p* < 0.05). Metadata for quantifications presented in this figure can be found at https://archive.researchdata.leeds.ac.uk/1042/. Schematics are drawn with Canvas X 2019; vector diagram of laboratory mouse from Wikimedia was used in the panel E, https://commons.wikimedia.org/wiki/File:Vector_diagram_of_laboratory_mouse_(black_and_white).svg. DR, dorsal root; DRG, dorsal root ganglion; MGE, medial ganglionic eminence; SN, spinal nerve.

We hypothesized that the analgesic effect of MGE cells transplanted into the DRG is mediated by GABAergic filtering of pro-nociceptive spikes at the DRG. To test this, we used an approach similar to that used in Figs 1 and S1–S7, but instead of applying GABA, we stimulated L4 DRG with blue laser light (Fig 4A). Optogenetic DRG stimulation (3 to 4 weeks after MGE transplantation) gave rise to qualitatively very similar effects to those produced by application of GABA. Firing induced by the hind paw injections of capsaicin (10 μM, 20 μl, Fig 4B and 4D) or BK (100 μM, 20 μl, Fig 4C and 4E) was significantly inhibited by the light stimulation in DR but not SN. The optogenetic suppression of firing in DR was evident immediately upon application of blue light (Fig 4B and 4C; lower right panels).

Application of noxious heat (60°C water; S9A and S9B Fig) and noxious cold (ice; S9C and S9D Fig) induced a significant increase of firing frequencies in both SN and DR and optogenetic DRG stimulation significantly inhibited firing rates in DR but not in SN. We then tested innocuous and noxious mechanical stimulation. Air puffs and subthreshold (4 g) von Frey filament stimulation (S9E–S9H Fig) increased firing in both SN and DR, presumably via the activation of low threshold mechanoreceptors in the skin [29,30]. Interestingly, blue light illumination of DRGs did not significantly affect the firing frequency in either SN or DR in these experiments (S9E–S9H Fig). Noxious mechanical stimulation of the paw with a blunt glass needle also significantly increased firing frequencies in both SN and DR. In this case, optogenetic stimulation substantially inhibited firing in DR, but not in SN (S9I and S9J Fig). Thus, it appears that GABAergic filtering in DRG predominantly exists in nociceptive fibers.

Next, we performed a set of experiments, similar to that shown in S9 Fig but in naïve rats and with the direct injection of GABA into the DRG instead of optogenetic stimulation (S10 Fig). There was a pattern similar to that observed with optogenetic stimulation of MGE cells in mice. Firing induced by noxious heat (S10A and S10B Fig) and noxious cold (S10C and S10D Fig) was selectively inhibited in the DR but not in the SN by the DRG-applied GABA. No significant effects of DRG-applied GABA were seen when firing was induced by innocuous air puffs or subthreshold von Frey hairs (S10E–S10H Fig). In contrast, firing induced by the noxious needle prick was reduced in the DR but not in the SN (S10I and S10J Fig). Striking similarity of the effects of exogenous GABA and optogenetic GABA release in DRG strongly suggest that (i) the GABAergic system modulates filtering efficacy of the ganglia; (ii) the filtering is most efficient in nociceptive fibers and (iii) such filtering is the most plausible explanation of the analgesic effect of MGE cells transplanted to the DRG; (iv) GABAergic filtering exists in both rats and mice.

**Fig 5:**
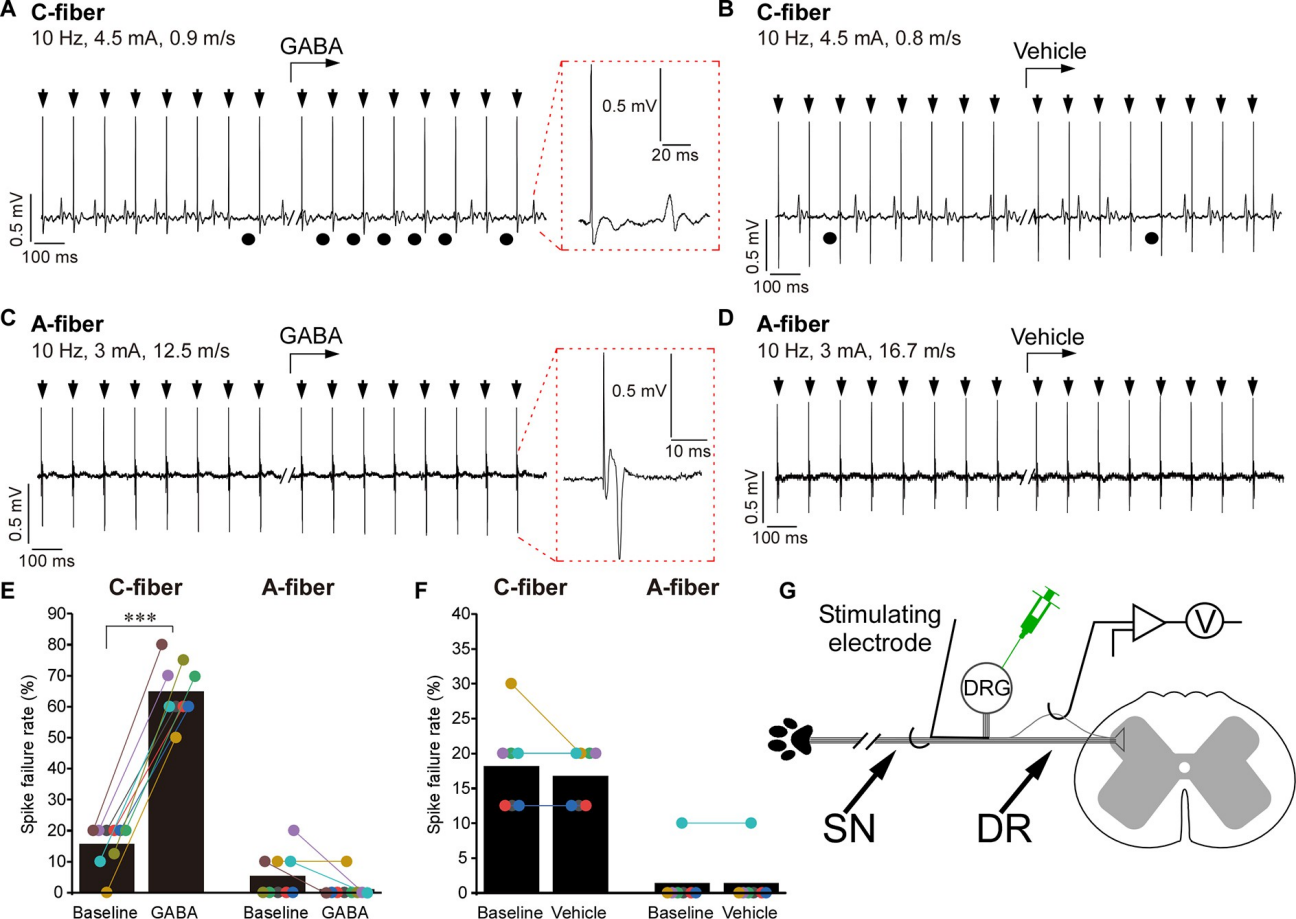
Single-unit recordings: DRG application of GABA induces spike failure at the DRG, which is specific to C fibers. (**A–D**) Example traces of in vivo single-unit recording from the DR process of a rat C fiber (A, B) or A fiber (C, D); stimulus electrode is placed in the SN (schematic of the experiment is shown in panel D). Parameters of stimulation and conduction velocity are indicated above the trace. GABA (200 μM, 3 μl; A, C) or vehicle (3 μl saline; B, D) were applied to the DRG by a micropipettor at the time point indicated by the bent arrows. Stimulus artifacts are indicated by black arrows. Black circles under the traces indicate failed spikes. Spike waveform is shown on the extended time scale within the dotted red boxes. (**E**) Spike failure rate before and during application of GABA is analyzed for C and A fibers; *n* = 9 (1 recording per animal) for both fiber types; Kruskal–Wallis ANOVA: H(3) = 28.2, *p* < 0.001; Mann–Whitney post hoc test: ***significant difference from control (*p* < 0.001). (**F**) Similar to panel E but spike failure rates before and after the application of vehicle control are analyzed; *n* = 7 for both fiber types. No significant effects of the vehicle were found (Kruskal–Wallis ANOVA). (**G**) Schematic of the single-unit recording paradigm. Metadata for quantifications presented in this figure can be found at https://archive.researchdata.leeds.ac.uk/1042/. Schematics are drawn with Canvas X 2019. DR, dorsal root; DRG, dorsal root ganglion; SN, spinal nerve.

To test this further, we performed single-unit recordings (Fig 5A–5G, schematized in panel G). Solutions (200 μM GABA, 1 μM TTX, or saline; all in 3 μl volume) were applied to the DRG by micropipettor and firing was induced before or after the injection by a stimulus train delivered by a stimulating electrode placed in SN while recordings were made from the mechanically isolated teased DR bundles. A-type and C-type spikes were distinguished by the conduction velocity (A fibers >1.2 m/s; C fibers <1.2 m/s [25]). The firing in both fiber types was blocked by 1 μM TTX applied to the DRG (S11A, S11B and S11E Fig). In single-unit recordings evoked stimuli propagated reliably in both fiber types under control conditions with only occasional failures (basal failure rate in A fibers: 5.6 ± 2.4%, *n* = 8; C fibers: 15.8 ± 2.4%, *n* = 9; Fig 5A–5F), which is consistent with earlier report [18]. At 10 Hz stimulation, DRG-applied GABA significantly increased failure rate in C fibers (to 65.0 ± 3.1%, *p* < 0.001) but had no significant effect in A fibers (Fig 5A and 5C and 5E). Even when stimulation frequency was increased to 50 and 100 Hz, DRG applied GABA still failed to affect spike propagation in A fibers (S11C and S11D and S11F Fig). DRG injection of vehicle (saline) had no effect on filtering (Fig 5B and 5D and 5F). Due to technical difficulties of these recordings not all of these were long enough to reliably analyze failure rate but among all the recordings made, 90% (38/42) of C fibers and only 17% (11/63) of A-type fibers displayed GABA-induced t-junctional spike failure, while in 100% of both fiber types failure was induced by TTX (S11E Fig). Taken together, single-unit recordings revealed that DRG application of GABA selectively increases filtering of C-fiber activity.

### Does the soma have more influence over the t-junction in C-type as compared with A-type fibers?

**Fig 6:**
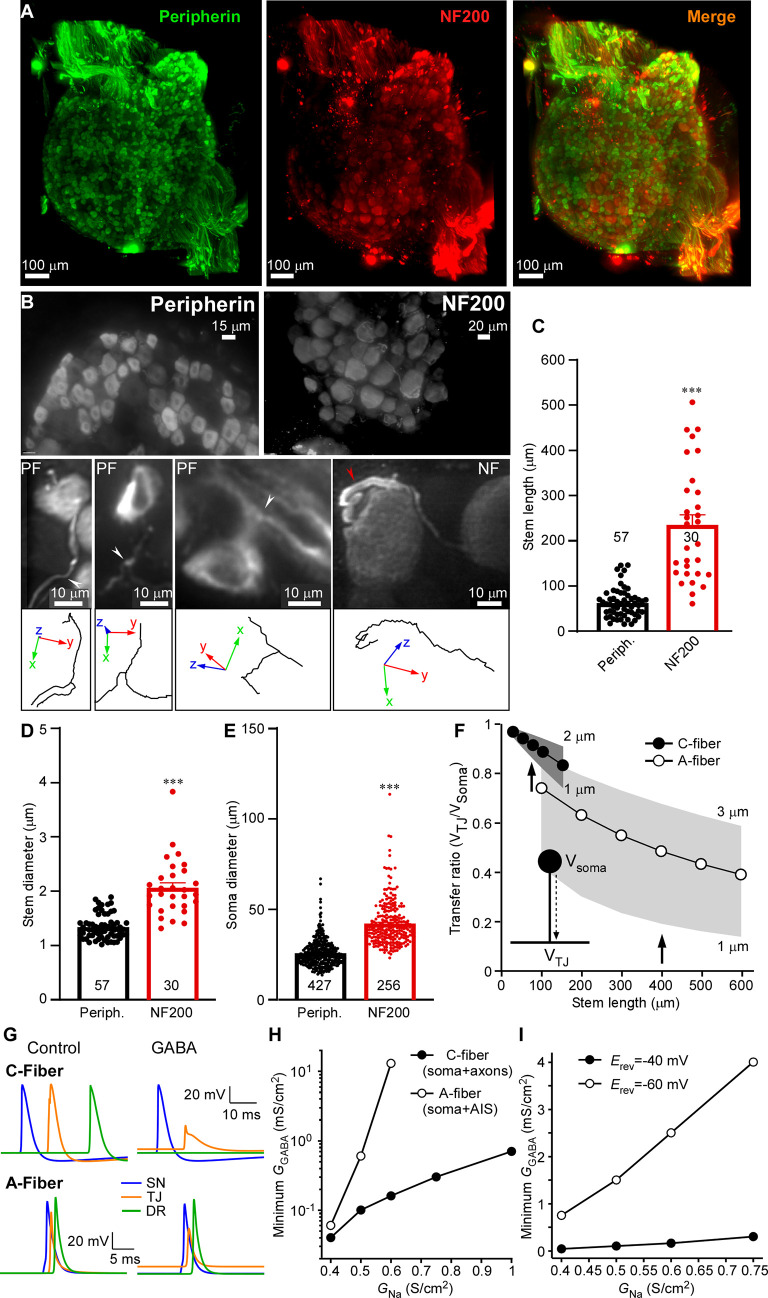
Stem axon morphology defines the efficiency of filtering in DRG. (**A, B**) Light-sheet microscopy of cleared rat DRG (see also S1 Movie). C-fibers are labeled with peripherin and A-fibers are labeled with NF-200. Stem axons were traced until bifurcation point using simple neurite tracer in FIJI (S2 Movie). White arrowheads indicate t-junctions, red arrowhead point at glomerular region of A-fiber stem axon that increases the length. (**C–E**) Quantification of the stem axon lengths (C), stem diameter (D), and somatic diameter (E) of the peripherin- and NF-200-labeled fibers is shown. Data are from 10 lumbar DRGs (peripherin) and 7 lumbar DRGs (NF-200) from 3 rats; *n* number is given within each bar. Unpaired *t* test: t(85) = 9.8, *p* < 0.001 (C); t(85) = 9.8, *p* < 0.001 (D); t(681) = 21.6, *p* < 0.001 (E). Per-animal analysis of data in C–F are shown in S12 Fig. (**F–I**) Biophysical modeling of sensory neurons. (**F**) Voltage attenuation from the soma to the t-junction (TJ) measured as the transfer ratio (V_TJ_/V_Soma_) for both C- and A-fiber models, across a physiological range of stem axon lengths and diameters. Note that there is overlap only at the shortest range of A-fiber model lengths and widest diameters. Thus, voltage attenuation is predicted to be greater for A-fiber than C-fiber stem axons. (**G**) Top traces are from a C-fiber model that exhibits spike propagation in the absence of GABA_A_ receptor activation and GABA-sensitive spike failure. In this case, G_Na_ across the model was lowered (0.4 S/cm^2^) and GABA receptors (0.04 mS/cm^2^) expressed in the soma and axons. In contrast, traces from an A-fiber model configured to be insensitive to the effects of GABA; higher G_Na_ (0.75 S/cm^2^) and GABA expression (0.02 mS/cm^2^) restricted to the soma and AIS. (**H**) Across a wide range of G_Na_ densities, the C-fiber model was more GABA-sensitive to spike failure compared with the A-fiber model. In contrast, the A-fiber model with GABA receptors restricted to the soma and AIS is less sensitive. Except for the lowest density of G_Na_, a higher density of GABA receptor activation is required to induce spike failure. Moreover, at higher G_Na_ densities (greater than 0.6 S/cm^2^ in the model) GABA receptor activation at any level no longer prevents spike propagation. (**I**) In a C-fiber model where E_Cl_ was set to the resting potential, and did not depolarize membrane potential, the minimum GABA conductance required to block spikes was greater than when E_Cl_ = −40 mV across a range of G_Na_, consistent with the requirement for inactivation of Na^+^ channels to lower the safety factor for spike propagation. Metadata for quantifications presented in this figure can be found at https://archive.researchdata.leeds.ac.uk/1042/. Code for modeling panels can be found at GitHub (https://github.com/dbjaffe67/DRGsims). Schematics are drawn with Canvas X 2019. AIS, axon initial segment; DRG, dorsal root ganglion.

DRG neurons are pseudo-unipolar and axonal bifurcation (t-junction) is potentially a major site of spike filtering in DRG due to impedance mismatch [13,15–17,31–33]. Why is GABA-induced filtering more efficient in the C-fibers, as compared to A-fibers? One possibility is the different length of the stem axon (from the soma to the t-junction) and, thus, the electrotonic influence of the soma on the t-junction and spike propagation: the longer the stem axon, the poorer the coupling [14,15,17]. To our knowledge, there has been no systematic analysis of stem axon lengths in mammalian DRG neurons, although drawings by Ramon y Cajal depict much shorter stems in small-diameter neurons, as compared to larger ones [34]. In addition, larger neurons are often depicted having stems with a winding “glomerular” section, extending the length [34–36]. In order to assess stem length in C-type versus A-type fibers, we cleared [37] rat whole DRG mounts and immunostained them with the C-fiber marker, peripherin, and A-fiber marker, neurofilament-200 (NF-200). We then performed light-sheet microscopy of entire ganglia (S1 Movie) and measured the stem axon lengths of peripherin-positive and NF-200 positive neurons (S2 Movie and Fig 6A). Consistently, peripherin labeled neurons with much smaller somatic diameter, as compared to NF-200 positive neurons (26.1 ± 0.4 μm versus 42.4 ± 0.8 μm, *p* < 0.001; Figs 6A and 6E and S12). Stem axon diameters of peripherin-labeled neurons were also consistently smaller (1.34 ± 0.03 μm versus 2.1 ± 0.1 μm, *p* < 0.001; Figs 6D and S12). Of note, axonal diameters for NF-200 positive neurons reported here do not include myelin and are in good agreement with previous literature [38]. Peripherin-labeled neurons displayed much shorter stems, as compared to NF-200 positive neurons (60.7 ± 4.2 μm versus 232.5 ± 22.9 μm, *p* < 0.001; Figs 6C and S12). While for all peripherin-positive neurons analyzed in Figs 6C and S12, the t-junction was reliably identified (S2 Movie), it was often impossible to confidently locate the t-junctions of NF-200 positive neurons as these were too far away from the cell body. In these instances, stem length was recorded as the longest traceable distance and, hence, it is an underestimation of the real stem length.

We hypothesized that one reason for why GABAergic control of DRG filtering is less effective in A-fibers compared to C-fibers may be due to the differences in stem axon length and the influence of somatic conductance load. Our previous computational modeling suggested that in a C-fiber with 75-μm stem axon, activation of somatic GABA_A_ channels could indeed cause a failure of action potential to propagate through the t-junction due to a combination of the impedance drop and sodium channel inactivation [15]. But even though data presented in Fig 6A–6C indicate that the stem axon of C-fibers is, on average, at least 3 times shorter than that of A-fibers, myelination could possibly compensate for the longer distance. Additionally, the larger diameter of A-fiber neurons, and greater conductance load, might also compensate for a longer stem axon. Using a computational model of an A-fiber neuron, we examined the relationship between somatic conductance and stem axon length. We constructed a minimal model of an A-type neuron (see Materials and methods) containing a limited repertoire of voltage-gated channels and a geometry consistent with the parameters obtained from the light-sheet morphometry of the NF-200 positive neurons and compared it to a model with a C-fiber morphology [15].

We first examined how potential at the soma influences the t-junction between the 2 models. We calculated the voltage transfer ratio, the fractional potential reaching the t-junction produced by a DC potential generated at the soma, for a passive model as a function of stem axon length and diameter for the 2 models (Fig 6F). In C-fibers, the transfer ratio was greater than 0.75 for a physiological range of stem lengths, while it was less than 0.6 for stem axon lengths measured from A-type neurons. For A-fibers, electrotonic control was comparable to the C-fiber model when stem axon lengths were limited to the shorter end of the range and stem axon diameter was wider. Thus, based simply on electrotonic distance, modeling predicts that the soma of a C-fiber neuron should have more influence on the t-junction than for an A-type neuron soma.

We then examined the propagation of spikes initiated in the most distal portion of the SN axon through the t-junction into the DR axon. For a myelinated A-fiber model, spikes at the nodes of Ranvier in SN and DR axons were 86 and 91 mV in amplitude, respectively (nodal G_Na_ = 0.2 S/cm^2^). Conduction velocity in the SN axon was 7 m/s for an internode distance of 150 μm. In our unmyelinated C-fiber model, spikes were 89 mV in amplitude in SN and DR axons (G_Na_ = 0.4 S/cm^2^), and SN axon conduction velocity was 0.2 m/s (Fig 6G, left). In both models, the repertoire of active conductances was limited to only TTX-sensitive Na^+^ [39] and delayed rectifier K^+^ [40] channels, allowing us to compare the effects of geometry between the 2 models. For the A-fiber model, we started with a stem axon of 400 μm and 2 μm diameter, and the C-fiber model started with a stem axon of 75 μm and diameter 1.35 μm. Conduction through the t-junction across a wide range of physiologically relevant parameter space was not influenced by firing at the soma, consistent with earlier models [41].

To assess the potential effect of GABA_A_ receptor activation on spike propagation through the DRG, a chloride conductance (G_Cl_) was introduced with E_Cl_ = −40 mV [42]. When GABA_A_ receptors were introduced only to the soma, an increase in somatic G_Cl_ depolarized the t-junction and, in turn, reduced local spike amplitude in both models. GABA_A_ receptor activation at the soma only could block spike propagation in both models, but the minimum conductance required to block propagation for the A-fiber model was 0.25 mS/cm^2^ (25 nS net conductance accounting for area) compared with 0.5 mS/cm^2^ (5 nS net conductance) for the C-fiber model (G_Na_ = 0.5 S/cm^2^). Currently, there is no evidence that GABA_A_ receptors are limited to the soma, and it is possible that GABA_A_ receptors are expressed on both the soma and axons of sensory neurons, although the total conductance provided is likely to be less at nodes of Ranvier. The greatest sensitivity to GABA_A_ receptor activation for the C-fiber model was best achieved when GABA_A_ receptors were expressed throughout the model (Fig 6G, right). In this case, the minimum G_Cl_ needed to block spikes (i.e., threshold) was substantially lower in the C-fiber model, over a wide range of Na^+^ channel densities (Fig 6H). As expected, for A-fiber models when the stem axon length was systematically varied, we found that longer stem axons had less influence on t-junction potential, and in turn, Na^+^ channel inactivation (S13 Fig).

We expect an increase in G_Cl_ to not only depolarize the t-junction, but also to act as a shunt via the increased membrane conductance, which would be expected to affect local impedance. In order to separate the effects of shunting versus depolarization, in the C-fiber model we compared the minimum G_Cl_ needed to block spikes at 2 equilibrium potentials for chloride (E_Cl_); first the model was set to realistic E_Cl_ = −40 mV and then it was changed the value of the resting potential (−60 mV). As shown in Fig 6I, with only shunting (E_Cl_ = −60 mV), greater G_Cl_ was required to block spikes and was proportional to G_Na_. This indicates that membrane depolarization, and the subsequent voltage-dependent inactivation of Na^+^ channels, contributes to the lower safety factor for spike propagation in the presence of GABA_A_ receptor activation. Indeed, when we examined the inactivation state variable for the Na^+^ conductance, the fractional available Na^+^ conductance at the t-junction decreased with increasing somatic G_Cl_ (S13 Fig). Taken together, the morphometry of DRG neuron stem axons and our biophysical models support the hypothesis that the longer stem axons of A-fibers contribute to limiting the influence of somatic GABA_A_ conductance on spike filtering. That said, expression of G_Cl_ in the axons (stem, SN, and DR) of the models was more effective at inactivating Na^+^ channels, compared to when they were only expressed at the soma (S13 Fig, bottom graphs in panels A and B). Additionally, greater shunting at, and proximal to, the t-junction lower the safety factor for spike propagation. Thus, under conditions with homogenous distribution of GABA_A_ channels in the soma and axons, and the uniform concentrations of GABA in the extracellular space, the axonal GABA_A_ channels would have stronger influence over the t-junctional filtering than somatic GABA_A_ channels. However, if GABA is released onto the soma mostly (i.e., across the satellite glia septum or in an autocrine way), then the effectiveness of electrotonic coupling of the soma to the t-junction would again be dependent on the stem axon length. In this vein, long stem axons of A fibers may physically remove the t-junctions away from the somatic GABA release area, making axonal GABA_A_ channels near the t-junction less relevant.

### Evidence for tonic release of GABA within the DRG

**Fig 7:**
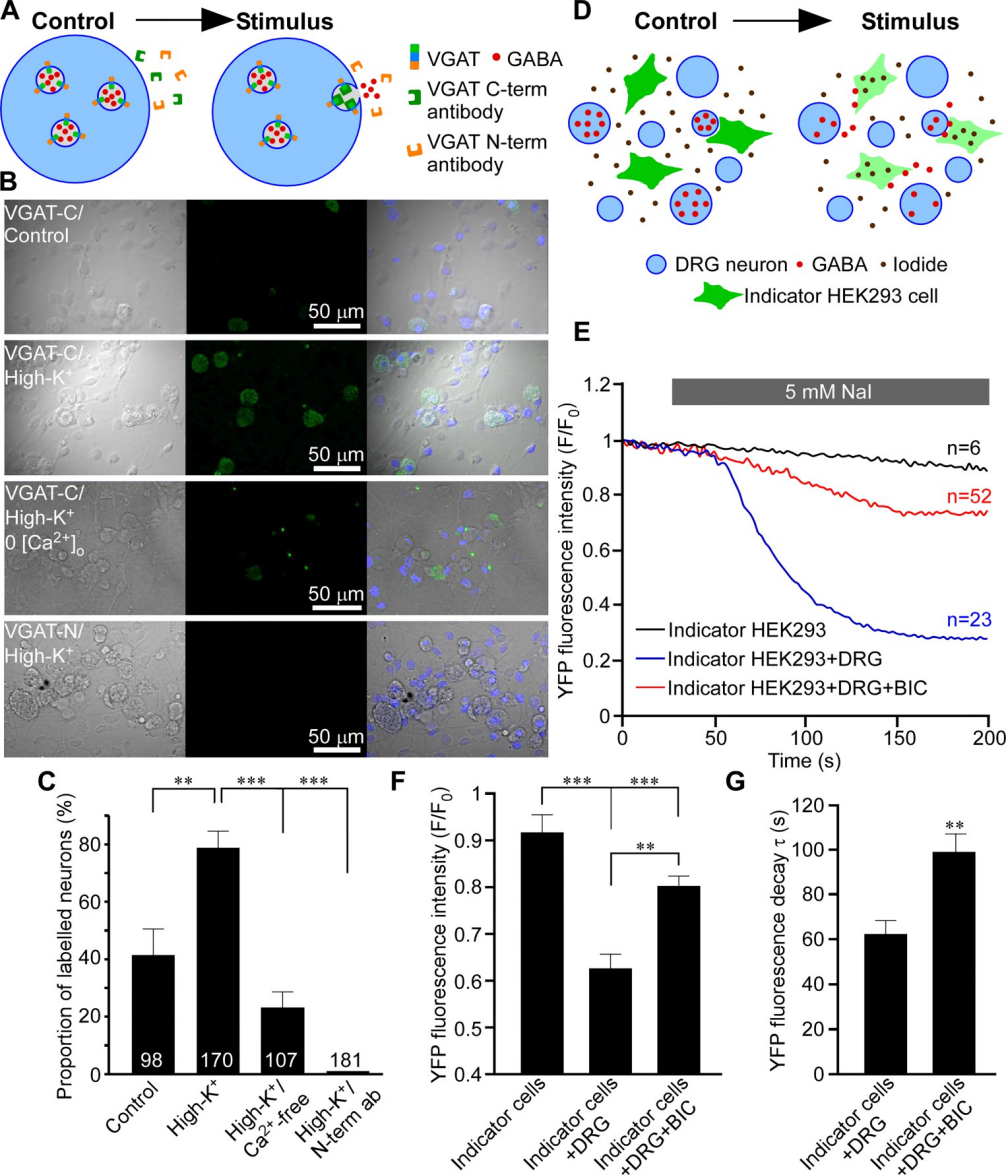
Activity-dependent and spontaneous release of GABA from DRG neurons. (**A–C**) Vesicular GABA release from DRG neurons studied with luminal VGAT antibody uptake. (**A**) Schematic of activity-dependent uptake of luminal (C-terminal) VGAT antibody. (**B**) Bright-field (left), fluorescence (middle), and overlaid images of DRG culture incubated in the presence of either a luminal VGAT antibody (C-term; 3 upper rows) or a cytoplasmic (N-term; bottom row) antibody. Cells were incubated in the control extracellular solution (upper row) or depolarized by extracellular solution containing 100 mM KCl (second from the top and the bottom rows). In the second from the bottom row, Ca^2+^ was excluded from the extracellular solution. (**C**) Summary of experiments like these shown in B (*N* = 3–5, number of neurons is indicated within the columns); quantified is the percentage of stained neurons **p < 0.01, ***p < 0.01 (Fisher’s exact test with Bonferroni correction). (**D–G**) GABA release from DRG investigated with the use of indicator HEK293 cells. (**D**) Schematic of the experiment: HEK293 cells transfected with α1, β2, and γ2 subunits of GABA_A_ receptors and a halide-sensitive EYFP mutant (H148Q/I152L; EYFP-QL) are co-cultured with DRG neurons and imaged in the presence of 5 mM extracellular iodide. When GABA is present in the extracellular solution, GABA_A_ channel opening causes I^-^ influx and EYFP-QL fluorescence quenching. (**E**) Application of NaI induced bicuculline-sensitive EYFP-QL quenching only when indicator HEK293 cells are co-cultured with DRG but not in monoculture; representative traces are shown for each experimental condition. (**F**) Summary for panel E; normalized fluorescence intensity is quantified at 200 s of each recording. Kruskal–Wallis ANOVA: H(2) = 33.6, *p* < 0.001; Mann–Whitney post hoc test: significant difference between indicated groups, **p < 0.01, ***p < 0.001. (**G**) Quantification of the kinetics of the EYFP-QL fluorescence quenching in experiments shown in E; individual recordings were fit with exponential function and time constants (τ) of the fluorescence decay were analyzed. Mann–Whitney test: U = 786, *p* < 0.01. In E and F, data from 3 independent experiments for each condition are shown, number of cells analyzed is indicated above each trace in panel E. Metadata for quantifications presented in this figure can be found at https://archive.researchdata.leeds.ac.uk/1042/. Schematics are drawn with Canvas X 2019. DRG, dorsal root ganglion.

Our paired recordings from SN and DR suggest the existence of tonic filtering at the DRG. At least a proportion of this tonic filtering is GABAergic (presumably due to a GABA tone), since BIC increases firing rate in the DR (Figs 1G and 1H and S1–S4), moreover, when injected to the DRG in vivo, BIC induces pain-like behavior [15]). In non-nociceptive fibers, this tonic filtering is much less pronounced (Fig 5). Here, we focused on the GABAergic mechanism; our previous data suggested that some DRG neuron cell bodies are capable of releasing GABA upon stimulation [15]. Another recent study reported robust activity-dependent somatic vesicle release from DRG neurons [43]. To investigate mechanisms of GABA release by DRG neurons, we developed a method for measuring exocytosis of GABA-containing vesicles based on the live uptake of luminal (C-terminal) antibody against vesicular GABA transporter VGAT (Fig 7A). N-terminus of VGAT, inserted in the neurotransmitter vesicle membrane, faces the cytoplasm while the C terminus resides in the vesicle lumen [44]. During exocytosis and subsequent recycling of a vesicle, luminal VGAT epitopes are temporarily exposed to the extracellular milieu. During such an exposure, antibodies that recognize these epitopes can bind to these and become trapped and subsequently internalized by endocytosis (Fig 7A; [44]). Antibodies against the N-terminus of VGAT should not be entrapped in this way as N-terminus of VGAT remains cytosolic at all times. Depolarization of cultured DRG neurons with extracellular solution containing 100 mM KCl induced robust uptake of C-terminal (luminal) but not N-terminal (cytosolic) VGAT antibody by DRG neurons (Fig 7B and 7C; quantified as proportions of neurons stained with the C-terminal VGAT antibody). The depolarization-induced C-terminal VGAT antibody uptake was significantly reduced (but not abolished—in good agreement with [43]) by the removal of extracellular Ca^2+^ (Fig 7B and 7C). Interestingly, even in the absence of depolarization, there was a significant number of neurons that took up C-terminal VGAT antibody (Fig 7B, top panel; C), hinting at spontaneous exocytosis of VGAT-containing vesicles. The N-terminal VGAT antibody was not taken up by DRG neurons (Fig 7B, bottom panel; C), even though this same antibody labeled permeabelized DRG neurons well [15].

Additionally, we used live imaging to optically monitor GABA release from the DRG neurons in culture. We transfected HEK293 cells with α_1_, β_2_, and γ_2_ GABA_A_ subunits and a halide-sensitive EYFP mutant (H148Q/I152L; EYFP-QL). The fluorescence of EYFP-QL is quenched by iodide and since Cl^-^ channels (e.g., GABA_A_) are permeable to this anion, EYFP-QL fluorescence quenching by I^-^ can be used to monitor Cl^-^ channel activation [45–48]. We co-cultured these “GABA indicator” HEK293 cells with DRG neurons and measured EYFP-QL fluorescence quenching induced by the addition of 5 mM NaI to the extracellular solution (Fig 7D). NaI induced robust EYFP-QL fluorescence quenching only when the indicator cells were co-cultured with DRG, not in the monoculture (Fig 7E). Moreover, this quenching in the DRG/indicator cell co-culture was significantly reduced in the presence of 50 μM BIC (Fig 7E and 7F). BIC also significantly slowed down the kinetics of the EYFP-QL fluorescence intensity decay (Fig 7G). These data further support existence of GABA tone within the sensory ganglia.

## Discussion

There is substantial evidence indicating that DRGs regulate the flow of sensory information to the CNS [13,14,18,19,49–52]. Consistent with this, electrical stimulation of DRGs provides strong analgesia even to the most intractable types of chronic pain in humans [53–55]. Simulations and experiments point to axonal t-junctions within DRGs as biophysically amenable sites for peripheral filtering/gating [14–17,49]. Moreover, GABAergic signaling within the DRG is emerging as a potential mechanism regulating such filtering. Thus, DRG neurons can produce and release GABA [15,23] and GABA delivered directly into the DRG has prominent analgesic action [15,22].

Our main findings here are 3-fold. First, we demonstrate that the DRG exerts a dynamic control over the throughput conduction via GABA receptor activation. Indeed, focal application of GABA, or optogenetic stimulation of GABA release from the DRG-transplanted MGE cells, specifically reduced stimulus-induced firing frequencies in the DR but not the SN in various experimental settings. Second, we show that this GABAergic filtering is much more efficient in the C-fibers, as compared to A-fibers. Third, we demonstrate that an intrinsic GABAergic inhibitory system can be engaged to scale the filtering of spikes passing through the DRG up or down, modulating nociceptive input to the CNS. Importantly, we further show that this mechanism can significantly reduce sensitivity to noxious stimuli and provide effective relief of hypersensitivity observed in chronic pain models.

The t-junctions, where the stem axon bifurcates into SN and DR axons, are expected to have a lowered safety factor for AP propagation due to the impedance mismatch [13–15,32,51,56]. Our earlier biophysical model of a C-fiber predicted that opening of somatic/peri-somatic GABA_A_ channels increases the likelihood for the failure of action potential propagation through the t-junction [15]. Here, we provide strong experimental support to these predictions. We show that GABA receptor activation within the DRG reduces the firing rate recorded at a point after the t-junction and has no effect on spikes entering the DRG. The most straightforward interpretation of these observations is that evoked spikes in peripheral axons fail to propagate through the DRG into DR axons when GABA system is activated. According to earlier modeling, the failure maybe due to a combination of t-junction geometry (i.e., input impedance drop), GABA conductance shunting, and GABA-induced depolarization and subsequent voltage-gated sodium channel inactivation [15]. In this study, we focused specifically on GABAergic filtering but this does not imply that t-junctional filtering cannot be mediated or regulated by other mechanisms. For example, activation of somatic or stem axon/t-junction K^+^ channels has also been suggested as a mechanism promoting t-junctional spike failure [14,19].

Another striking finding is that GABAergic DRG filtering is much more robust in C-, as compared to the A-fibers. As a consequence, optogenetic GABA release (S9 Fig) or DRG injection of GABA (S10 Fig) specifically inhibited firing produced by noxious but not innocuous stimuli. A recent study used single-unit recordings from the DR to demonstrate that electric field stimulation of the DRG in rats, which mimicked DRG field stimulation (neuromodulation) used for analgesia in humans [53–55], increased t-junctional filtering mostly in C-fibers, with little effect in A-type fibers [19]. Together, these findings indicate that C fibers are more amenable for the t-junctional filtering, which can be exploited therapeutically.

We propose that the induced filtering is more efficient in the C-fibers in part, because these have a shorter stem axon. Electrotonic coupling of the soma to the t-junction depends on the stem axon length [15,17]. Drawings by Ramon y Cajal indeed suggested much shorter stems in small-diameter DRG neurons, as compared to the larger ones [34]. Our light-sheet imaging of cleared DRG with labeled C- and A-type fibers established that stem axon is indeed over 3 times shorter in C fibers (Fig 6). Our new biophysical model of an A-fiber suggests that when a stem axon is longer than 200 to 300 μm, opening of somatic GABA_A_ channels has less effect on the action potentials propagating through their t-junctions. Thus, this investigation may have revealed a simple but elegant principle of differential filtering of spikes that depends on the modality-specific fiber morphology. It should be emphasized, however, that reduced excitability at the t-junction, not the impedance mismatch alone, contributes to GABA-dependent spike failure.

Interestingly, there was considerable basal activity in the nerve under control (no peripheral stimulation) conditions. This may arise from the activity of low-threshold, non-nociceptive neurons or from the preparation-specific injury. However, our previous observation that DRG-injected GABA antagonists in freely behaving animals induced pain-like paw flinching in the absence of noxious input [15] may suggest that there is indeed some spontaneous activity in the peripheral nerve that is failing to reach spinal cord. Of note, basal firing rates in the DR were almost invariantly about 50% lower, as compared to these in SN, even under conditions where efferent fibers were either cut (S1 Fig) or physically destroyed (S2 Fig). Single-unit recordings (Fig 5) revealed that evoked spikes in C and A fibers do fail occasionally under basal conditions (at a rate of approximately 16% in C and approximately 6% in A fibers), which was consistent with a recent report [18]. Thus, basal filtering does exist but when measuring evoked single-unit spikes it is much lower than approximately 50% mismatch seen in our multi-unit SN/DR recordings. While GABA_A_ antagonist BIC significantly reduced this mismatch indicating that at least part of it is due to tonic GABAergic inhibition, we cannot exclude that other factors may contribute to the mismatch, including, e.g., some back-propagation in the SN. It is important to note, however, that regardless of the nature of this mismatch in basal firing, the sensory stimulation of the paw (noxious or innocuous) is seen in our SN/DR recordings as a consistent increase in the firing rates in both compartments (SN and DR).

Another important question—is the intrinsic GABAergic system in the DRG sufficient to impose filtering in vivo? Strong expression of functional GABA_A_ receptors in DRG is well recognized (reviewed in [57]) but the ability of DRG to produce and release GABA is less well documented (although see [15,23]). Transcript levels of enzymes (GAD65, GAD67) and transporters (VGAT) needed to synthesize and package GABA into vesicles are low according to transcriptomic studies [58,59]. Yet functional proteins are detectable [15,23,60], as is tonic and induced GABA release (Fig 7 and [15]). Moreover, in vivo studies demonstrated that GABA reuptake inhibitor, NO711, produced analgesic effect when delivered to the DRG [15,22], while DRG-applied GABA_A_ antagonists exacerbated peripherally induced pain [15]. Hence, there must be efficacious levels of endogenous GABA in the DRG influencing action potential throughput.

A major causative factor of neuropathic pain is a loss of dorsal horn GABAergic inhibitory system [61]. GABAergic progenitor cells implanted into adult spinal cord survive and integrate into the spinal inhibitory circuit [27,62,63], reducing neuropathic pain severity [26,64]. We found the MGE cells transplanted into the DRG of adult mice also survive there and can release GABA (Fig 3). Optogenetic stimulation of MGE-transplanted DRG significantly alleviated hypersensitivity to noxious stimuli induced by chronic inflammation (Fig 3). Importantly, even without optogenetic stimulation, MGE cell transplantation accelerated the recovery from both inflammatory and neuropathic types of such hypersensitivity (S8 Fig). This further suggests that GABAergic system in DRG could be targeted for pain relief. In the spinal cord, MGE cells maturate into interneurons and integrate into the existing spinal inhibitory system [26,27]. For the case of the DRG transplant, the direct analogy is unlikely as there are no “classical” interneurons in DRG. The most straightforward explanation for the anti-nociceptive effect of MGE cells in DRG is a “GABA pump” mechanism, whereby MGE cells simply release GABA into the extracellular space thus increasing the GABA tone. Recent years saw increasing success in using stem cells as a chronic pain therapy [65,66] and GABAergic progenitor cells can be generated from human stem cells [67] and integrated into the pain pathways [68]. Thus, targeting GABAergic system with the DRG-directed stem cell therapy or GABA-mimetics tailored to reduce their CNS permeability could open up avenues for analgesic strategies with reduced CNS side effects.

### Study limitations

It must be noted, that while our approach to matching spikes between SN and DR recording sites is largely accurate in simulations, it does have some real-world limitations. The accuracy of matching decreases slightly when the firing rate increases, and also with very slow conduction velocities. Some variability may also arise from different electrode characteristics between the recording sites and signal to noise ratios that affect spike extraction in the first instance, in addition to variability in the unsupervised method of spike sorting. However, with these considerations in mind and ensuring a short distance between recording sites, it is perfectly feasible to temporally correlate spikes from peripheral and central nerves across dorsal root ganglia t-junctions.

In this study, we provide for the first time quantitative measurements of stem axon lengths for both C- and A-fiber neurons, along with measurements of somatic diameter. But our modeling approach is limited by several critical membrane parameters. Mostly notably, all the membrane mechanisms (i.e., voltage-gated conductances) available in the literature are based on somatic measurements. We used minimal models with uniform distributions of key conductances along the neuronal membrane, as well as a uniform concentration of ions and extracellular GABA throughout the tissue. It is highly likely that there is much more variability within and between cells in vivo. Differential repertoires and densities of ion channels (including but not limited to GABA_A_) at the t-junctions or stem axon of different fiber types could indeed contribute to variations in filtering. Thus, in the absence of internodes, C fibers may have a higher GABA_A_ conductance density along their axons. Likewise, the density of Na channels may not be uniform, at least between the soma and axons [69,70]. There well may be other dynamic and activity-dependent differences in gating of action potentials at the t-junctions of different fiber types [71,72]. Future simulations, incorporating more experimentally determined parameters and physiological measurements, will be necessary to improve and validate our computational approach.

## Materials and methods

All animal experiments performed in Hebei Medical University were in accordance with the Animal Care and Ethical Committee of Hebei Medical University (approval number: IACUC-Hebmu-2020007). All animal work carried out at the University of Leeds was approved by the University of Leeds Animal Welfare Ethical Review Body (AWERB) and performed under UK Home Office License P40AD29D7 and in accordance with the regulations of the UK Animals (Scientific Procedures) Act 1986.

### In vivo recording of peripheral nerve and dorsal root activity

All surgical procedures were performed under deep anesthesia with an i.p. injection of pentobarbital sodium (60 to 80 mg/kg) in accordance with the Animal Care and Ethical Committee of Hebei Medical University under the International Association for the Study of Pain guidelines. In one set of experiments (S4 Fig), pentobarbital was replaced by isoflurane (4% for induction, 2% for maintenance). Laminectomy was performed to expose right L5 DRG of adult male rat (Sprague Dawley, 180 to 200 g) or L4 DRG of adult male C57BL/6J mice. DR, SN, and DRG were exposed by removal of both spinous and transverse processes of the vertebra bone; the DR and SN were then suspended (without transection) on the hooked stainless steel recording electrodes connected to BL-420F biological data acquisition and analysis system (Chengdu Techman Software Co., China). The wound was filled with paraffin in order to stabilize preparation. The right hindpaw was used for the injection of capsaicin (10 μM; 50 μl for rat; 20 μl for mouse) or Bradykinin (100 μM, 50 μl for rat; 20 μl for mouse), or the stimulation with hot water (approximately 60°C), ice, air puffs (using bulb syringe), von Frey filaments (4 g for rat; 0.4 g for mouse), or needle prick (glass electrode). GABA (200 μM; 3 μl for rat; 2 μl for mouse) or Bicuculline (200 μM; 3 μl for rat; 2 μl for mouse) was accurately delivered to the surface of exposed DRG by micropipettor.

For the single-unit recording in rats, DR and SN were exposed and covered with liquid paraffin. A single axon bundle was teased away from dorsal root by a fine tweezer and placed on nerve fiber electrode for electrophysiological recording. The stimulus current pulses (2 to 5 mA) were delivered to the spinal nerve at 10/50/100 Hz. Conduction velocity of fiber was determined by dividing conduction distance by response latency. GABA (200 μM, 3 μl), Tetrodotoxin (TTX, 1 μM, 3 μl), or vehicle (3 μl saline) were accurately delivered to DRG by micropipettor. During the operation and recording, the animal body temperature was maintained at 38°C with the Animal Temperature Controller pad (RWD Life Science Co., China).

### Ventral root transection and ChAT immunohistochemistry

Laminectomy was performed to expose right L5 DRG of adult male rat (Sprague Dawley, 180 to 200 g). VR was exposed by removal of both spinous and transverse processes of the vertebra and transected (5 mm of the nerve removed). The muscle and skin were sutured, area of the wound was disinfected with iodophor, and the animals were transferred to a recovery cage. Two weeks after VR transection, the rats were subjected to electrophysiological recordings or sacrificed for immunohistochemical detection of ChaT-positive motor fibers. In the latter case, the spinal nerve sections of L5 DRGs were removed and submerged in Tissue-Tek O.C.T. (Sakura, Alphen aan den Rijn, the Netherlands), frozen, and sectioned (10 μm) using a freezing microtome (CM1950, Leica Microsystems). Sections were placed on microscope slides and washed thrice with 0.01 M PBS (Sigma-Aldrich), fixed in 4% PFA (Biosharp) for 1 h, and blocked for 2 h with blocking buffer (3% donkey serum in 0.1 M PBS; Sigma-Aldrich). Primary antibodies (ChAT, Abcam, 1:400) were diluted in 0.3% Triton X-100/PBS buffer before overnight incubation at 4°C. The following day, sections received a further 3 washes in PBS before incubation with secondary antibodies (Alexa Fluor 488, 1:500) for 2 h at room temperature. Sections were washed with PBS 3 times and sealed with cover glass. Staining was visualized using a confocal fluorescent microscope (TCS SP5 II, Leica).

### Spike sorting

Electrophysiological recordings of both the SN and DR were imported into Python and high pass filtered at 60 Hz using a digital Butterworth filter from the SciPy module [73]. Extracellular spike times and waveforms were extracted using an absolute median deviation of between 5 and 6 from the median of the signal. Extracted spike waveforms were sorted using the WaveClus program [74] in Matlab to define individual neuronal units underlying the extracellular signal. Matching of spikes in the dorsal root to an origin spike in the spinal nerve was achieved by finding the minimum latency (within a tolerance window of the slowest theoretical fiber conduction velocity of 0.1 m/s) between spikes in the SN and DR. The overall approach to spike sorting was as follows: (i) The SN spike waveforms were sorted to group SN spikes into “firing units/clusters.” (ii) For every spike in DR, we tried to identify an origin spike in the SN, based on minimal latency with a cut-off of 150 ms (calculated from the slowest expected conduction velocity of C fibers of 0.1 m/s over the estimated distance between 2 recording electrodes of 1.5 cm). (iii) The spike identity, defined by the SN spike waveforms, was extended to the DR spikes after matching. (iv) For each firing unit/cluster, the propagation success was quantified based on how many SN spikes in that firing unit had a matched DR spike (number of SN spikes with DR match/total SN spikes) × 100.

To validate this approach, we used a computer-generated, Poisson randomly generated spike train to simulate an SN recording (S5 Fig). The DR spike train was produced by firstly randomly assigning “spikes” in the simulated SN spike train a “fiber type” from Aα, Aβ, Aδ, and C. To introduce randomness and variability, each SN “spike” was randomly assigned a conduction velocity from a normal distribution of the expected mean conduction velocity for the assigned fiber type. The randomly generated conduction velocity for each SN spike dictated the latency with which an associated spike should be placed in the simulated DR spike train as if it was recorded 1.5 cm away from the simulated spinal nerve spike train (as per our in vivo recording conditions). To evaluate the performance of spike-matching during t-junction filtering, spikes in the DR were randomly deleted to simulate different degrees of filtering. Accuracy was defined as the percentage of correct matches from total “ground truth” matches (S5B Fig). We hypothesized that this latency-based spike-matching method would decrease in accuracy as the firing rate increased as this would increase the likelihood of spikes, traveling at different velocities, overlapping in the DR spike train. In a similar vein, we also hypothesized that the accuracy would be determined by the slowest conducting fiber as this again would increase the likelihood of faster spikes overlapping with the slower spikes. Both predications were indeed confirmed. We found that the accuracy of our method was reduced to 80% if a very slow conducting mean C fiber velocity was used to randomly shift dorsal root spike latencies and also at higher spinal nerve firing rates (S5B Fig). A false positive in this case was more likely to be a faster spike overlapping with a slower spike, i.e., incorrectly finding a match for a slow spinal nerve spike. We simulated spinal nerve firing rates up to 100 Hz but none of the in vivo spike-sorted recordings approached this firing rate giving us confidence that little overlap was occurring and that our method was accurate (>80%) at our observed firing rates. We also independently adjusted the mean conduction velocities of other fiber types and found no effect on accuracy. To further increase randomness in our assessment of this method, each simulation experiment (where a fiber type mean conduction velocity was adjusted, at varying spinal nerve firing rates and degrees of random deletion) was repeated 5 times with new randomly generated Poisson spike trains, with new randomly assigned conduction velocities. Source data and code for spike sorting analysis is available at GitHub (https://github.com/pnm4sfix/SpikePropagation).

### MGE cells transplantation

The female VGAT-ChR2-EYFP mice (Jackson Laboratory) with embryos between E12.5 and E13.5 were anesthetized with an intraperitoneal injection of pentobarbital sodium (60 to 80 mg/kg). All the embryos were removed via abdominal incision and placed into a 10-cm Petri dish containing ice-cold HBSS. The mice were humanely sacrificed. The gestational sac of each embryo was removed using forceps under the stereo microscope (SZX7, Olympus). The embryonic brain was extracted and cut along the sagittal plane to separate 2 hemispheres. MGE on each hemisphere was then removed with a scalpel. MGE tissue was put in a 1.5 ml collection tube containing 500 μl DMEM/10% FBS (Sigma) and triturated into a single-cell suspension as described [75]. The final cell density in the suspension of embryonic stem cells was measured with hemocytometer. Adult (5 to 6 weeks) male C57BL/6J mice were anesthetized with an intraperitoneal injection of pentobarbital sodium (60 to 80 mg/kg). L4 DRG was exposed by removal of both spinous and transverse processes of the vertebra bone. The microinjector (Hamilton Co.) loaded with a suspension of MGE cells (3 μl; approximately 1 × 10^7^/ml) was inserted into the ganglion to a depth of 200 μm from the exposed surface. The cell suspension was injected slowly, and the needle was removed 3 min after the injection. The muscles overlying the spinal cord were loosely sutured together, and the wound was closed. Animals developing signs of distress were humanely sacrificed. In order to verify that the DRGs were transplanted with MGE cells successfully, the L4 DRGs were excised, submerged in Tissue-Tek O.C.T. (Sakura, Alphen aan den Rijn, the Netherlands), frozen, and sectioned (10 μm) using a freezing microtome (CM1950, Leica Microsystems). Slices were then analyzed for the EYFP fluorescence using confocal microscopy (TCS SP5 II, Leica Microsystems).

### Chronic pain models

CCI was performed as described previously [15]. Briefly, rats were anesthetized with an i.p. injection of sodium pentobarbital (60 to 80 mg/kg). The right hind leg was shaved and cleaned using 70% ethanol. The sciatic nerve was exposed by blunt dissection at the mid-thigh level, proximal to the sciatic trifurcation. Four nonabsorbable sterile surgical sutures (4–0 chromic gut; packaged in isopropyl alcohol and soaked in saline for 10 min prior to application) were loosely tied around the sciatic nerve with an approximately 1.0-to-1.5-mm interval between the knots. The skin was sutured, and the animal was transferred to a recovery cage. To induce chronic inflammatory pain, CFA (20 μl) was injected into the plantar surface of the right hind paw of the mice.

### Behavioral tests

Mechanical withdrawal threshold was measured by a set of von Frey filaments (Stoelting Co., Chicago, Illinois, United States of America) with a calibrated range of bending force (0.16, 0.4, 0.6, 1, 1.4, 2 g). Each mouse was placed into a plastic cage with a wire mesh bottom. A single filament was applied perpendicularly to the plantar surface of hind paw for 5 times with an interval of 5 s. Positive response was defined as at least 3 clear withdrawal responses out of 5 applications. Filaments were applied in an up-and-down order according to a negative or positive response to determine the hind paw withdrawal threshold. Thermal withdrawal latency was tested by a radiant heat lamp source (PL-200, Taimeng Co., Chengdu, China). The intensity of the radiant heat source was maintained at 10 ± 0.1%. Mice were placed individually into Plexiglas cubicles placed on a transparent glass surface. The light beam from radiant heat lamp, located below the glass, was directed at the plantar surface of hindpaw. The time was recorded from the onset of radiant heat stimulation to withdrawal of the hindpaw. Three trials with an interval of 5 min were made for each mouse, and scores from 3 trials were averaged.

### In vivo optogenetic stimulation

Adult male C57BL/6J mice were L4 DRG transplanted with MGE cells from VGAT-ChR2-EYFP mice 3 to 4 weeks before experiments. Recordings of DR and SN activity was performed as described above in combination with laser stimulation (473 nm, 3 mW, 30 Hz for 10 s with 20-s interval) of DRG using an MLL-FN-473-50 unit (Changchun New Industries Optoelectronics Technology Co.) controlled by a pulsing set (S48 Stimulator, Grass Technologies, An Astro-Med, Product Group). In the behavioral tests on freely moving animals, a stainless steel cannula guide (RWD Life Science Co., China; diameter 0.64 mm) was implanted into the L4 DRG, the cannula was firmly fixed in place with dental cement, and the optical fiber (RWD Life Science Co., China; diameter 0.2 mm, length 1 m) was inserted through the guide; a more detailed description is provided in [15].

### Patch clamp recording from DRG neurons

DRG dissection were performed as described previously [42]. Briefly, L4 DRG transplanted with MGE cells 4 weeks in advance was carefully removed and digested with a mixture of 0.4 mg/ml trypsin (Sigma) and 1.0 mg/ml type-A collagenase (Sigma) for 45 min at 37°C. The intact ganglia were then incubated in ACSF (artificial cerebrospinal fluid) oxygenated with 95% O2 and 5% CO2 at 28°C for at least 1 h before transferring them to the recording chamber. DRG were visualized with a 40× water-immersion objective using a microscope (BX51WI; Olympus, Tokyo, Japan) equipped with infrared differential interference contrast optics. Whole-cell current recording was acquired with an Axon700B amplifier (Molecular Devices Corporation, Sunnyvale, California, USA) and pClamp 10.0 software (Axon Instruments); recordings were sampled at 5 kHz. Patch pipettes (4 to 7 MΩ) were pulled from borosilicate glass capillaries on P-97 puller (Sutter Instruments, USA). The series resistance was 10 to 20 MΩ. Continuous voltage-clamp recording was performed via holding potential of −60 mV. The ACSF contained (in mM): 124 NaCl, 2.5 KCl, 1.2 NaH_2_PO_4_, 1.0 MgCl_2_, 2.0 CaCl_2_, 25 NaHCO_3_, and 10 Glucose. The pipette solution contained (in mM): 140 KCl, 2 MgCl_2_, 10 HEPES, 2 Mg-ATP, pH 7.4. Osmolarity was adjusted to 290 to 300 mOsm. For optical stimulation of MGE cells derived from VGAT-ChR2-EYFP mice, a 473-nm blue light (3 mW) was elicited using the same device as for in vivo stimulation.

### VGAT antibody uptake

DRG neurons were dissociated and cultured as described previously [42]. DRG neurons were incubated for 15 min in either normal or “high K^+^” extracellular (EC) solution supplemented with either C-terminal (luminal) N-terminal (cytosolic) VGAT antibodies. Normal EC solution contains (in mM): 144 NaCl, 5.8 KCl, 1.3 CaCl_2_, 5.6 D-glucose, 0.7 NaH_2_PO4, 0.9 MgCl_2_, and 10 HEPES (all from Sigma). In high K^+^ EC solution NaCl concentration was lowered to 49.8 mM and KCl concentration was raised to 100 mM. Ca^2+^-free EC solution was also used; in this solution, CaCl_2_ was omitted. After incubation, cell cultures were washed 3 times with PBS and fixed using 4% paraformaldehyde, followed by permeabilization with 0.05% tween 20 and 0.25% triton-X 100 (with donkey serum) for 1 h. Cells were then labeled with secondary antibody, washed 3 times with PBS, mounted on coverslips, and imaged using Zeiss LSM880 confocal microscope. The following antibodies were used: VGAT C-terminal antibody (rabbit polyclonal #AB-N44, Advance Targeting System; 1:200); VGAT N-terminal antibody (rabbit polyclonal 131002, Invitrogen, Eugene, Oregon, USA; 1:1,000); secondary antibody: alexafluor donkey anti-rabbit 488 (Invitrogen, Eugene, Oregon, USA; 1:1,000). Cells were deemed positively stained if the mean fluorescence intensity of the somatic area was at least 2 times of the background fluorescence intensity.

### Iodide imaging

HEK293 cells were co-transfected with cDNA encoding human α1, β2, and γ2 subunits of GABA_A_ receptors (gift of David Weiss, Department of Physiology, University of Texas Health Science Center, San Antonio, Texas, USA) together with the halide-sensitive EYFP mutant (H148Q/I152L; EYFP-QL) using FuGENE HD transfection reagent. Transfected cells were co-cultured for 24 h with DRG neurons isolated as described above (see also [15]). Extracellular solution consisted of (mM): NaCl (160); KCl (2.5); MgCl_2_ (1); CaCl_2_ (2); HEPES (10), and glucose (10); pH adjusted to 7.4 with NaOH (all from Sigma). I^-^ containing solution was produced by equimolar substitution of 5 mM NaCl with NaI. I^-^ imaging was performed using Nikon TE-2000 E Swept Field Confocal microscope using 488 nm argon laser as excitation light source. Images were recorded and analyzed using Nikon Elements software.

### Dorsal root ganglia clearing, staining, and morphometry

DRGs were extracted from the lumbar spinal column of euthanized adult Wistar rats (150 to 250 g) and immersion fixed in 4% paraformaldehyde at 4°C overnight. Tissue clearing of the ganglia was performed using the iDISCO+ protocol [37]. Briefly, DRG samples were pretreated and permeabilized before blocking with 3% donkey serum. Primary antibodies raised against Neurofilament-200 (NF-200, 1:500, Sigma-Aldrich, N5389) and peripherin (Abcam, 1:250, ab39374) were used as markers for myelinated neurons and nociceptors, respectively. DRGs were incubated in primary antibodies for 5 days at 37°C, washed for 24 h, and incubated in secondary antibodies (donkey anti-mouse 555, 1:1,000, donkey anti-chicken 488, 1:1,000, Invitrogen, A31570, A21202) for 4 days at 37°C. Following final washes, DRGs were embedded in 1% agarose cubes, sequentially dehydrated in a methanol/H_2_0 series (20%, 40%, 60%, 80%, 100%, 100%), and stored overnight in 100% methanol at room temperature. Following methanol dehydration, samples were incubated for 3 h in 33% methanol/66% dichloromethane (DCM, Sigma-Aldrich) solution. To complete the tissue clearing, this was followed by two 15-min incubations in 100% DCM and final storage in DiBenzyl Ether (DBE, Sigma-Aldrich).

Cleared DRG samples were imaged using the 20× objective of an Ultramicroscope II (LaVision BioTech) resulting in an axial resolution of 0.35 × 0.35 μm. The numerical aperture of the light sheet was 0.15 and resulted in a light sheet focal thickness of 4 μm. Dynamic horizontal focusing was utilized to ensure a homogenous point spread function across the field of view. Images were analyzed using the simple neurite tracer in FIJI [76,77]. Using this semi-automatic method of tracing, stem axons were measured from origin at peripherin and NF-200-positive somata through the image stack until clear bifurcation or in the case of NF-200-positive cells, loss of a clear signal.

### Computer modeling

All simulations were performed using NEURON [78] on an Intel-based Macintosh computer (http://neuron.yale.edu) and analyzed using Python scripts. Representative code is available at GitHub (https://github.com/dbjaffe67/DRGsims). A simplified model of a portion of an Aδ neuron within the DRG was constructed of an SN and DR axon of 21 mm length of 4 and 3 μm diameter, respectively, of alternating internodal (150 μm length) and nodal (1.5 μm) segments (3 μm diameter) [79]. A stem axon was connected to the t-junction node midway along the SN/DR axis. Stem axon internode distance was 100 μm with nodes of 1.5 μm, both with diameters ranging from 1 to 3 μm. An unmyelinated axon initial segment (AIS) [80] of 50 μm and 2 μm diameter was attached to the last stem node and the soma (40 μm diameter [81]). All internodal/myelin wrapped segments had a specific membrane conductance (G_m_) of 10 μS/cm^2^ and a specific membrane capacitance (C_m_) of 0.01 μF/cm^2^, while for the nodes, AIS, and soma the values for G_m_ and C_m_ were 200 μS/cm^2^ and 1 μF/cm^2^, respectively. Soma and nodes contained a TTX-sensitive voltage-gated Na^+^ conductance (G_Na_) reflecting a mix of Na_V_1.1, 1.6, and 1.7 channels [39]. Spike repolarization in nodes was achieved solely by passive leak conductance [82], while both the AIS and soma also contained a delayed rectifier conductance (5 mS/cm^2^). A chloride conductance (G_Cl_) with E_Cl_ = −40 mV [42] was added to model tonic GABA_A_ receptor activation in various compartments (i.e., soma and/or axons). Resting potential was set in all compartments to −60 mV [14,81] and used to calculate the resting leak equilibrium potential from the sum of steady-state resting currents. Action potentials were initiated by injecting suprathreshold current (1 ms duration) into the most distal portion of the spinal nerve axon. Morphology for an unmyelinated C fiber neuron, used for comparison with the A-neuron, was based on previously published models [15] constructed with SN/DR axons of 5.5 mm each with diameters of 0.8 and 0.4 μm, respectively. The stem axon was connected at the midpoint with a length ranging from 25 to 150 μm and diameters 1 to 2 μm. The same TTX-sensitive voltage-gated Na^+^ conductance was added to all compartments and spike repolarization was achieved by a delayed rectifier K^+^ conductance [40]. Specific membrane resistivity for all compartments was 10,000 Ωcm^2^, intracellular resistance 100 Ωcm, and capacitance of 1 μF/cm^2^.

### Statistics

Sample size estimations were made using NC3Rs Experimental Design Assistant (https://eda.nc3rs.org.uk/). Animals were allocated to the groups randomly. In the instances where blinding was appropriate (e.g., in vivo optogenetic stimulation of implanted MGE cells), the operator was unaware of specific surgery allocations. All data are given as mean ± SEM. Paired or unpaired *t* test was used to compare the mean of 2 groups when the data were normally distributed. Multiple groups were compared using ANOVA (one-, two-, or three-factor) or repeated-measures ANOVA, depending on experimental setup; Bonferroni post hoc test was used for comparison between groups; for data failing normality test Kruskal–Wallis ANOVA with Mann–Whitney post hoc test was used. Statistical analyses were performed using IBM SPSS Statistics 21, GraphPad Prism, or Origin. Statistical parameters are given in the figure legends.

## Supporting information

S1 Fig Ventral root transection does not affect filtering at the DRG. (**A**) VR transection (schematized in the panel J) does not affect spontaneous activity in the SN or DR. (**B**) Summary of experiments exemplified in A. Two-factor (nerve site, VR transection) repeated measures ANOVA: main effect associated with nerve site [F(1,12) = 12.7; *p* < 0.05]. (**C**) After the VR transection, a baseline was recorded (control) and Capsaicin (CAP, 10 μM, 50 μl) was injected into the hindpaw. CAP increased firing frequency in both SN and DR branches of the nerve (middle traces, as compared to basal activity shown in the upper traces). Application of GABA (200 μM, 3 μl) to DRG reduced CAP-induced firing frequency in DR but not SN (bottom traces). (**D**) Summary of the panel C. Two-factor (nerve site, drug application) repeated measures ANOVA: main effects associated with nerve site [F(1,10) = 12.2; *p* < 0.05] and drug application [F(2,9) = 6.5; *p* < 0.05]; significant interaction between nerve site and drug application [F(2,9) = 41.3; *p* < 0.01]. Bonferroni post hoc test: **,***significant difference from control (*p* < 0.01, *p* < 0.001); ^##^significant difference from CAP (*p* < 0.01). (**E**) After the VR transection, a baseline was recorded (control) and GABA_A_ antagonist bicuculline (BIC, 200 μM, 3 μl) was applied to DRG; hindpaw was not stimulated. (**F**) Summary for panel E. Two-factor repeated measures ANOVA: main effects associated with nerve site [F(1,12) = 8.2; *p* < 0.05], drug application [F(1,12) = 18.2; *p* < 0.01], significant interaction between nerve site and drug application [F(1,12) = 8.1; *p* < 0.05]. Bonferroni post hoc test: **significant difference from control (*p* < 0.01). (**G**) Experiments similar to these shown in (E, F) but GABA was applied instead of BIC. (**H**) Summary for panel G. Two-factor repeated measures ANOVA: main effect associated with nerve site [F(1,10) = 11.9; *p* < 0.05). (**I**) Scatter plot comparison of the firing basal (tonic) firing rates in paired SN-DR recordings with VR intact (left, data from the Fig 1; paired *t* test: t(25) = 7.2, *p* < 0.001) and VR transected (right; paired *t* test: t(36) = 6.8, *p* < 0.001). (**J**) Schematic of the preparation. The overall procedure is similar to that in Fig 1 but VR is transected before (or during) the recording. Schematics are drawn with Canvas X 2019. Metadata for quantifications presented in this figure can be found at https://archive.researchdata.leeds.ac.uk/1042/.
(PDF)

S2 Fig Efferent fiber elimination does not affect filtering at the DRG. The ventral root was surgically transected as in S1 Fig. The animals were left to recover and further experiments were done 2 weeks after the transection. (**A**) Immunostaining of the L5 spinal nerve of control (right) and VR-transected animals for choline acetyltransferase. (**B**) Two weeks after the VR transection, a baseline was recorded (control) and Capsaicin (CAP, 10 μM, 50 μl) was injected into the hindpaw. CAP increased firing frequency in both SN and DR branches of the nerve (middle traces, as compared to basal activity shown in the upper traces). Application of GABA (200 μM, 3 μl) to DRG reduced CAP-induced firing frequency in DR but not SN (bottom traces). (**C**) Summary for panel B. Two-factor (nerve site, drug application) repeated measures ANOVA: main effects associated with nerve site [F(1,12) = 15.8; *p* < 0.01] and drug application [F(1,12) = 34.8; *p* < 0.01]; significant interaction between nerve site and drug application [F(2,11) = 7.7; *p* < 0.05]. Bonferroni post hoc test: *,***significant difference from control (*p* < 0.05, *p* < 0.001); ^##^significant difference from CAP (*p* < 0.01). (**D**) GABA_A_ antagonist bicuculline (BIC, 200 μM, 3 μl) was applied to DRG; hindpaw was not stimulated. (**E**) Summary for panel D. Two-factor repeated measures ANOVA: main effects associated with nerve site [F(1,12) = 12.9; *p* < 0.05], drug application [F(1,12) = 52.9; *p* < 0.001], significant interaction between nerve site and drug application [F(1,12) = 14.8; *p* < 0.01]. Bonferroni post hoc test: ***significant difference from control (*p* < 0.001). Metadata for quantifications presented in this figure can be found at https://archive.researchdata.leeds.ac.uk/1042/.
(PDF)

S3 Fig DRG filtering in the female rats. The recordings similar to that shown in Fig 1 but performed in female rats. (**A**) After baseline was recorded (control), Capsaicin (CAP, 10 μM, 50 μl) was injected into the hindpaw. Application of GABA (200 μM, 3 μl) to DRG reduced CAP-induced firing frequency in DR but not SN (bottom traces). (**B**) Summary for panel A. Two-factor (nerve site, drug application) repeated measures ANOVA: main effect associated with nerve site [F(1,10) = 8.7; *p* < 0.05]. Bonferroni post hoc test: *,**significant difference from control (*p* < 0.05, *p* < 0.01); ^#^significant difference from CAP (*p* < 0.05). (**C**) GABA_A_ antagonist bicuculline (BIC, 200 μM, 3 μl) was applied to DRG; hindpaw was not stimulated. (**D**) Summary for panel C. Two-factor repeated measures ANOVA: main effects associated with nerve site [F(1,10) = 10.4; *p* < 0.05], drug application [F(1,10) = 12.2; *p* < 0.05], significant interaction between nerve site and drug application [F(1,10) = 23.1; *p* < 0.01]. Bonferroni post hoc test: **significant difference from control (*p* < 0.01). Metadata for quantifications presented in this figure can be found at https://archive.researchdata.leeds.ac.uk/1042/.
(PDF)

S4 Fig DRG filtering as recorded in isoflurane anesthetized rats. The recordings similar to that shown in Fig 1 but performed in male rats anesthetized with isoflurane. (**A**) After baseline was recorded (control), Capsaicin (CAP, 10 μM, 50 μl) was injected into the hindpaw. Application of GABA (200 μM, 3 μl) to DRG reduced CAP-induced firing frequency in DR but not SN (bottom traces). (**B**) Summary for panel A. Two-factor (nerve site, drug application) repeated measures ANOVA: main effects associated with nerve site [F(1,20) = 25.5; *p* < 0.001] and drug application [F(2,19) = 11.8; *p* < 0.01]; significant interaction between nerve site and drug application [F(2,19) = 9.4; *p* < 0.01]. Bonferroni post hoc test: **,***significant difference from control (*p* < 0.01, *p* < 0.001); ^#^significant difference from CAP (*p* < 0.05). (**C**) GABA_A_ antagonist bicuculline (BIC, 200 μM, 3 μl) was applied to DRG; hindpaw was not stimulated. (**D**) Summary for panel C. Two-factor repeated measures ANOVA: main effects associated with nerve site [F(1,22) = 17.4; *p* < 0.01], drug application [F(1,22) = 21.4; *p* < 0.001], significant interaction between nerve site and drug application [F(1,22) = 13.6; *p* < 0.01]. Bonferroni post hoc test: ***significant difference from control (*p* < 0.001). Metadata for quantifications presented in this figure can be found at https://archive.researchdata.leeds.ac.uk/1042/.
(PDF)

S5 Fig Additional spike analyses. (**A**) Top: the latency between SN and DR spikes was calculated; darker colors represent shorter latencies and hotter colors represent longer latencies. Bottom: each spike in the dorsal root was paired with a spike in the spinal nerve based on the minimum latency within a short time window. The end of the time window was defined by an estimation of the slowest conduction velocity of C-fibers; matching temporally uncorrelated spikes during gaps in spike activity was avoided. Using this method, the minimum latency defined the spinal nerve origin of a dorsal root spike. (**B**) The accuracy of latency-based spike matching at different firing frequencies in simulated Poisson-generated spike trains. Filtering was modeled by random deletion of a percentage of DR spikes. Spikes were randomly assigned velocities that were varied around mean A and C fiber velocities. Spike matching accuracy was inversely proportional to firing frequency and was highly dependent on the velocity of the slowest fiber type, C fiber. (**C**) False positive rates for fiber types at different simulated spike frequencies. The false positive rate was relatively low across fiber types but faster conducting fiber types were more likely to have a higher FPR at higher spike train frequencies. Metadata for quantifications presented in this figure can be found at https://archive.researchdata.leeds.ac.uk/1042/. Code for spike sorting analysis is available at GitHub (https://github.com/pnm4sfix/SpikePropagation).
(PDF)

S6 Fig Additional spike analyses. (**A**) Raster plot for each clustered waveform (denoted as Unit) under control, CAP and CAP+GABA conditions after matching the DR spikes with these in the SN. (**B**) Individual waveforms of spike-sorted units (clusters) in the SN. (**C**) Average waveforms of spike-sorted units (clusters) in the SN. (**D**) Average waveforms of matched units in the DR. Metadata for quantifications presented in this figure can be found at ttps://archive.researchdata.leeds.ac.uk/1042/. Code for spike sorting analysis is available at GitHub (https://github.com/pnm4sfix/SpikePropagation).
(PDF)

S7 Fig Additional spike analyses. (**A**) To rule out contamination of dorsal root units with synchronized firing of another fiber, the mean deviation (represented as a z score) of each waveform in the unit from the mean waveform of the unit was calculated. Any spikes originating from another fiber firing in a temporally correlated way should exhibit a different waveform shape and thus be recognized as an outlier (>3 z score). The histogram shows a large majority of spikes were within a z score of 3 from the unit means. (**B**) Mean latencies of the DR spikes matched to the units 1 to 10 from the dataset shown in the S6 Fig. (**C**) Instantaneous firing frequency of all spike-sorted units in the SN and DR. Metadata for quantifications presented in this figure can be found at https://archive.researchdata.leeds.ac.uk/1042/. Code for spike sorting analysis is available at GitHub (https://github.com/pnm4sfix/SpikePropagation).
(PDF)

S8 Fig Transplantation of MGE cells into DRG accelerated the recovery from chronic hyperalgesia. (**A**, **B**) Mechanical (A) and thermal (B) hyperalgesia caused by hindpaw injection of CFA 2 weeks after MGE cells transplantation into L4 DRG of mice. Mechanical sensitivity was measured using the von Frey method and thermal sensitivity was measured using the Hargreaves method (see Materials and methods). Black symbols denote control mice DRG-injected with saline; red symbols denote MGE-transplanted mice. BL1: baseline before transplantation; BL2: baseline after transplantation; CFA: 1 day after the plantar injection of CFA. Number of experiments (n) is indicated as in each panel (1 animal per experiment). (**C**, **D**) experiments similar to A and B, but chronic constriction injury neuropathic pain model was performed instead of CFA injection. All labeling is similar to panels A and B. A: Two-factor (MGE vs. vehicle, time after CFA) repeated measures ANOVA: main effects associated with transplantation [F(1,15) = 22.0; *p* < 0.01] and time after CFA F(4,12) = 2.4; *p* = 0.24]; significant interaction between these factors [F(4,12) = 0.4; *p* = 0.83]. Bonferroni post hoc test: *significant difference between groups at a given time point (*p* < 0.05). B: Two-factor (MGE vs. vehicle, time after CFA) repeated measures ANOVA: main effects associated with transplantation [F(1,15) = 33.2; *p* < 0.001] and time after CFA [F(4,12 = 0.6; *p* = 0.69]; significant interaction between these factors [F(4,12) = 28.2; *p* < 0.01]. Bonferroni post hoc test: *,**,*** significant difference between groups at a given time point (*p* < 0.05, *p* < 0.01, *p* < 0.001). C: Two-factor (MGE vs. vehicle, time after CCI) repeated measures ANOVA: main effects associated with transplantation [F(1,17) = 11.7; *p* < 0.01] and time after CFA [F(5,13) = 5.6; *p* = 0.06]; significant interaction between these factors [F(5,13) = 1.1; *p* = 0.49]. Bonferroni post hoc test: *,*** significant difference between groups at a given time point (*p* < 0.05, *p* < 0.001). B: Two-factor (MGE vs. vehicle, time after CCI) repeated measures ANOVA: main effects associated with transplantation [F [1,17] = 55.1; *p* < 0.001] and time after CFA [F(5,13) = 8.5; *p* < 0.05]; significant interaction between these factors [F(5,13) = 1.5; *p* = 0.37]. Bonferroni post hoc test: ** significant difference between groups at a given time point (*p* < 0.01). Metadata for quantifications presented in this figure can be found at https://archive.researchdata.leeds.ac.uk/1042/.
(PDF)

S9 Fig Optogenetic stimulation of the DRG-transplanted MGE cells enhances filtering of spikes triggered by noxious thermal and mechanical stimulation. (**A**) Example of in vivo recording of the SN and DR activity (similar to these shown in Fig 1). Stimulation of hindpaw of the MGE-transplanted mice with hot water (60°C) increased firing frequency in both SN and DR branches of the nerve (onset of the bottom traces, as compared to basal activity shown in the upper traces). Application of 473-nm laser light to DRG acutely reduced heat-induced firing frequency in DR but not SN (bottom traces). (**B**) Summary for panel A. Two-factor (nerve site, treatment) repeated measures ANOVA: main effect associated with treatment [F(2,7) = 10.5; *p* < 0.05]; significant interaction between nerve site and treatment [F(2,7) = 12.8; *p* < 0.05]. Bonferroni post hoc test: *,**significant difference from control (*p* < 0.05, *p* < 0.01); ^##^significant difference from heat (*p* < 0.05). (**C**) Similar to A and B but the hindpaw was stimulated with ice cube. (**D**) Summary for panel C. Two-factor (nerve site, treatment) repeated measures ANOVA: significant interaction between nerve site and treatment [F(2,7) = 47.8; *p* < 0.01]. Bonferroni post hoc test: *,**significant difference from control (*p* < 0.05, *p* < 0.01); ^#^significant difference from ice (*p* < 0.01). (**E**) Similar to A and B but the hindpaw was stimulated with air puff. (**F**) Summary for panel E. Two-factor (nerve site, treatment) repeated measures ANOVA: main effect associated with treatment [F(2,7) = 11.1; *p* < 0.05]. Bonferroni post hoc test: *significant difference from control (*p* < 0.05). (**G**) Similar to A and B but the hindpaw was stimulated with sub-threshold von Frey filament (0.4 g). (**H**) Summary for panel G. Two-factor (nerve site, treatment) repeated measures ANOVA: main effects associated with treatment [F(2,7) = 32.4; *p* < 0.01]. Bonferroni post hoc test: *,**significant difference from control (*p* < 0.05, *p* < 0.01). (**I**) Similar to A and B but the hindpaw was stimulated with a needle prick. (**J**) Summary for panel I. Two-factor (nerve site, treatment) repeated measures ANOVA: main effect associated with treatment [F(2,7) = 235.3; *p* < 0.001]; significant interaction between nerve site and treatment [F(2,7) = 38.1; *p* < 0.01]. Bonferroni post hoc test: *,**significant difference from control (*p* < 0.05, *p* < 0.01); ^##^significant difference from needle (*p* < 0.01). Metadata for quantifications presented in this figure can be found at https://archive.researchdata.leeds.ac.uk/1042/.
(PDF)

S10 Fig Activity induced by thermal and mechanical stimulation of the receptive fields is filtered at the DRG. (**A**) Example of in vivo recording of the SN and DR activity (similar to these shown in Fig 1). Stimulation of hindpaw of the rat with hot water (60°C) increased firing frequency in both SN and DR branches of the nerve (middle traces, as compared to basal activity shown in the upper traces). Application of GABA (200 μM, 3 μl) to DRG reduced heat-induced firing frequency in DR but not SN (bottom traces). (**B**) Summary for panel A. Two-factor (nerve site, treatment) repeated measures ANOVA: main effects associated with nerve site [F(1,10) = 13.4; *p* < 0.05] and treatment [F(2,9) = 8.3; *p* < 0.05]. Bonferroni post hoc test: **significant difference from control (*p* < 0.01); ^##^significant difference from heat (*p* < 0.01). (**C**) Similar to A and B but the hindpaw was stimulated with ice cube. (**D**) Summary for panel C. Two-factor (nerve site, treatment) repeated measures ANOVA: main effects associated with nerve site [F(1,10) = 150.0; *p* < 0.001] and treatment [F(2,9) = 10.4; *p* < 0.05]; significant interaction between nerve site and treatment [F(2,9) = 13.6; *p* < 0.05]. Bonferroni post hoc test: **significant difference from control (*p* < 0.01); ^###^significant difference from ice (*p* < 0.001). (**E**) Similar to A and B but the hindpaw was stimulated with air puff. (**F**) Summary for panel E. Two-factor (nerve site, treatment) repeated measures ANOVA: main effect associated with treatment [F(2,9) = 7.4; *p* < 0.05]. Bonferroni post hoc test: *,**significant difference from control (*p* < 0.05, *p* < 0.01). (**G**) Similar to A and B but the hindpaw was stimulated with sub-threshold von Frey filament (4 g). (**H**) Summary for panel G. Two-factor (nerve site, treatment) repeated measures ANOVA: main effects associated with nerve site [F(1,10) = 15.0; *p* < 0.05] and treatment [F(2,9) = 11.8; *p* < 0.05]. Bonferroni post hoc test: *significant difference from control (*p* < 0.05). (**I**) Similar to A and B but the hindpaw was stimulated with a needle prick. (**J**) Summary for panel I. Two-factor (nerve site, treatment) repeated measures ANOVA: main effect associated with treatment [F(2,4) = 18.0; *p* < 0.01]; significant interaction between nerve site and treatment [F(2,4) = 7.5; *p* < 0.05]. Bonferroni post hoc test: **significant difference from control (*p* < 0.01); ^#^significant difference from needle (*p* < 0.05). Metadata for quantifications presented in this figure can be found at https://archive.researchdata.leeds.ac.uk/1042/.
(PDF)

S11 Fig Additional single-unit recordings. (**A, B**) TTX blocks evoked spikes. Example traces of in vivo single unit recording from the DR aspect of a rat C fiber (A) or A fiber (B); stimulus electrode is placed in the spinal nerve. Parameters of stimulation and conduction velocity are indicated above the trace. TTX (1 μM, 3 μl; C) was injected into the DRG using a microsyringe at time points indicated by the bent arrow. Black circles indicate failed spikes. A fiber spike waveform for the A fiber recording is shown on the extended time scale within the dotted red box. (**C, D**) In the A-type fibers spike propagation through the DRG is not affects by GABA even at higher stimulation frequencies: 50 Hz (C) and 100 Hz (D). (**E**) Pie charts summarizing the percentage of C and A fibers in which TTX (from experiments shown in panels A and B) or GABA (data from the experiments shown in main Fig 5) produced a conduction block. (F) Spike failure rate before and during application of GABA to A fibers when stimulating at 50 and 100 Hz. No significant effects of GABA were found (*n* = 8; nonparametric Kruskal–Wallis ANOVA). Metadata for quantifications presented in this figure can be found at https://archive.researchdata.leeds.ac.uk/1042/.
(PDF)

S12 Fig Additional analysis of initial axon morphology. Light-sheet microscopy of cleared rat DRG; axon lengths (**A**), stem diameter (**B**), and somatic diameter (**C**) of the peripherin- and NF-200-labeled fibers were analyzed per animal (see also Fig 6C–6E). (A) Peripherin: 3 animals (rat 1: 5 DRGs, 36 stems; rat 2: 2 DRGs, 9 stems; rat 3: 3 DRGs, 12 stems). NF200: 3 animals (rat 1: 4 DRGs, 22 stems; rat 2: 1 DRG, 3 stems; rat 3: 2 DRGs, 5 stems); paired *t* test: t(2) = 3, *p* < 0.05. (B) Peripherin: 3 animals (rat 1: 5 DRGs, 36 stems; rat 2: 2 DRGs, 9 stems; rat 3: 3 DRGs, 12 stems). NF200: 3 animals (rat 1: 4 DRGs, 22 stems; rat 2: 1 DRG, 3 stems; rat 3: 2 DRGs, 5 stems); paired *t* test: t(2) = 3, *p* < 0.05. (C) Peripherin: 3 animals (rat 1: 5 DRGs, 150 somas; rat 2: 2 DRGs, 122 somas; rat 3: 3 DRGs, 155 somas). NF200: 3 animals (rat 1: 4 DRGs, 150 somas; rat 2: 1 DRG, 31 somas; rat 3: 2 DRGs, 75 somas); paired *t* test: t(2) = 7, *p* < 0.05. Metadata for quantifications presented in this figure can be found at https://archive.researchdata.leeds.ac.uk/1042/.
(PDF)

S13 Fig Parameter space analysis. (**A**) C-fiber model parameter space analysis. Fraction of available TTX-sensitive Na channels at the T-junction (TJ) in response to steady-state GABA_A_ receptor activation. For a model with GABA_A_ receptors restricted to the soma, varying the diameter of the stem axon (length 75 μm; top left panel) or varying stem axon length (diameter 1.35 μm; top right panel) generally had only modest effects on the fraction of available Na channels (i.e., proportion of non-inactivated Na channels). In contrast, expressing GABA_A_ receptors on the axons, compared with the soma alone, substantially decreased Na channel availability (stem length 75 μm, stem diameter 1.35 μm; bottom left). Connection of the soma and stem to the TJ made no difference when GABA_A_ receptors were expressed across all compartments, (bottom right). (**B**) A-fiber model parameter space analysis. With GABA_A_ receptors restricted to the soma, varying stem axon diameter (stem length 400 μm) or length (stem diameter 2 μm) substantially affected the available fraction of Na channels (top left and right panels). Likewise, if GABA_A_ receptors were expressed both in the soma/axon initial segment and all nodes of Ranvier, rather than just the soma/AIS alone, fractional availability of Na channels was substantially reduced by GABA_A_ receptor activation (bottom left). Connection of the soma and stem axon to the TJ provided for a greater effect of GABA_A_ receptor activation compared to when detached, although this effect was less pronounced with at higher densities of GABA_A_ receptor activation (bottom right panel). Metadata for quantifications presented in this figure can be found at https://archive.researchdata.leeds.ac.uk/1042/. Code can be found at GitHub (https://github.com/dbjaffe67/DRGsims).
(PDF)

S1 Movie Light-sheet microscopy of cleared rat lumbar DRG immunolabeled with NF-200 (red) and peripherin (green).
(MP4)

S2 Movie The simple neurite tracer plugin (FIJI) was used to semi-automate stem axons and branch point tracing through a 3D image volume of a DRG. The plugin uses a search algorithm to trace the fluorescently labeled axons between 2 points. The video shows the example of tracing the stem axon from a peripherin labeled neuron.
(MP4)

## Abbreviations

ACSF: artificial cerebrospinal fluid
AIS: axon initial segment
CCI: chronic constriction injury
CFA: complete Freund’s adjuvant
ChAT: choline acetyltransferase
CNS: central nervous system
DR: dorsal root
DRG: dorsal root ganglion
FPR: false positive rate
MGE: medial ganglionic eminence
PPN: preganglionic parasympathetic neuron
SN: spinal nerve
VR: ventral root
