# Supplementary material for: Dorsal root ganglia control nociceptive input to the central nervous system
Source: PLoS Biol. 2023 Jan 5;21(1):e3001958. doi: 10.1371/journal.pbio.3001958 (PMC9847955; doi:10.1371/journal.pbio.3001958)
Supplement: S1 Fig — (A) VR transection (schematized in the panel J) does not affect spontaneous activity in the SN or DR. (B) Summary of experiments exemplified in A. Two-factor (nerve site, VR transection) repeated measures ANOVA: main effect associated with nerve site [F(1,12) = 12.7; p < 0.05]. (C) After the VR transection, a baseline was recorded (control) and Capsaicin (CAP, 10 μM, 50 μl) was injected into the hindpaw. CAP increased firing frequency in both SN and DR branches of the nerve (middle traces, as compared to basal activity shown in the upper traces). Application of GABA (200 μM, 3 μl) to DRG reduced CAP-induced firing frequency in DR but not SN (bottom traces). (D) Summary of the panel C. Two-factor (nerve site, drug application) repeated measures ANOVA: main effects associated with nerve site [F(1,10) = 12.2; p < 0.05] and drug application [F(2,9) = 6.5; p < 0.05]; significant interaction between nerve site and drug application [F(2,9) = 41.3; p < 0.01]. Bonferroni post hoc test: **,***significant difference from control (p < 0.01, p < 0.001); ##significant difference from CAP (p < 0.01). (E) After the VR transection, a baseline was recorded (control) and GABAA antagonist bicuculline (BIC, 200 μM, 3 μl) was applied to DRG; hindpaw was not stimulated. (F) Summary for panel E. Two-factor repeated measures ANOVA: main effects associated with nerve site [F(1,12) = 8.2; p < 0.05], drug application [F(1,12) = 18.2; p < 0.01], significant interaction between nerve site and drug application [F(1,12) = 8.1; p < 0.05]. Bonferroni post hoc test: **significant difference from control (p < 0.01). (G) Experiments similar to these shown in (E, F) but GABA was applied instead of BIC. (H) Summary for panel G. Two-factor repeated measures ANOVA: main effect associated with nerve site [F(1,10) = 11.9; p < 0.05). (I) Scatter plot comparison of the firing basal (tonic) firing rates in paired SN-DR recordings with VR intact (left, data from the Fig 1; paired t test: t(25) = 7.2, p < 0.001) [file pbio.3001958.s001.pdf]

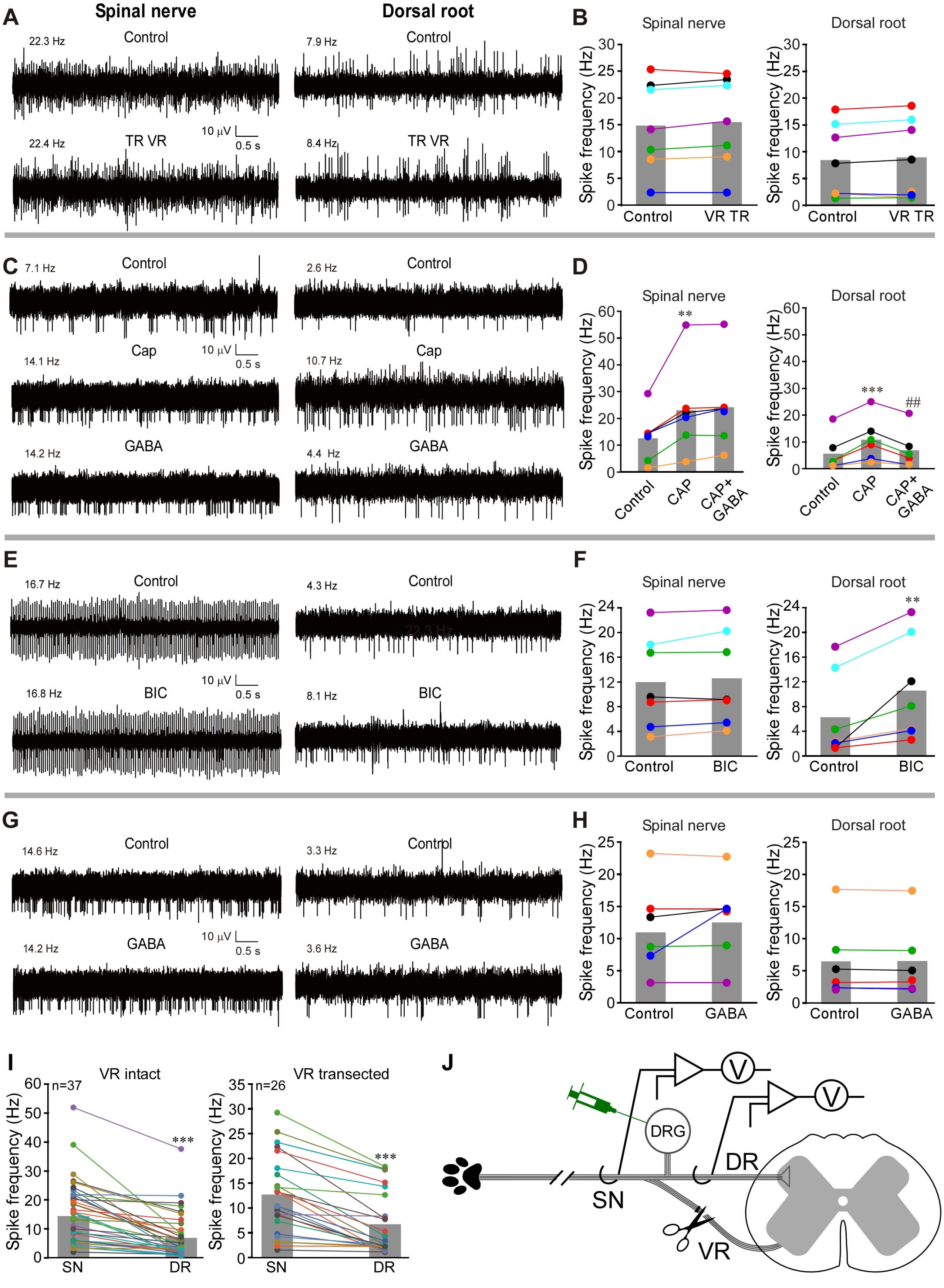

A
Spinal nerve
Dorsal root
22.3 Hz
Control
7.9 Hz
Control
22.4 Hz
TR VR
10 μV
0.5 s
8.4 Hz
TR VR
B
Spinal nerve
Dorsal root
Spike frequency (Hz)
Control
VR TR
C
7.1 Hz
Control
2.6 Hz
Control
14.1 Hz
Cap
10.7 Hz
Cap
14.2 Hz
GABA
4.4 Hz
GABA
D
Spinal nerve
Dorsal root
**
***
##
Control
CAP
CAP+ GABA
E
16.7 Hz
Control
4.3 Hz
Control
16.8 Hz
BIC
8.1 Hz
BIC
F
Spinal nerve
Dorsal root
Control
BIC
G
14.6 Hz
Control
3.3 Hz
Control
14.2 Hz
GABA
3.6 Hz
GABA
H
Spinal nerve
Dorsal root
Control
GABA
I
n=37
VR intact
n=26
VR transected
SN
DR
J
V
DRG
DR
SN
VR

S1 Fig. Ventral root transection does not affect filtering at the DRG. (**A**) VR transection (schematized in the panel J) does not affect spontaneous activity in the SN or DR. (**B**) Summary of experiments exemplified in A. Two-factor (nerve site, VR transection) repeated measures ANOVA: main effect associated with nerve site [$F(1,12)=12.7$; $p<0.05$). (**C**) After the VR transection a baseline was recorded (control) and Capsaicin (CAP, 10 μM, 50 μl) was injected into the hindpaw. CAP increased firing frequency in both SN and DR branches of the nerve (middle traces, as compared to basal activity shown in the upper traces). Application of GABA (200 μM, 3 μl) to DRG reduced CAP-induced firing frequency in DR but not SN (bottom traces). (**D**) Summary of the panel C. Two-factor (nerve site, drug application) repeated measures ANOVA: main effects associated with nerve site [$F(1,10)=12.2$; $p<0.05$] and drug application [$F(2,9)=6.5$; $p<0.05$]; significant interaction between nerve site and drug application [$F(2,9)=41.3$; $p<0.01$]. Bonferroni post-hoc test: **,***significant difference from control ($p<0.01$, $p<0.001$); ##significant difference from CAP ($p<0.01$). (**E**) After the VR transection a baseline was recorded (control) and $GABA_A$ antagonist bicuculline (BIC, 200 μM, 3 μl) was applied to DRG; hindpaw was not stimulated. (**F**) Summary for panel E. Two-factor repeated measures ANOVA: main effects associated with nerve site [$F(1,12)=8.2$; $p<0.05$), drug application [$F(1,12)=18.2$; $p<0.01$), significant interaction between nerve site and drug application [$F(1,12)= 8.1$; $p<0.05$]. Bonferroni post-hoc test: **significant difference from control ($p<0.01$). (**G**) Experiments similar to these shown in (E, F) but GABA was applied instead of BIC. (**H**) Summary for panel G. Two-factor repeated measures ANOVA: main effect associated with nerve site [$F(1,10)=11.9$; $p<0.05$). (**I**) Scatter plot comparison of the firing basal (tonic) firing rates in paired SN-DR recordings with VR intact (left, data from the Fig. 1; Paired t-test: $t(25)=7.2$, $p<0.001$) and VR transected (right; Paired t-test: $t(36)=6.8$, $p<0.001$). (**J**) Schematic of the preparation. The overall procedure is similar to that in Fig.1 but ventral root (VR) is transected before (or during) the recording. Schematics are drawn with Canvas X 2019. Metadata for quantifications presented in this figure can be found at https://archive.researchdata.leeds.ac.uk/1042/
