# Supplementary material for: Dorsal root ganglia control nociceptive input to the central nervous system
Source: PLoS Biol. 2023 Jan 5;21(1):e3001958. doi: 10.1371/journal.pbio.3001958 (PMC9847955; doi:10.1371/journal.pbio.3001958)
Supplement: S2 Fig — The ventral root was surgically transected as in S1 Fig. The animals were left to recover and further experiments were done 2 weeks after the transection. (A) Immunostaining of the L5 spinal nerve of control (right) and VR-transected animals for choline acetyltransferase. (B) Two weeks after the VR transection, a baseline was recorded (control) and Capsaicin (CAP, 10 μM, 50 μl) was injected into the hindpaw. CAP increased firing frequency in both SN and DR branches of the nerve (middle traces, as compared to basal activity shown in the upper traces). Application of GABA (200 μM, 3 μl) to DRG reduced CAP-induced firing frequency in DR but not SN (bottom traces). (C) Summary for panel B. Two-factor (nerve site, drug application) repeated measures ANOVA: main effects associated with nerve site [F(1,12) = 15.8; p < 0.01] and drug application [F(1,12) = 34.8; p < 0.01]; significant interaction between nerve site and drug application [F(2,11) = 7.7; p < 0.05]. Bonferroni post hoc test: *,***significant difference from control (p < 0.05, p < 0.001); ##significant difference from CAP (p < 0.01). (D) GABAA antagonist bicuculline (BIC, 200 μM, 3 μl) was applied to DRG; hindpaw was not stimulated. (E) Summary for panel D. Two-factor repeated measures ANOVA: main effects associated with nerve site [F(1,12) = 12.9; p < 0.05], drug application [F(1,12) = 52.9; p < 0.001], significant interaction between nerve site and drug application [F(1,12) = 14.8; p < 0.01]. Bonferroni post hoc test: ***significant difference from control (p < 0.001). Metadata for quantifications presented in this figure can be found at https://archive.researchdata.leeds.ac.uk/1042/. (PDF) [file pbio.3001958.s002.pdf]

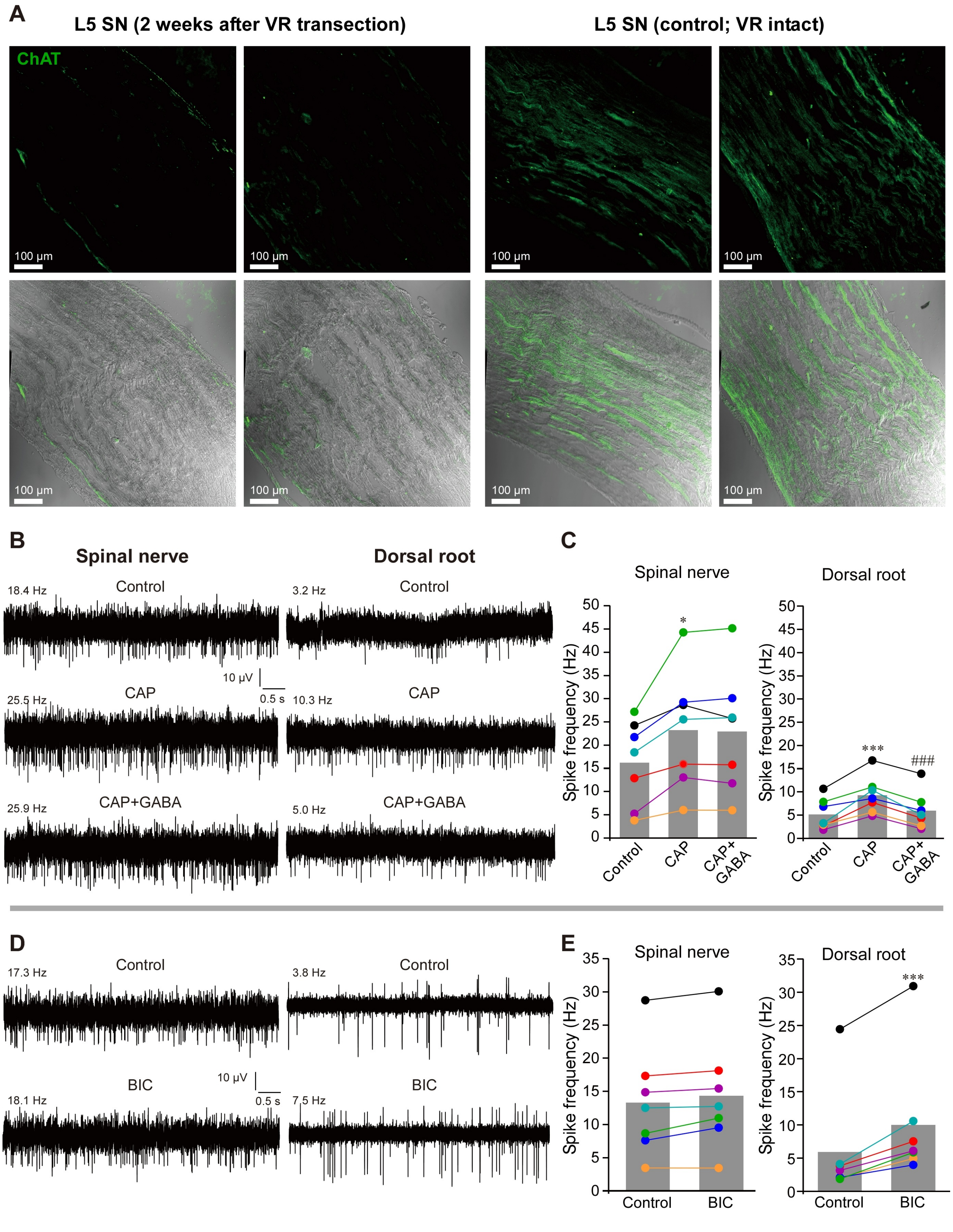
A
L5 SN (2 weeks after VR transection)
L5 SN (control; VR intact)
ChAT
100 μm
B
Spinal nerve
Dorsal root
18.4 Hz
Control
3.2 Hz
Control
10 μV
0.5 s
25.5 Hz
CAP
10.3 Hz
CAP
25.9 Hz
CAP+GABA
5.0 Hz
CAP+GABA
C
Spinal nerve
Dorsal root
Spike frequency (Hz)
*
***
###
Control
CAP
CAP+ GABA
D
17.3 Hz
Control
3.8 Hz
Control
BIC
10 μV
0.5 s
18.1 Hz
7.5 Hz
BIC
E
Spinal nerve
Dorsal root
Spike frequency (Hz)
***
Control
BIC

S2 Fig. Efferent fiber elimination does not affect filtering at the DRG. The ventral root was surgically transected as in Suppl. Fig. 1. The animals were left to recover (see Suppl. Methods) and further experiments were done 2 weeks after the transection. (**A**) Immunostaining of the L5 spinal nerve of control (right) and VR-transected animals for Choline Acetyltransferase (ChAT). (**B**) 2 weeks after the VR transection a baseline was recorded (control) and Capsaicin (CAP, 10 μM, 50 μl) was injected into the hindpaw. CAP increased firing frequency in both SN and DR branches of the nerve (middle traces, as compared to basal activity shown in the upper traces). Application of GABA (200 μM, 3 μl) to DRG reduced CAP-induced firing frequency in DR but not SN (bottom traces). (**C**) Summary for panel B. Two-factor (nerve site, drug application) repeated measures ANOVA: main effects associated with nerve site [$F(1,12)=15.8$; $p<0.01$] and drug application [$F(2,11)=34.8$; $p<0.01$]; significant interaction between nerve site and drug application [$F(2,11)=7.7$; $p<0.05$]. Bonferroni post-hoc test: *,***significant difference from control ($p<0.05$, $p<0.001$); $^{\#\#}$significant difference from CAP ($p<0.01$). (**D**) $GABA_A$ antagonist bicuculline (BIC, 200 μM, 3 μl) was applied to DRG; hindpaw was not stimulated. (**E**) Summary for panel D. Two-factor repeated measures ANOVA: main effects associated with nerve site [$F(1,12)=12.9$; $p<0.05$), drug application [$F(1,12)=52.9$; $p<0.001$), significant interaction between nerve site and drug application [$F(1,12)=14.8$; $p<0.01$]. Bonferroni post-hoc test: ***significant difference from control ($p<0.001$). Metadata for quantifications presented in this figure can be found at https://archive.researchdata.leeds.ac.uk/1042/
