# Supplementary material for: Dorsal root ganglia control nociceptive input to the central nervous system
Source: PLoS Biol. 2023 Jan 5;21(1):e3001958. doi: 10.1371/journal.pbio.3001958 (PMC9847955; doi:10.1371/journal.pbio.3001958)
Supplement: S3 Fig — The recordings similar to that shown in Fig 1 but performed in female rats. (A) After baseline was recorded (control), Capsaicin (CAP, 10 μM, 50 μl) was injected into the hindpaw. Application of GABA (200 μM, 3 μl) to DRG reduced CAP-induced firing frequency in DR but not SN (bottom traces). (B) Summary for panel A. Two-factor (nerve site, drug application) repeated measures ANOVA: main effect associated with nerve site [F(1,10) = 8.7; p < 0.05]. Bonferroni post hoc test: *,**significant difference from control (p < 0.05, p < 0.01); #significant difference from CAP (p < 0.05). (C) GABAA antagonist bicuculline (BIC, 200 μM, 3 μl) was applied to DRG; hindpaw was not stimulated. (D) Summary for panel C. Two-factor repeated measures ANOVA: main effects associated with nerve site [F(1,10) = 10.4; p < 0.05], drug application [F(1,10) = 12.2; p < 0.05], significant interaction between nerve site and drug application [F(1,10) = 23.1; p < 0.01]. Bonferroni post hoc test: **significant difference from control (p < 0.01). Metadata for quantifications presented in this figure can be found at https://archive.researchdata.leeds.ac.uk/1042/. (PDF) [file pbio.3001958.s003.pdf]

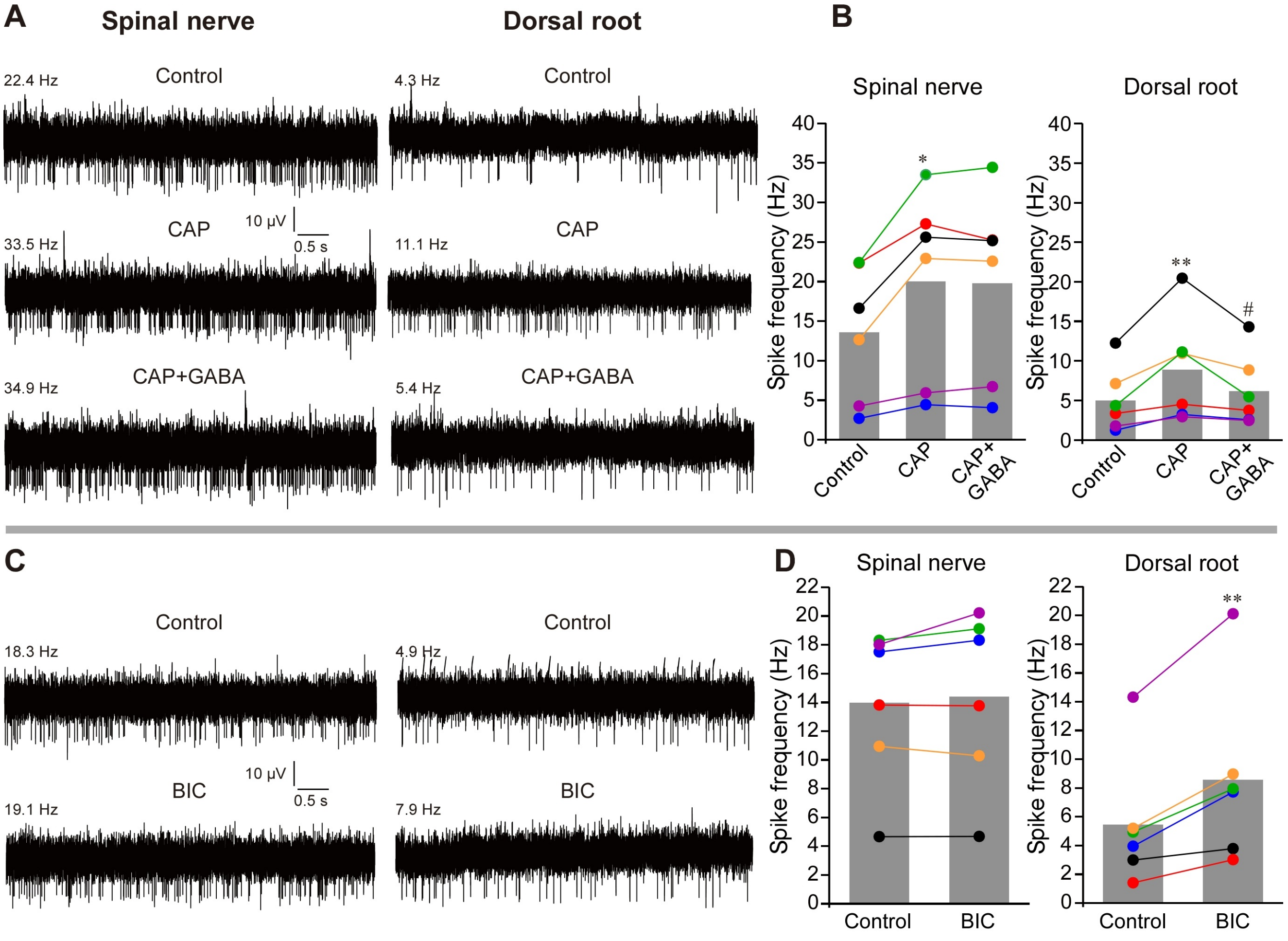

S3 Fig. DRG filtering in the female rats. The recordings similar to that shown in Fig.1 but performed in female rats. (**A**) After baseline was recorded (control), Capsaicin (CAP, 10 µM, 50 µl) was injected into the hindpaw. Application of GABA (200 µM, 3 µl) to DRG reduced CAP-induced firing frequency in DR but not SN (bottom traces). (**B**) Summary for panel A. Two-factor (nerve site, drug application) repeated measures ANOVA: main effect associated with nerve site [F(1,10)=8.7; p<0.05]. Bonferroni post-hoc test: *,**significant difference from control (p<0.05, p<0.01); #significant difference from CAP (p<0.05). (**C**) $GABA_A$ antagonist bicuculline (BIC, 200 µM, 3 µl) was applied to DRG; hindpaw was not stimulated. (**D**) Summary for panel C. Two-factor repeated measures ANOVA: main effects associated with nerve site [F(1,10)=10.4; p<0.05), drug application [F(1,10)=12.2; p<0.05), significant interaction between nerve site and drug application [F(1,10)=23.1; p<0.01]. Bonferroni post-hoc test: **significant difference from control (p<0.01). Metadata for quantifications presented in this figure can be found at https://archive.researchdata.leeds.ac.uk/1042/
