# Supplementary material for: Dorsal root ganglia control nociceptive input to the central nervous system
Source: PLoS Biol. 2023 Jan 5;21(1):e3001958. doi: 10.1371/journal.pbio.3001958 (PMC9847955; doi:10.1371/journal.pbio.3001958)
Supplement: S4 Fig — The recordings similar to that shown in Fig 1 but performed in male rats anesthetized with isoflurane. (A) After baseline was recorded (control), Capsaicin (CAP, 10 μM, 50 μl) was injected into the hindpaw. Application of GABA (200 μM, 3 μl) to DRG reduced CAP-induced firing frequency in DR but not SN (bottom traces). (B) Summary for panel A. Two-factor (nerve site, drug application) repeated measures ANOVA: main effects associated with nerve site [F(1,20) = 25.5; p < 0.001] and drug application [F(2,19) = 11.8; p < 0.01]; significant interaction between nerve site and drug application [F(2,19) = 9.4; p < 0.01]. Bonferroni post hoc test: **,***significant difference from control (p < 0.01, p < 0.001); #significant difference from CAP (p < 0.05). (C) GABAA antagonist bicuculline (BIC, 200 μM, 3 μl) was applied to DRG; hindpaw was not stimulated. (D) Summary for panel C. Two-factor repeated measures ANOVA: main effects associated with nerve site [F(1,22) = 17.4; p < 0.01], drug application [F(1,22) = 21.4; p < 0.001], significant interaction between nerve site and drug application [F(1,22) = 13.6; p < 0.01]. Bonferroni post hoc test: ***significant difference from control (p < 0.001). Metadata for quantifications presented in this figure can be found at https://archive.researchdata.leeds.ac.uk/1042/. (PDF) [file pbio.3001958.s004.pdf]

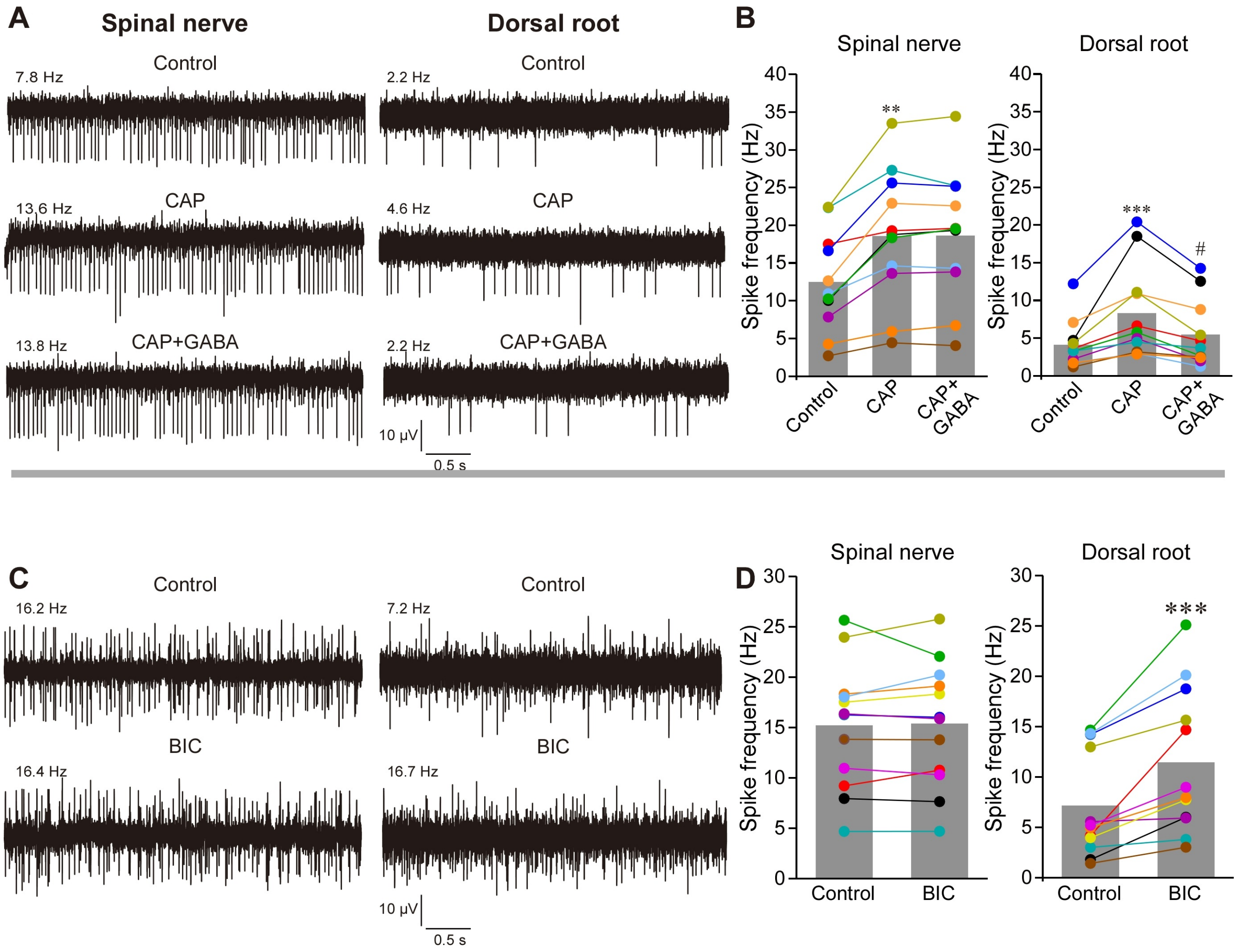

S4 Fig. DRG filtering as recorded in isoflurane anesthetized rats. The recordings similar to that shown in Fig.1 but performed in male rats anesthetized with isoflurane. (**A**) After baseline was recorded (control), Capsaicin (CAP, 10 μM, 50 μl) was injected into the hindpaw. Application of GABA (200 μM, 3 μl) to DRG reduced CAP-induced firing frequency in DR but not SN (bottom traces). (**B**) Summary for panlel A. Two-factor (nerve site, drug application) repeated measures ANOVA: main effects associated with nerve site [$F(1,20)=25.5$; $p<0.001$] and drug application [$F(2,19)=11.8$; $p<0.01$]; significant interaction between nerve site and drug application [$F(2,19)=9.4$; $p<0.01$]. Bonferroni post-hoc test: **,***significant difference from control ($p<0.01$, $p<0.001$); $^{\#}$significant difference from CAP ($p<0.05$). (**C**) $GABA_A$ antagonist bicuculline (BIC, 200 μM, 3 μl) was applied to DRG; hindpaw was not stimulated. (**D**) Summary for panel C. Two-factor repeated measures ANOVA: main effects associated with nerve site [$F(1,22)=17.4$; $p<0.01$), drug application [$F(1,22)=21.4$; $p<0.001$), significant interaction between nerve site and drug application [$F(1,22)=13.6$; $p<0.01$]. Bonferroni post-hoc test: ***significant difference from control ($p<0.001$). Metadata for quantifications presented in this figure can be found at https://archive.researchdata.leeds.ac.uk/1042/
