# Supplementary material for: Dorsal root ganglia control nociceptive input to the central nervous system
Source: PLoS Biol. 2023 Jan 5;21(1):e3001958. doi: 10.1371/journal.pbio.3001958 (PMC9847955; doi:10.1371/journal.pbio.3001958)
Supplement: S5 Fig — (A) Top: the latency between SN and DR spikes was calculated; darker colors represent shorter latencies and hotter colors represent longer latencies. Bottom: each spike in the dorsal root was paired with a spike in the spinal nerve based on the minimum latency within a short time window. The end of the time window was defined by an estimation of the slowest conduction velocity of C-fibers; matching temporally uncorrelated spikes during gaps in spike activity was avoided. Using this method, the minimum latency defined the spinal nerve origin of a dorsal root spike. (B) The accuracy of latency-based spike matching at different firing frequencies in simulated Poisson-generated spike trains. Filtering was modeled by random deletion of a percentage of DR spikes. Spikes were randomly assigned velocities that were varied around mean A and C fiber velocities. Spike matching accuracy was inversely proportional to firing frequency and was highly dependent on the velocity of the slowest fiber type, C fiber. (C) False positive rates for fiber types at different simulated spike frequencies. The false positive rate was relatively low across fiber types but faster conducting fiber types were more likely to have a higher FPR at higher spike train frequencies. Metadata for quantifications presented in this figure can be found at https://archive.researchdata.leeds.ac.uk/1042/. Code for spike sorting analysis is available at GitHub (https://github.com/pnm4sfix/SpikePropagation). (PDF) [file pbio.3001958.s005.pdf]

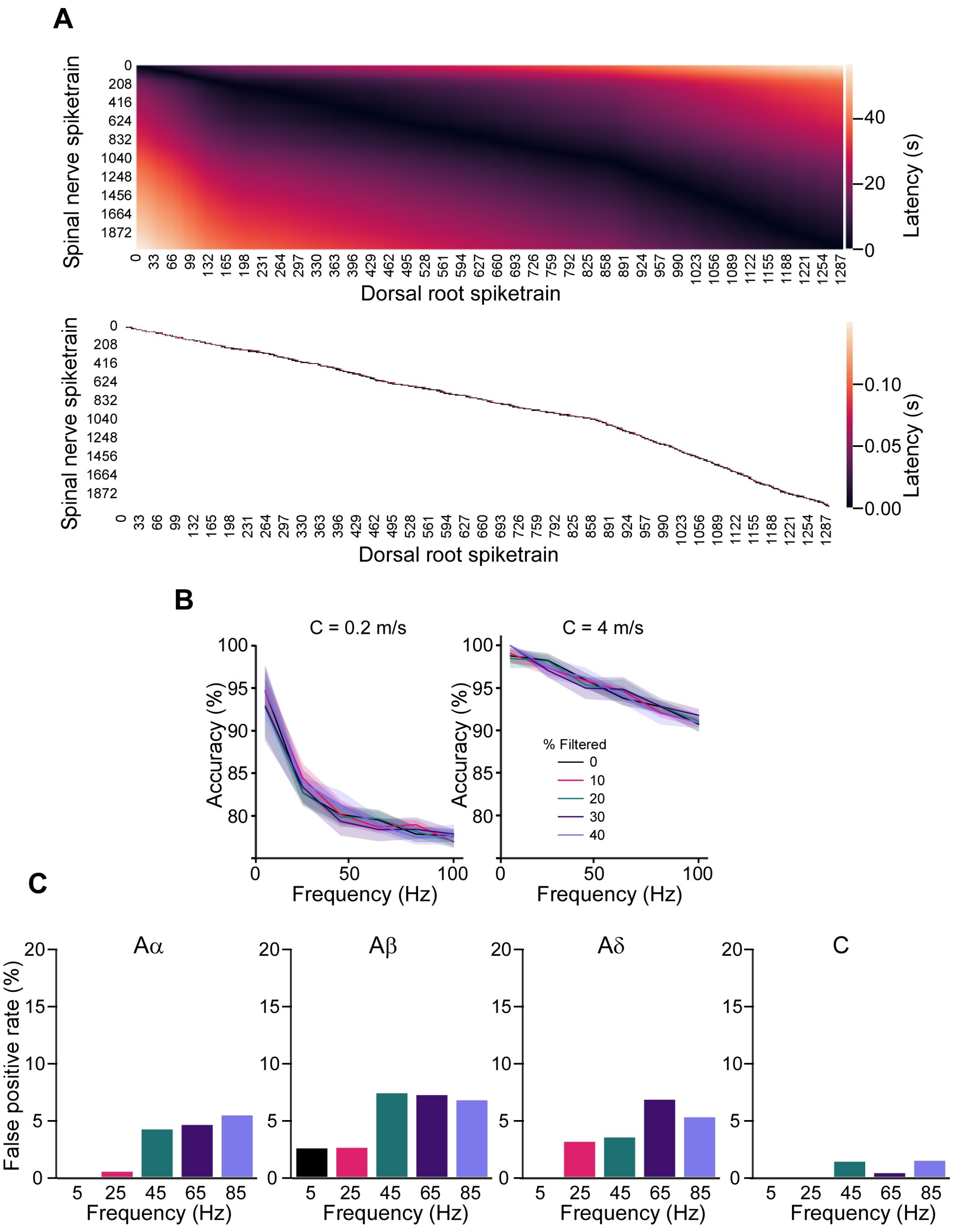
A
Spinal nerve spiketrain
Dorsal root spiketrain
Latency (s)
B
C = 0.2 m/s
C = 4 m/s
Accuracy (%)
Frequency (Hz)
% Filtered
0
10
20
30
40
C
Aα
Aβ
Aδ
C
False positive rate (%)
Frequency (Hz)

S5 Fig. Additional spike analyses. (**A**) Top: the latency between SN and DR spikes was calculated; darker colours represent shorter latencies and hotter colours represent longer latencies. Bottom: each spike in the dorsal root was paired with a spike in the spinal nerve based on the minimum latency within a short time window. The end of the time window was defined by an estimation of the slowest conduction velocity of C-fibres; matching temporally uncorrelated spikes during gaps in spike activity was avoided. Using this method, the minimum latency defined the spinal nerve origin of a dorsal root spike. (**B**) The accuracy of latency-based spike matching at different firing frequencies in simulated poisson-generated spike trains. Filtering was modelled by random deletion of a percentage of DR spikes. Spikes were randomly assigned velocities which were varied around mean A and C fibre velocities. Spike matching accuracy was inversely proportional to firing frequency and was highly dependent on the velocity of the slowest fibre type, C fibre. (**C**) False positive rates (FPR) for fibre types at different simulated spike frequencies. The false positive rate was relatively low across fibre types but faster conducting fibre types were more likely to have a higher FPR at higher spike train frequencies. Metadata for quantifications presented in this figure can be found at https://archive.researchdata.leeds.ac.uk/1042/ code for spike sorting analysis is available at GitHub (https://github.com/pnm4sfix/SpikePropagation).
