# Supplementary material for: Dorsal root ganglia control nociceptive input to the central nervous system
Source: PLoS Biol. 2023 Jan 5;21(1):e3001958. doi: 10.1371/journal.pbio.3001958 (PMC9847955; doi:10.1371/journal.pbio.3001958)
Supplement: S6 Fig — (A) Raster plot for each clustered waveform (denoted as Unit) under control, CAP and CAP+GABA conditions after matching the DR spikes with these in the SN. (B) Individual waveforms of spike-sorted units (clusters) in the SN. (C) Average waveforms of spike-sorted units (clusters) in the SN. (D) Average waveforms of matched units in the DR. Metadata for quantifications presented in this figure can be found at ttps://archive.researchdata.leeds.ac.uk/1042/. Code for spike sorting analysis is available at GitHub (https://github.com/pnm4sfix/SpikePropagation). (PDF) [file pbio.3001958.s006.pdf]

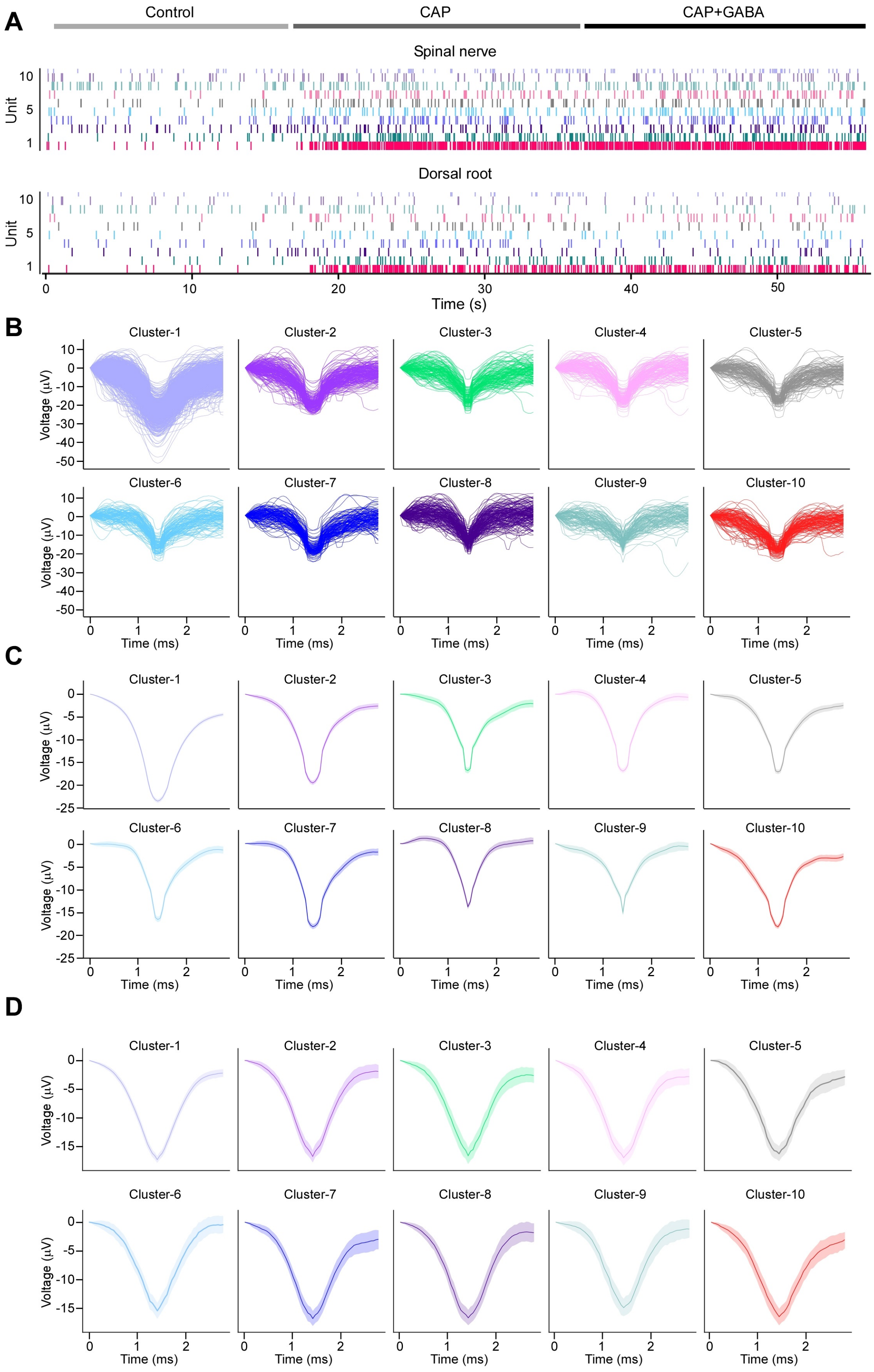
A
Control
CAP
CAP+GABA
Spinal nerve
Unit
Dorsal root
Time (s)
B
Cluster-1
Cluster-2
Cluster-3
Cluster-4
Cluster-5
Cluster-6
Cluster-7
Cluster-8
Cluster-9
Cluster-10
Voltage (μV)
Time (ms)
C
D

S6 Fig. Additional spike analyses. (**A**) Raster plot for each clustered waveform (denoted as Unit) under control, CAP and CAP+GABA conditions after matching the DR spikes with these in the SN. (**B**) Individual waveforms of spike sorted units (cluster) in the SN. (**C**) Average waveforms of spike sorted units (clusters) in the SN. (**D**) Average waveforms of matched units in the DR. Metadata for quantifications presented in this figure can be found at https://archive.researchdata.leeds.ac.uk/1042/ code for spike sorting analysis is available at GitHub (https://github.com/pnm4sfix/SpikePropagation).
