# Supplementary material for: Dorsal root ganglia control nociceptive input to the central nervous system
Source: PLoS Biol. 2023 Jan 5;21(1):e3001958. doi: 10.1371/journal.pbio.3001958 (PMC9847955; doi:10.1371/journal.pbio.3001958)
Supplement: S7 Fig — (A) To rule out contamination of dorsal root units with synchronized firing of another fiber, the mean deviation (represented as a z score) of each waveform in the unit from the mean waveform of the unit was calculated. Any spikes originating from another fiber firing in a temporally correlated way should exhibit a different waveform shape and thus be recognized as an outlier (>3 z score). The histogram shows a large majority of spikes were within a z score of 3 from the unit means. (B) Mean latencies of the DR spikes matched to the units 1 to 10 from the dataset shown in the S6 Fig. (C) Instantaneous firing frequency of all spike-sorted units in the SN and DR. Metadata for quantifications presented in this figure can be found at https://archive.researchdata.leeds.ac.uk/1042/. Code for spike sorting analysis is available at GitHub (https://github.com/pnm4sfix/SpikePropagation). (PDF) [file pbio.3001958.s007.pdf]

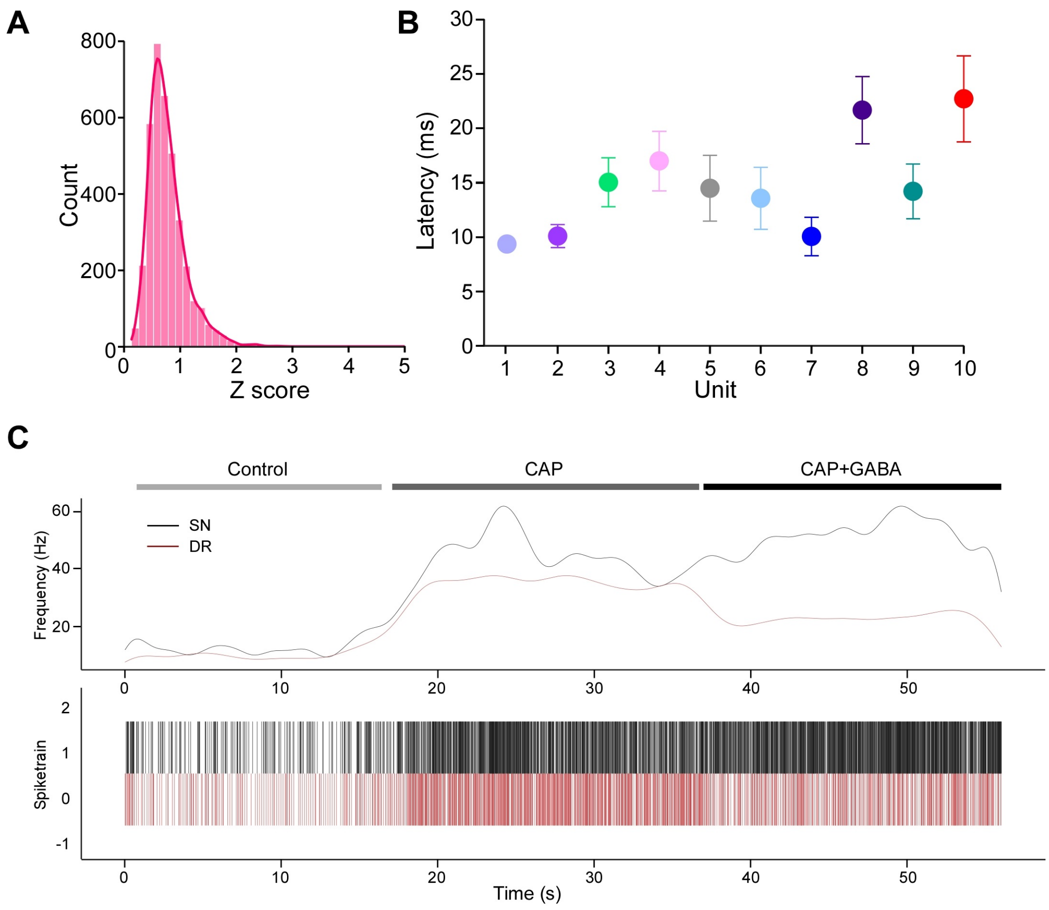

S7 Fig. Additional spike analyses. (**A**) To rule out contamination of dorsal root units with synchronized firing of another fibre, the mean deviation (represented as a z score) of each waveform in the unit from the mean waveform of the unit was calculated. Any spikes originating from another fibre firing in a temporally correlated way should exhibit a different waveform shape and thus be recognized as an outlier (> 3 z score). The histogram shows a large majority of spikes were within a z score of 3 from the unit means. (**B**) Mean latencies of the DR spikes matched to the units 1 to 10 from the dataset shown in the Suppl. Fig. 6. (**C**) Instantaneous firing frequency of all spike sorted units in the SN and DR. Metadata for quantifications presented in this figure can be found at https://archive.researchdata.leeds.ac.uk/1042/ code for spike sorting analysis is available at GitHub (https://github.com/pnm4sfix/SpikePropagation).
