# Supplementary material for: Dorsal root ganglia control nociceptive input to the central nervous system
Source: PLoS Biol. 2023 Jan 5;21(1):e3001958. doi: 10.1371/journal.pbio.3001958 (PMC9847955; doi:10.1371/journal.pbio.3001958)
Supplement: S8 Fig — (A, B) Mechanical (A) and thermal (B) hyperalgesia caused by hindpaw injection of CFA 2 weeks after MGE cells transplantation into L4 DRG of mice. Mechanical sensitivity was measured using the von Frey method and thermal sensitivity was measured using the Hargreaves method (see Materials and methods). Black symbols denote control mice DRG-injected with saline; red symbols denote MGE-transplanted mice. BL1: baseline before transplantation; BL2: baseline after transplantation; CFA: 1 day after the plantar injection of CFA. Number of experiments (n) is indicated as in each panel (1 animal per experiment). (C, D) experiments similar to A and B, but chronic constriction injury neuropathic pain model was performed instead of CFA injection. All labeling is similar to panels A and B. A: Two-factor (MGE vs. vehicle, time after CFA) repeated measures ANOVA: main effects associated with transplantation [F(1,15) = 22.0; p < 0.01] and time after CFA F(4,12) = 2.4; p = 0.24]; significant interaction between these factors [F(4,12) = 0.4; p = 0.83]. Bonferroni post hoc test: *significant difference between groups at a given time point (p < 0.05). B: Two-factor (MGE vs. vehicle, time after CFA) repeated measures ANOVA: main effects associated with transplantation [F(1,15) = 33.2; p < 0.001] and time after CFA [F(4,12 = 0.6; p = 0.69]; significant interaction between these factors [F(4,12) = 28.2; p < 0.01]. Bonferroni post hoc test: *,**,*** significant difference between groups at a given time point (p < 0.05, p < 0.01, p < 0.001). C: Two-factor (MGE vs. vehicle, time after CCI) repeated measures ANOVA: main effects associated with transplantation [F(1,17) = 11.7; p < 0.01] and time after CFA [F(5,13) = 5.6; p = 0.06]; significant interaction between these factors [F(5,13) = 1.1; p = 0.49]. Bonferroni post hoc test: *,*** significant difference between groups at a given time point (p < 0.05, p < 0.001). B: Two-factor (MGE vs. vehicle, time after CCI) repeated measures ANOVA: main e [file pbio.3001958.s008.pdf]

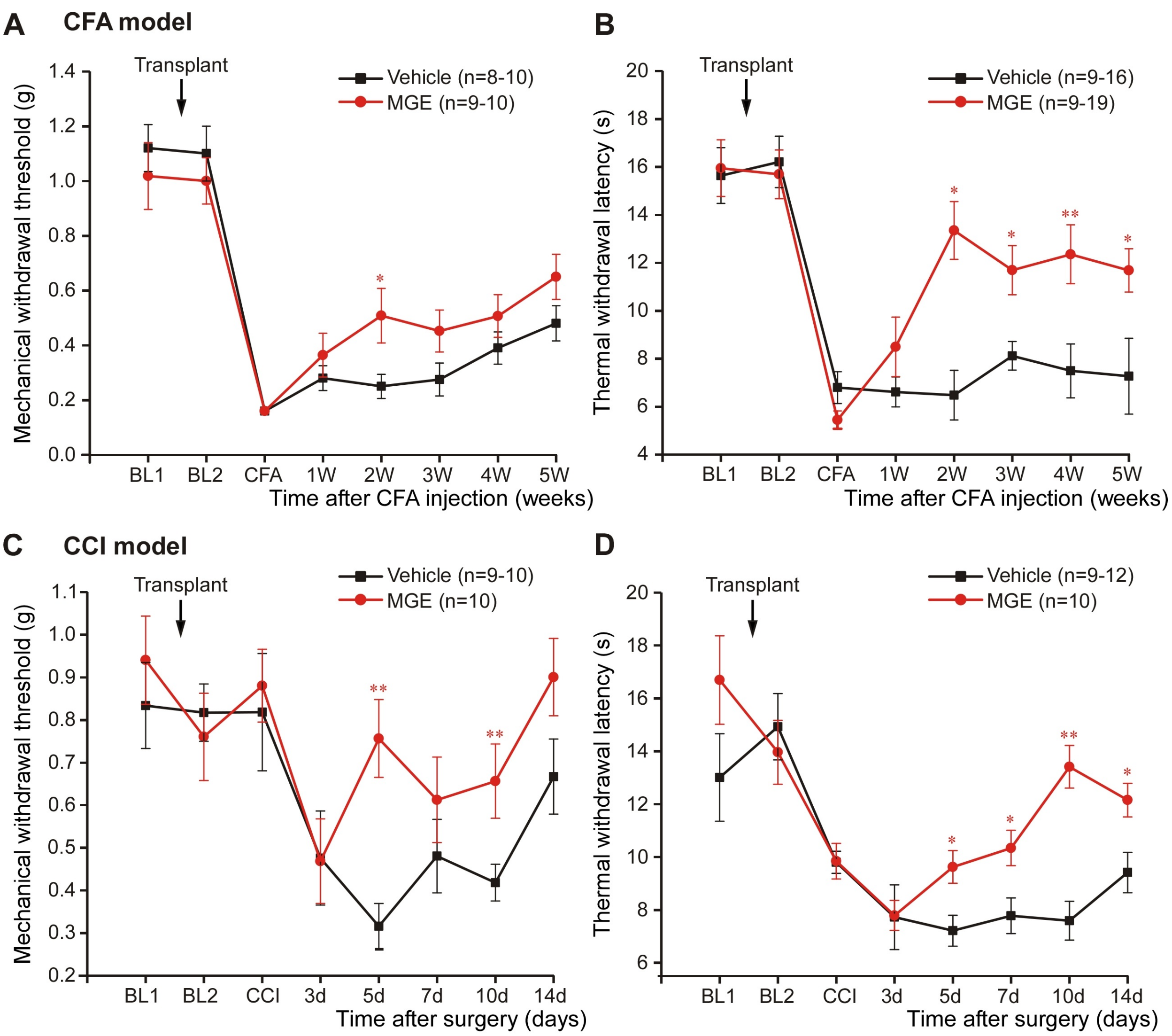

A
CFA model
Transplant
Vehicle (n=8-10)
MGE (n=9-10)
Mechanical withdrawal threshold (g)
1.4
1.2
1.0
0.8
0.6
0.4
0.2
0.0
BL1
BL2
CFA
1W
2W
3W
4W
5W
Time after CFA injection (weeks)
*
B
Transplant
Vehicle (n=9-16)
MGE (n=9-19)
Thermal withdrawal latency (s)
20
18
16
14
12
10
8
6
4
BL1
BL2
CFA
1W
2W
3W
4W
5W
Time after CFA injection (weeks)
*
*
**
*
C
CCI model
Transplant
Vehicle (n=9-10)
MGE (n=10)
Mechanical withdrawal threshold (g)
1.1
1.0
0.9
0.8
0.7
0.6
0.5
0.4
0.3
0.2
BL1
BL2
CCI
3d
5d
7d
10d
14d
Time after surgery (days)
**
**
D
Transplant
Vehicle (n=9-12)
MGE (n=10)
Thermal withdrawal latency (s)
20
18
16
14
12
10
8
6
BL1
BL2
CCI
3d
5d
7d
10d
14d
Time after surgery (days)
*
*
**
*

S8 Fig. Transplantation of MGE cells into DRG accelerated the recovery from chronic hyperalgesia. (**A**, **B**) Mechanical (A) and thermal (B) hyperalgesia caused by hindpaw injection of CFA 2 weeks after MGE cells transplantation into L4 DRG of mice. Mechanical sensitivity was measured using the von Frey method and thermal sensitivity was measured using the Hargreaves method (see Methods). Black symbols denote control mice DRG-injected with saline; red symbols denote MGE-transplanted mice. BL1: baseline before transplantation; BL2: baseline after transplantation; CFA: 1 day after the plantar injection of CFA. Number of experiments (n) is indicated as in each panel (one animal per experiment). (**C**, **D**) experiments similar to A and B, but chronic constriction injury (CCI) neuropathic pain model was performed instead of CFA injection. All labelling is similar to panels A and B. A: Two-factor (MGE vs. vehicle, time after CFA) repeated measures ANOVA: main effects associated with transplantation [$F(1,15)=22.0$; $p<0.01$] and time after CFA [$F(4,12)=2.4$; $p=0.24$]; significant interaction between these factors [$F(4,12)=0.4$; $p=0.83$]. Bonferroni post-hoc test: *significant difference between groups at a given time point ($p<0.05$). B: Two-factor (MGE vs. vehicle, time after CFA) repeated measures ANOVA: main effects associated with transplantation [$F(1,15)=33.2$; $p<0.001$] and time after CFA [$F(4,12)=0.6$; $p=0.69$]; significant interaction between these factors [$F(4,12)=28.2$; $p<0.01$]. Bonferroni post-hoc test: *,**,*** significant difference between groups at a given time point ($p<0.05$, $p<0.01$, $p<0.001$). C: Two-factor (MGE vs. vehicle, time after CCI) repeated measures ANOVA: main effects associated with transplantation [$F(1,17)=11.7$; $p<0.01$] and time after CFA [$F(5,13)=5.6$; $p=0.06$]; significant interaction between these factors [$F(5,13)=1.1$; $p=0.49$]. Bonferroni post-hoc test: *,*** significant difference between groups at a given time point ($p<0.05$, $p<0.001$). B: Two-factor (MGE vs. vehicle, time after CCI) repeated measures ANOVA: main effects associated with transplantation [$F(1,17)=55.1$; $p<0.001$] and time after CFA [$F(5,13)=8.5$; $p<0.05$]; significant interaction between these factors [$F(5,13)=1.5$; $p=0.37$]. Bonferroni post-hoc test: ** significant difference between groups at a given time point ($p<0.01$). Metadata for quantifications presented in this figure can be found at https://archive.researchdata.leeds.ac.uk/1042/
