# Supplementary material for: Dorsal root ganglia control nociceptive input to the central nervous system
Source: PLoS Biol. 2023 Jan 5;21(1):e3001958. doi: 10.1371/journal.pbio.3001958 (PMC9847955; doi:10.1371/journal.pbio.3001958)
Supplement: S9 Fig — (A) Example of in vivo recording of the SN and DR activity (similar to these shown in Fig 1). Stimulation of hindpaw of the MGE-transplanted mice with hot water (60°C) increased firing frequency in both SN and DR branches of the nerve (onset of the bottom traces, as compared to basal activity shown in the upper traces). Application of 473-nm laser light to DRG acutely reduced heat-induced firing frequency in DR but not SN (bottom traces). (B) Summary for panel A. Two-factor (nerve site, treatment) repeated measures ANOVA: main effect associated with treatment [F(2,7) = 10.5; p < 0.05]; significant interaction between nerve site and treatment [F(2,7) = 12.8; p < 0.05]. Bonferroni post hoc test: *,**significant difference from control (p < 0.05, p < 0.01); ##significant difference from heat (p < 0.05). (C) Similar to A and B but the hindpaw was stimulated with ice cube. (D) Summary for panel C. Two-factor (nerve site, treatment) repeated measures ANOVA: significant interaction between nerve site and treatment [F(2,7) = 47.8; p < 0.01]. Bonferroni post hoc test: *,**significant difference from control (p < 0.05, p < 0.01); #significant difference from ice (p < 0.01). (E) Similar to A and B but the hindpaw was stimulated with air puff. (F) Summary for panel E. Two-factor (nerve site, treatment) repeated measures ANOVA: main effect associated with treatment [F(2,7) = 11.1; p < 0.05]. Bonferroni post hoc test: *significant difference from control (p < 0.05). (G) Similar to A and B but the hindpaw was stimulated with sub-threshold von Frey filament (0.4 g). (H) Summary for panel G. Two-factor (nerve site, treatment) repeated measures ANOVA: main effects associated with treatment [F(2,7) = 32.4; p < 0.01]. Bonferroni post hoc test: *,**significant difference from control (p < 0.05, p < 0.01). (I) Similar to A and B but the hindpaw was stimulated with a needle prick. (J) Summary for panel I. Two-factor (nerve site, treatment) repeated measures ANOVA: main effect associated w [file pbio.3001958.s009.pdf]

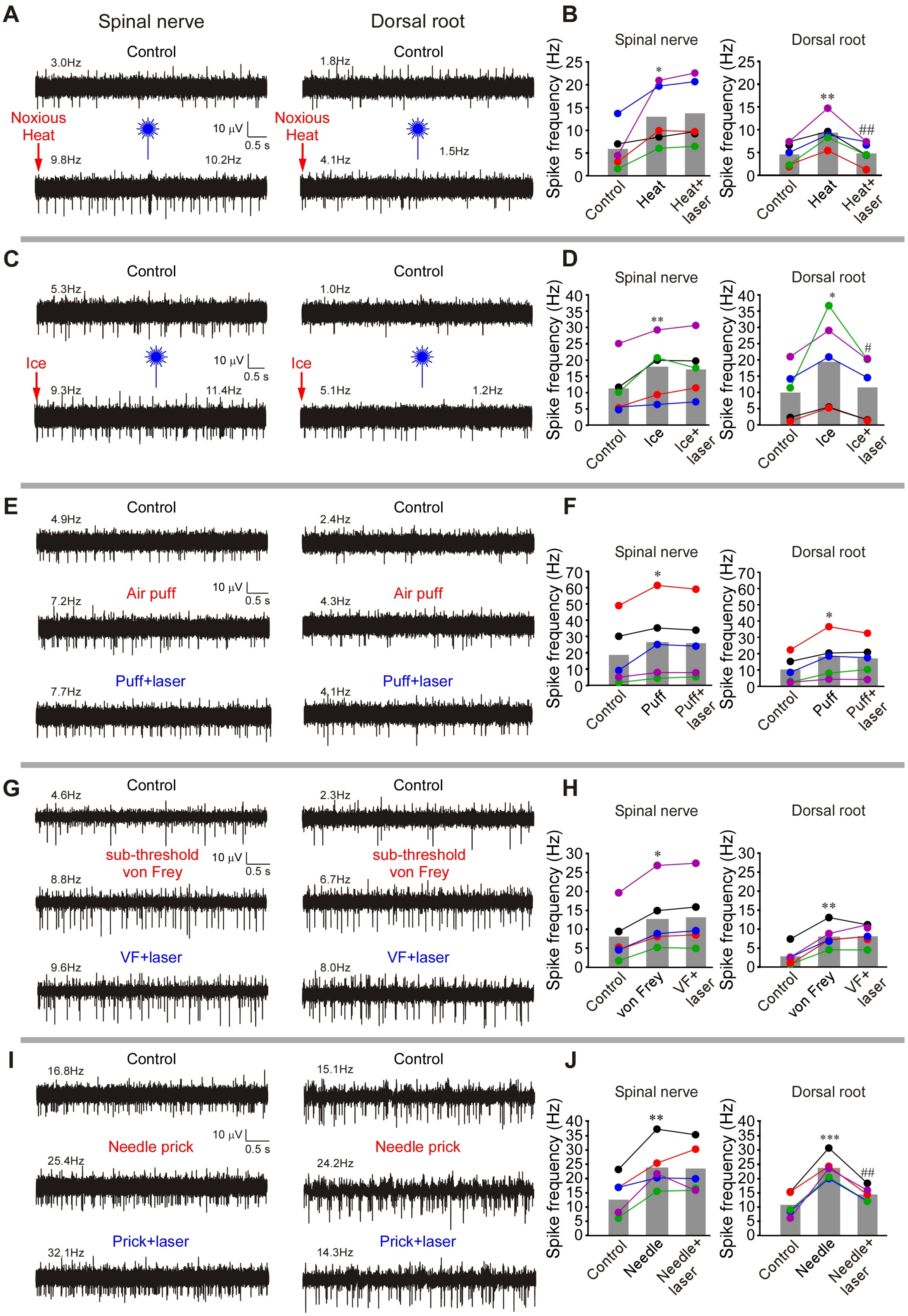

A
Spinal nerve
Dorsal root
Control
3.0Hz
1.8Hz
Noxious Heat
9.8Hz
10.2Hz
4.1Hz
1.5Hz
10 μV
0.5 s
B
Spinal nerve
Dorsal root
Spike frequency (Hz)
Control
Heat
Heat+ laser
C
5.3Hz
1.0Hz
Ice
9.3Hz
11.4Hz
5.1Hz
1.2Hz
D
Ice+ laser
E
4.9Hz
2.4Hz
Air puff
7.2Hz
4.3Hz
Puff+laser
7.7Hz
4.1Hz
F
Puff
Puff+ laser
G
4.6Hz
2.3Hz
sub-threshold von Frey
8.8Hz
6.7Hz
VF+laser
9.6Hz
8.0Hz
H
von Frey
VF+ laser
I
16.8Hz
15.1Hz
Needle prick
25.4Hz
24.2Hz
Prick+laser
32.1Hz
14.3Hz
J
Needle
Needle+ laser

S9 Fig. Optogenetic stimulation of the DRG-transplanted MGE cells enhances filtering of spikes triggered by noxious thermal and mechanical stimulation. (**A**) Example of *in vivo* recording of the SN and DR activity (similar to these shown in Fig. 1). Stimulation of hindpaw of the MGE-transplanted mice with hot water (60°C) increased firing frequency in both SN and DR branches of the nerve (onset of the bottom traces, as compared to basal activity shown in the upper traces). Application of 473 nm laser light to DRG acutely reduced heat-induced firing frequency in DR but not SN (bottom traces). (**B**) Summary for panel A. Two-factor (nerve site, treatment) repeated measures ANOVA: main effect associated with treatment [$F(2,7)=10.5$; $p<0.05$]; significant interaction between nerve site and treatment [$F(2,7)=12.8$; $p<0.05$]. Bonferroni post-hoc test: *,**significant difference from control ($p<0.05$, $p<0.01$); ##significant difference from heat ($p<0.05$). (**C**) Similar to A and B but the hindpaw was stimulated with ice cube. (**D**) Summary for panel C. Two-factor (nerve site, treatment) repeated measures ANOVA: significant interaction between nerve site and treatment [$F(2,7)=47.8$; $p<0.01$]. Bonferroni post-hoc test: *,**significant difference from control ($p<0.05$, $p<0.01$); #significant difference from ice ($p<0.01$). (**E**) Similar to A and B but the hindpaw was stimulated with air puff. (**F**) Summary for panel E. Two-factor (nerve site, treatment) repeated measures ANOVA: main effect associated with treatment [$F(2,7)=11.1$; $p<0.05$]. Bonferroni post-hoc test: *significant difference from control ($p<0.05$). (**G**) Similar to A and B but the hindpaw was stimulated with sub-threshold von Frey filament (0.4g). (**H**) Summary for panel G. Two-factor (nerve site, treatment) repeated measures ANOVA: main effects associated with treatment [$F(2,7)=32.4$; $p<0.01$]. Bonferroni post-hoc test: *,**significant difference from control ($p<0.05$, $p<0.01$). (**I**) Similar to A and B but the hindpaw was stimulated with a needle prick. (**J**) Summary for panel I. Two-factor (nerve site, treatment) repeated measures ANOVA: main effect associated with treatment [$F(2,7)=235.3$; $p<0.001$]; significant interaction between nerve site and treatment [$F(2,7)=38.1$; $p<0.01$]. Bonferroni post-hoc test: *,**significant difference from control ($p<0.05$, $p<0.01$); ##significant difference from needle ($p<0.01$). Metadata for quantifications presented in this figure can be found at https://archive.researchdata.leeds.ac.uk/1042/
