# Supplementary material for: Dorsal root ganglia control nociceptive input to the central nervous system
Source: PLoS Biol. 2023 Jan 5;21(1):e3001958. doi: 10.1371/journal.pbio.3001958 (PMC9847955; doi:10.1371/journal.pbio.3001958)
Supplement: S10 Fig — (A) Example of in vivo recording of the SN and DR activity (similar to these shown in Fig 1). Stimulation of hindpaw of the rat with hot water (60°C) increased firing frequency in both SN and DR branches of the nerve (middle traces, as compared to basal activity shown in the upper traces). Application of GABA (200 μM, 3 μl) to DRG reduced heat-induced firing frequency in DR but not SN (bottom traces). (B) Summary for panel A. Two-factor (nerve site, treatment) repeated measures ANOVA: main effects associated with nerve site [F(1,10) = 13.4; p < 0.05] and treatment [F(2,9) = 8.3; p < 0.05]. Bonferroni post hoc test: **significant difference from control (p < 0.01); ##significant difference from heat (p < 0.01). (C) Similar to A and B but the hindpaw was stimulated with ice cube. (D) Summary for panel C. Two-factor (nerve site, treatment) repeated measures ANOVA: main effects associated with nerve site [F(1,10) = 150.0; p < 0.001] and treatment [F(2,9) = 10.4; p < 0.05]; significant interaction between nerve site and treatment [F(2,9) = 13.6; p < 0.05]. Bonferroni post hoc test: **significant difference from control (p < 0.01); ###significant difference from ice (p < 0.001). (E) Similar to A and B but the hindpaw was stimulated with air puff. (F) Summary for panel E. Two-factor (nerve site, treatment) repeated measures ANOVA: main effect associated with treatment [F(2,9) = 7.4; p < 0.05]. Bonferroni post hoc test: *,**significant difference from control (p < 0.05, p < 0.01). (G) Similar to A and B but the hindpaw was stimulated with sub-threshold von Frey filament (4 g). (H) Summary for panel G. Two-factor (nerve site, treatment) repeated measures ANOVA: main effects associated with nerve site [F(1,10) = 15.0; p < 0.05] and treatment [F(2,9) = 11.8; p < 0.05]. Bonferroni post hoc test: *significant difference from control (p < 0.05). (I) Similar to A and B but the hindpaw was stimulated with a needle prick. (J) Summary for panel I. Two-factor (nerve site, treatment) r [file pbio.3001958.s010.pdf]

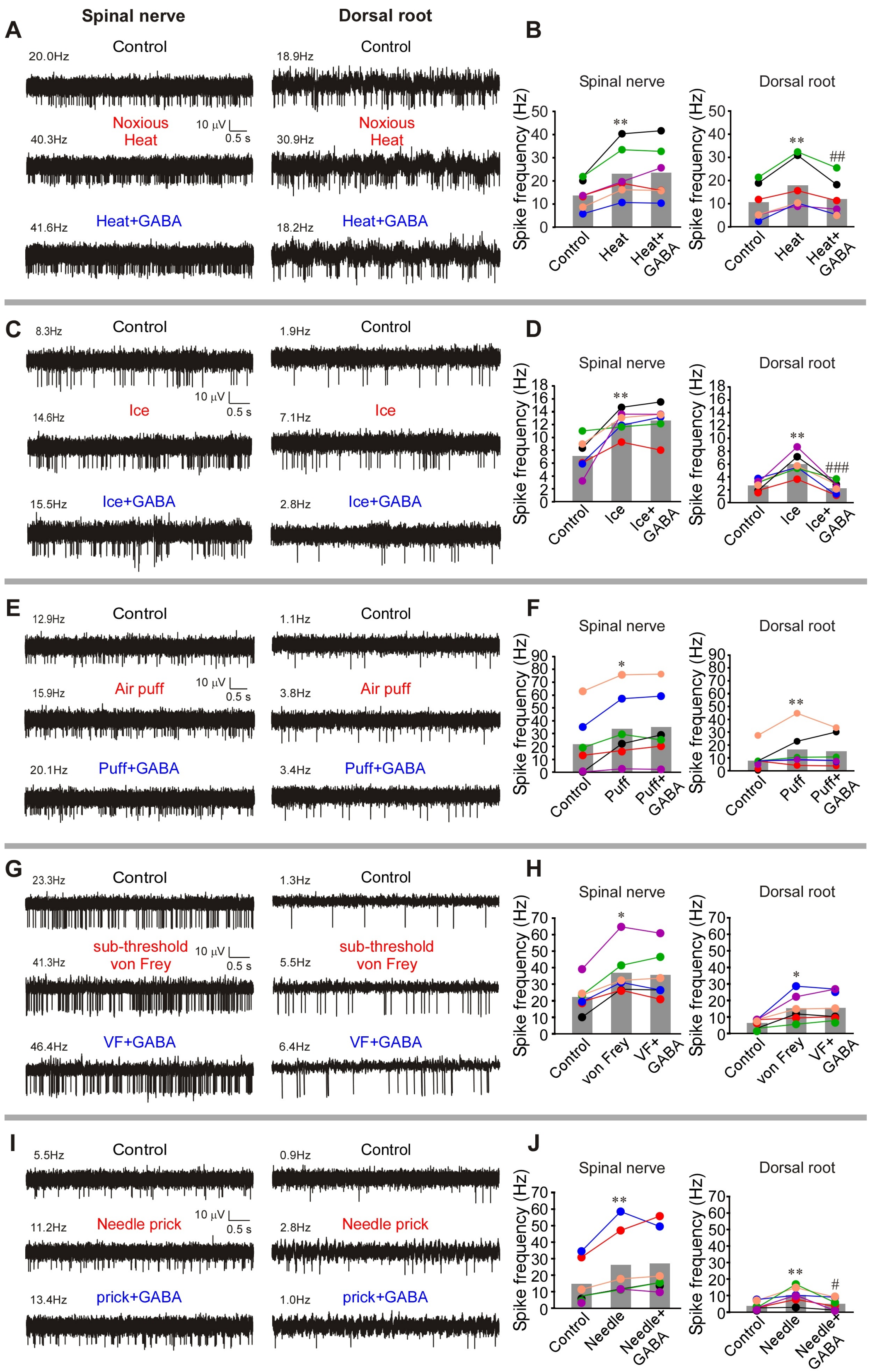

A
Spinal nerve
Dorsal root
Control
20.0Hz
18.9Hz
Noxious Heat
40.3Hz
30.9Hz
Heat+GABA
41.6Hz
18.2Hz
10 μV
0.5 s
B
Spinal nerve
Dorsal root
Spike frequency (Hz)
Control
Heat
Heat+ GABA
**
##
C
Control
8.3Hz
1.9Hz
Ice
14.6Hz
7.1Hz
Ice+GABA
15.5Hz
2.8Hz
D
Ice+ GABA
###
E
Control
12.9Hz
1.1Hz
Air puff
15.9Hz
3.8Hz
Puff+GABA
20.1Hz
3.4Hz
F
Puff
Puff+ GABA
*
G
Control
23.3Hz
1.3Hz
sub-threshold von Frey
41.3Hz
5.5Hz
VF+GABA
46.4Hz
6.4Hz
H
von Frey
VF+ GABA
I
Control
5.5Hz
0.9Hz
Needle prick
11.2Hz
2.8Hz
prick+GABA
13.4Hz
1.0Hz
J
Needle
Needle+ GABA
#

**S10 Fig. Activity induced by thermal and mechanical stimulation of the receptive fields is filtered at the DRG.** (**A**) Example of *in vivo* recording of the SN and DR activity (similar to these shown in Fig. 1). Stimulation of hindpaw of the rat with hot water (60oC) increased firing frequency in both SN and DR branches of the nerve (middle traces, as compared to basal activity shown in the upper traces). Application of GABA (200 μM, 3 μl) to DRG reduced heat-induced firing frequency in DR but not SN (bottom traces). (**B**) Summary for panel A. Two-factor (nerve site, treatment) repeated measures ANOVA: main effects associated with nerve site [F(1,10)=13.4; p<0.05) and treatment [F(2,9)=8.3; p<0.05]. Bonferroni post-hoc test: **significant difference from control (p<0.01); $^{\#\#}$significant difference from heat (p<0.01). (**C**) Similar to A and B but the hindpaw was stimulated with ice cube. (**D**) Summary for panel C. Two-factor (nerve site, treatment) repeated measures ANOVA: main effects associated with nerve site [F(1,10)=150.0; p<0.001) and treatment [F(2,9)=10.4; p<0.05]; significant interaction between nerve site and treatment [F(2,9)=13.6; p<0.05]. Bonferroni post-hoc test: **significant difference from control (p<0.01); $^{\#\#\#}$significant difference from ice (p<0.001). (**E**) Similar to A and B but the hindpaw was stimulated with air puff. (**F**) Summary for panel E. Two-factor (nerve site, treatment) repeated measures ANOVA: main effect associated with treatment [F(2,9)=7.4; p<0.05]. Bonferroni post-hoc test: $^{*,}$**significant difference from control (p<0.05, p<0.01). (**G**) Similar to A and B but the hindpaw was stimulated with sub-threshold von Frey filament (4g). (**H**) Summary for panel G. Two-factor (nerve site, treatment) repeated measures ANOVA: main effects associated with nerve site [F(1,10)=15.0; p<0.05] and treatment [F(2,9)=11.8; p<0.05]. Bonferroni post-hoc test: *significant difference from control (p<0.05). (**I**) Similar to A and B but the hindpaw was stimulated with a needle prick. (**J**) Summary for panel I. Two-factor (nerve site, treatment) repeated measures ANOVA: main effect associated with treatment [F(2,4)=18.0; p<0.01]; significant interaction between nerve site and treatment [F(2,4)=7.5; p<0.05]. Bonferroni post-hoc test: **significant difference from control (p<0.01); $^{\#}$significant difference from needle (p<0.05). Metadata for quantifications presented in this figure can be found at https://archive.researchdata.leeds.ac.uk/1042/
