# Supplementary material for: Dorsal root ganglia control nociceptive input to the central nervous system
Source: PLoS Biol. 2023 Jan 5;21(1):e3001958. doi: 10.1371/journal.pbio.3001958 (PMC9847955; doi:10.1371/journal.pbio.3001958)
Supplement: S11 Fig — (A, B) TTX blocks evoked spikes. Example traces of in vivo single unit recording from the DR aspect of a rat C fiber (A) or A fiber (B); stimulus electrode is placed in the spinal nerve. Parameters of stimulation and conduction velocity are indicated above the trace. TTX (1 μM, 3 μl; C) was injected into the DRG using a microsyringe at time points indicated by the bent arrow. Black circles indicate failed spikes. A fiber spike waveform for the A fiber recording is shown on the extended time scale within the dotted red box. (C, D) In the A-type fibers spike propagation through the DRG is not affects by GABA even at higher stimulation frequencies: 50 Hz (C) and 100 Hz (D). (E) Pie charts summarizing the percentage of C and A fibers in which TTX (from experiments shown in panels A and B) or GABA (data from the experiments shown in main Fig 5) produced a conduction block. (F) Spike failure rate before and during application of GABA to A fibers when stimulating at 50 and 100 Hz. No significant effects of GABA were found (n = 8; nonparametric Kruskal–Wallis ANOVA). Metadata for quantifications presented in this figure can be found at https://archive.researchdata.leeds.ac.uk/1042/. (PDF) [file pbio.3001958.s011.pdf]

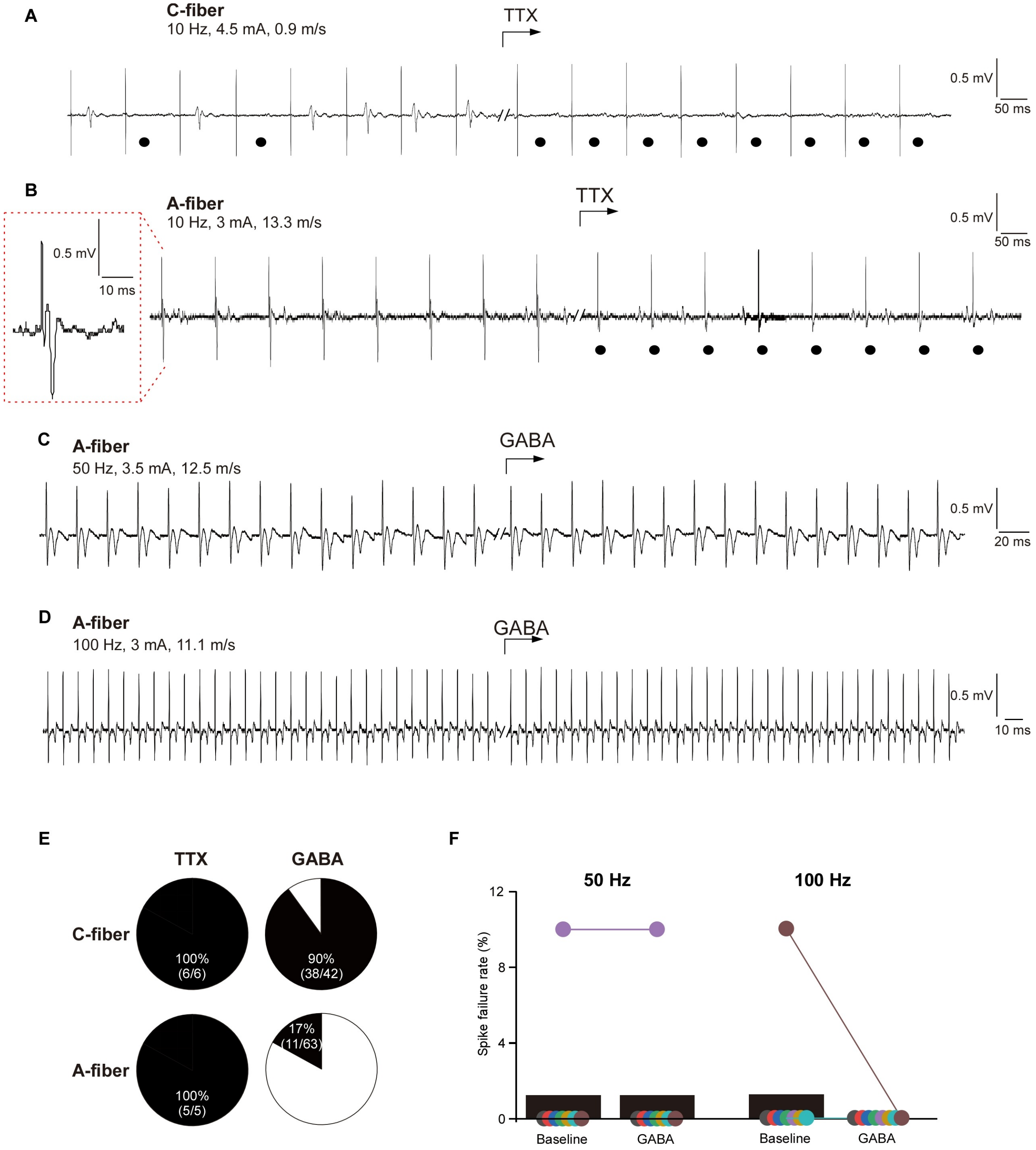

A
C-fiber
10 Hz, 4.5 mA, 0.9 m/s
TTX
0.5 mV
50 ms
B
A-fiber
10 Hz, 3 mA, 13.3 m/s
TTX
0.5 mV
10 ms
0.5 mV
50 ms
C
A-fiber
50 Hz, 3.5 mA, 12.5 m/s
GABA
0.5 mV
20 ms
D
A-fiber
100 Hz, 3 mA, 11.1 m/s
GABA
0.5 mV
10 ms
E
TTX
GABA
C-fiber
100%
(6/6)
90%
(38/42)
A-fiber
100%
(5/5)
17%
(11/63)
F
50 Hz
100 Hz
Spike failure rate (%)
12
8
4
0
Baseline
GABA
Baseline
GABA

S11 Fig. Additional single-unit recordings. (**A, B**) TTX blocks evoked spikes. Example traces of *in vivo* single unit recording from the DR aspect of a rat C fiber (A) or A fiber (B); stimulus electrode is placed in the spinal nerve. Parameters of stimulation and conduction velocity are indicated above the trace. TTX (1 μM, 3 μl; C) was injected into the DRG using a microsyringe at time points indicated by the bent arrow. Black circles indicate failed spikes. A fiber spike waveform for the A fiber recording is shown on the extended time scale within the dotted red box. (**C, D**). In the A-type fibers spike propagation through the DRG is not affects by GABA even at higher stimulation frequencies: 50 Hz (C) and 100 Hz (D). (**E**) Pie charts summarizing the percentage of C and A fibers in which TTX (from experiments shown in panels A and B) or GABA (data from the experiments shown in main Fig. 5) produced a conduction block. (F) Spike failure rate before and during application of GABA to A fibers when stimulating at 50 and 100 Hz. No significant effects of GABA were found (n=8; Nonparametric Kruskal-Wallis ANOVA). Metadata for quantifications presented in this figure can be found at https://archive.researchdata.leeds.ac.uk/1042/
