# Supplementary material for: Dorsal root ganglia control nociceptive input to the central nervous system
Source: PLoS Biol. 2023 Jan 5;21(1):e3001958. doi: 10.1371/journal.pbio.3001958 (PMC9847955; doi:10.1371/journal.pbio.3001958)
Supplement: S12 Fig — Light-sheet microscopy of cleared rat DRG; axon lengths (A), stem diameter (B), and somatic diameter (C) of the peripherin- and NF-200-labeled fibers were analyzed per animal (see also Fig 6C–6E). (A) Peripherin: 3 animals (rat 1: 5 DRGs, 36 stems; rat 2: 2 DRGs, 9 stems; rat 3: 3 DRGs, 12 stems). NF200: 3 animals (rat 1: 4 DRGs, 22 stems; rat 2: 1 DRG, 3 stems; rat 3: 2 DRGs, 5 stems); paired t test: t(2) = 3, p < 0.05. (B) Peripherin: 3 animals (rat 1: 5 DRGs, 36 stems; rat 2: 2 DRGs, 9 stems; rat 3: 3 DRGs, 12 stems). NF200: 3 animals (rat 1: 4 DRGs, 22 stems; rat 2: 1 DRG, 3 stems; rat 3: 2 DRGs, 5 stems); paired t test: t(2) = 3, p < 0.05. (C) Peripherin: 3 animals (rat 1: 5 DRGs, 150 somas; rat 2: 2 DRGs, 122 somas; rat 3: 3 DRGs, 155 somas). NF200: 3 animals (rat 1: 4 DRGs, 150 somas; rat 2: 1 DRG, 31 somas; rat 3: 2 DRGs, 75 somas); paired t test: t(2) = 7, p < 0.05. Metadata for quantifications presented in this figure can be found at https://archive.researchdata.leeds.ac.uk/1042/. (PDF) [file pbio.3001958.s012.pdf]

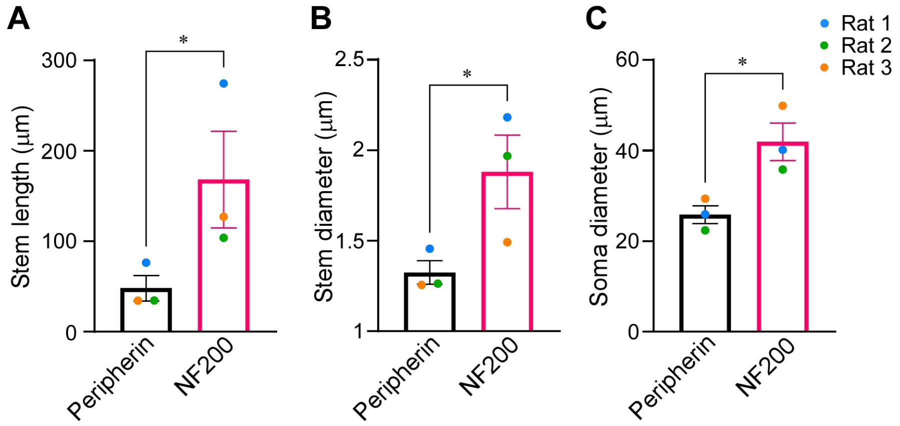

S12 Fig. Additional analysis of initial axon morphology. Light-sheet microscopy of cleared rat DRG; axon lengths (**A**), stem diameter (**B**) and somatic diameter (**C**) of the peripherin- and NF-200-labelled fibers were analyzed per animal (see also Figure 6C-E). (A) peripherin: 3 animals (rat 1: 5 DRGs, 36 stems; rat 2: 2 DRGs, 9 stems; rat 3: 3 DRGs, 12 stems). NF200: 3 animals (rat 1: 4 DRGs, 22 stems; rat 2: 1 DRG, 3 stems; rat 3: 2 DRGs, 5 stems); paired t-test: $t(2)=3$, $p<0.05$. (B) peripherin: 3 animals (rat 1: 5 DRGs, 36 stems; rat 2: 2 DRGs, 9 stems; rat 3: 3 DRGs, 12 stems). NF200: 3 animals (rat 1: 4 DRGs, 22 stems; rat 2: 1 DRG, 3 stems; rat 3: 2 DRGs, 5 stems); paired t-test: $t(2)=3$, $p<0.05$. (C) peripherin: 3 animals (rat 1: 5 DRGs, 150 somas; rat 2: 2 DRGs, 122 somas; rat 3: 3 DRGs, 155 somas). NF200: 3 animals (rat 1: 4 DRGs, 150 somas; rat 2: 1 DRG, 31 somas; rat 3: 2 DRGs, 75 somas); paired t-test: $t(2)=7$, $p<0.05$. Metadata for quantifications presented in this figure can be found at https://archive.researchdata.leeds.ac.uk/1042/
