# Supplementary material for: Dorsal root ganglia control nociceptive input to the central nervous system
Source: PLoS Biol. 2023 Jan 5;21(1):e3001958. doi: 10.1371/journal.pbio.3001958 (PMC9847955; doi:10.1371/journal.pbio.3001958)
Supplement: S13 Fig — (A) C-fiber model parameter space analysis. Fraction of available TTX-sensitive Na channels at the T-junction (TJ) in response to steady-state GABAA receptor activation. For a model with GABAA receptors restricted to the soma, varying the diameter of the stem axon (length 75 μm; top left panel) or varying stem axon length (diameter 1.35 μm; top right panel) generally had only modest effects on the fraction of available Na channels (i.e., proportion of non-inactivated Na channels). In contrast, expressing GABAA receptors on the axons, compared with the soma alone, substantially decreased Na channel availability (stem length 75 μm, stem diameter 1.35 μm; bottom left). Connection of the soma and stem to the TJ made no difference when GABAA receptors were expressed across all compartments, (bottom right). (B) A-fiber model parameter space analysis. With GABAA receptors restricted to the soma, varying stem axon diameter (stem length 400 μm) or length (stem diameter 2 μm) substantially affected the available fraction of Na channels (top left and right panels). Likewise, if GABAA receptors were expressed both in the soma/axon initial segment and all nodes of Ranvier, rather than just the soma/AIS alone, fractional availability of Na channels was substantially reduced by GABAA receptor activation (bottom left). Connection of the soma and stem axon to the TJ provided for a greater effect of GABAA receptor activation compared to when detached, although this effect was less pronounced with at higher densities of GABAA receptor activation (bottom right panel). Metadata for quantifications presented in this figure can be found at https://archive.researchdata.leeds.ac.uk/1042/. Code can be found at GitHub (https://github.com/dbjaffe67/DRGsims). (PDF) [file pbio.3001958.s013.pdf]

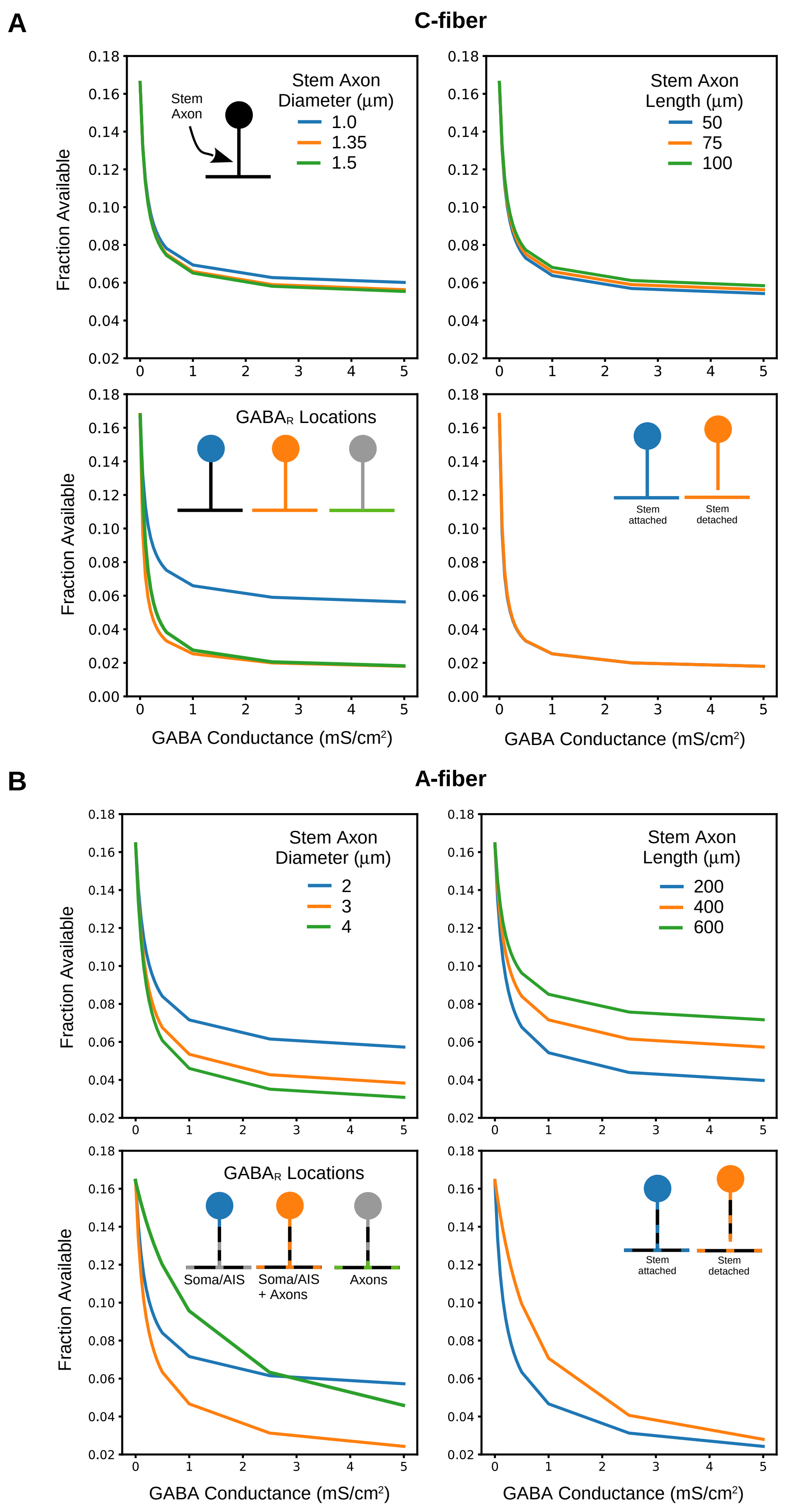

A
C-fiber
Stem Axon
Stem Axon Diameter (µm)
1.0
1.35
1.5
Stem Axon Length (µm)
50
75
100
GABA_R Locations
Stem attached
Stem detached
Fraction Available
GABA Conductance (mS/cm²)
B
A-fiber
Stem Axon Diameter (µm)
2
3
4
Stem Axon Length (µm)
200
400
600
GABA_R Locations
Soma/AIS
Soma/AIS + Axons
Axons
Stem attached
Stem detached
Fraction Available
GABA Conductance (mS/cm²)

S13 Fig. Parameter space analysis. (**A**), C-fiber model parameter space analysis. Fraction of available TTX-sensitive Na channels at the T-junction (TJ) in response to steady-state $GABA_A$ receptor activation. For a model with $GABA_A$ receptors restricted to the soma, varying the diameter of the stem axon (length 75 μm; top left panel) or varying stem axon length (diameter 1.35 μm; top right panel) generally had only modest effects on the fraction of available Na channels (i.e. proportion of non-inactivated Na channels). In contrast, expressing $GABA_A$ receptors on the axons, compared with the soma alone, substantially decreased Na channel availability (stem length 75 μm, stem diameter 1.35 μm; bottom left). Connection of the soma and stem to the TJ made no difference when $GABA_A$ receptors were expressed across all compartments, (bottom right). (**B**) A-fiber model parameter space analysis. With $GABA_A$ receptors restricted to the soma, varying stem axon diameter (stem length 400 μm) or length (stem diameter 2 μm) substantially affected the available fraction of Na channels (top left and right panels). Likewise, if $GABA_A$ receptors were expressed both in the soma/axon initial segment (AIS) and all nodes of Ranvier, rather than just the soma/AIS alone, fractional availability of Na channels was substantially reduced by $GABA_A$ receptor activation (bottom left). Connection of the soma and stem axon to the TJ provided for a greater effect of $GABA_A$ receptor activation compared to when detached, although this effect was less pronounced with at higher densities of $GABA_A$ receptor activation (bottom right panel). Metadata for quantifications presented in this figure can be found at https://archive.researchdata.leeds.ac.uk/1042/
Code can be found at GitHub (https://github.com/dbjaffe67/DRGsims).
